# European Guideline for the Management of Kidney Transplant Patients With HLA Antibodies: By the European Society for Organ Transplantation Working Group

**DOI:** 10.3389/ti.2022.10511

**Published:** 2022-08-10

**Authors:** Nizam Mamode, Oriol Bestard, Frans Claas, Lucrezia Furian, Siân Griffin, Christophe Legendre, Liset Pengel, Maarten Naesens

**Affiliations:** ^1^ Department of Transplantation, Guys Hospital, London, United Kingdom; ^2^ Department of Nephrology and Kidney Transplantation, Vall d’Hebrón University Hospital, Barcelona, Spain; ^3^ Department of Immunology, Leiden University Medical Center, Leiden, Netherlands; ^4^ Department of Immunology, University of Antwerp, Antwerp, Belgium; ^5^ Kidney and Pancreas Transplantation Unit, Department of Surgical Gastroenterological and Oncological Sciences, University Hospital of Padua, Padua, Italy; ^6^ Department of Nephrology, University Hospital of Wales, Cardiff, United Kingdom; ^7^ Department of Nephrology and Adult Kidney Transplantation, Hôpital Necker and Université de Paris, Paris, France; ^8^ Centre for Evidence in Transplantation, University of Oxford, Oxford, United Kingdom; ^9^ Department of Microbiology, Immunology and Transplantation, KU Leuven, Leuven, Belgium

**Keywords:** kidney transplantation, guidelines, HLA antibodies, sensitization, incompatible

## Abstract

This guideline, from a European Society of Organ Transplantation (ESOT) working group, concerns the management of kidney transplant patients with HLA antibodies. Sensitization should be defined using a virtual parameter such as calculated Reaction Frequency (cRF), which assesses HLA antibodies derived from the actual organ donor population. Highly sensitized patients should be prioritized in kidney allocation schemes and linking allocation schemes may increase opportunities. The use of the ENGAGE 5 ((Bestard et al., Transpl Int, 2021, 34: 1005–1018) system and online calculators for assessing risk is recommended. The Eurotransplant Acceptable Mismatch program should be extended. If strategies for finding a compatible kidney are very unlikely to yield a transplant, desensitization may be considered and should be performed with plasma exchange or immunoadsorption, supplemented with IViG and/or anti-CD20 antibody. Newer therapies, such as imlifidase, may offer alternatives. Few studies compare HLA incompatible transplantation with remaining on the waiting list, and comparisons of morbidity or quality of life do not exist. Kidney paired exchange programs (KEP) should be more widely used and should include unspecified and deceased donors, as well as compatible living donor pairs. The use of a KEP is preferred to desensitization, but highly sensitized patients should not be left on a KEP list indefinitely if the option of a direct incompatible transplant exists.

## Introduction

Although kidney transplantation rates have increased in many countries in recent years, highly sensitized patients typically spend longer waiting for a transplant, or may never receive one. This guideline is aimed at healthcare professionals who are faced with a patient with HLA antibodies, to provide advice regarding the most appropriate way to achieve a successful transplant.

The guideline does not include patients undergoing non-renal or multi-organ transplants, and does not consider pediatric recipients in detail.

This article provides a summary of the guideline; the full guideline can be accessed at: https://esot.org/wp-content/uploads/2022/07/WS06_Full-doc_07202022.pdf.

## Methods

A working group (WS06) was convened by the European Society of Transplantation (ESOT) as part of the Transplant Learning Journey Project, including healthcare professionals from across Europe with expertise in the field, patient group representatives and a member of the Centre for Evidence in Transplantation, University of Oxford, United Kingdom.

Six areas of interest were defined and are listed below:

• Definition of sensitization
• Comparison of practices across Europe for transplanting sensitized patients
• The place of kidney exchange programs for sensitized patients
• Desensitization strategies
• Outcomes after HLA incompatible transplantation
• Strategies for access to kidney transplantation for highly sensitized patients

For each, a standard systematic search strategy was predefined, using the PICO model to formulate clinical questions. Bibliographic searches were developed for each of the clinical questions by experienced staff from the Centre for Evidence in Transplantation. Systematic searches were conducted in the Transplant Library (www.transplantlibrary.com), Medline and Embase and consisted of a mixture of free text and controlled vocabulary terms.

Different members of the working group drafted each chapter, which was then reviewed by the whole working group. The initial recommendations were presented at an ESOT webinar open to all, on the 29th June 2021, and again *via* an ESOT Twitter chat on the 2nd August 2021, after which further refinements were made. An Expert Working Group, including interested healthcare professionals from across Europe, was convened on the 28th August 2021 (in Milan and online), when a draft of the final document was presented and discussed, with further refinements following this.

The detailed methodology, including the search strategies used and search dates, is presented in the full guideline (Appendix).

We have presented below a brief summary of each chapter listed above, along with our recommendations. Recommendations were graded according to the strength of the recommendation [strong (1) or weak (2)] and the quality of the evidence [high (A), moderate (B), low (C) or very low (D) (2)].

## Definition of Sensitization

High levels of donor-specific HLA antibodies (DSA) present at transplantation are associated with a high incidence of hyper-acute rejection (3,4), and can be induced by previous blood transfusions, pregnancies or transplants (5–7).

Historically, complement-dependent cytotoxicity (CDC) was the gold standard measure of HLA antibodies and the degree of sensitization was expressed as a percentage of panel reactive antibodies (%PRA). This %PRA was defined by the percentage of panel donors reactive with the patient serum in CDC. The %PRA was a relatively inaccurate assessment of sensitization, but often a PRA > 85% was considered the threshold for a highly sensitized patient (8).

A CDC crossmatch only detects complement-activating HLA antibodies. To also detect the non-complement fixing IgG subclasses IgG2 and IgG4, the Flow Cytometric crossmatch (FCM) was introduced in several laboratories (9,10). Donor-specific antibodies detectable in FCM, but not in CDC, appeared to be clinically relevant and were associated with graft rejection and graft loss in a proportion of recipients (11). In contrast to CDC reactive DSAs, antibodies detected in FCM were considered more as a risk factor than a contra-indication for transplantation.

Clinically irrelevant antibodies (including autoantibodies) reactive with other structures on lymphocytes can interfere in the outcome of both a CDC and a FCM crossmatch (12,13) leading to false positive results. Additionally, endothelial cells in the kidney can express alloantigens, which are not present on lymphocytes (14) and antibodies to these cannot currently be detected.

Solid phase assays were introduced more recently (15). Single antigen beads (SAB) have facilitated the detection and identification of specific HLA antibodies (16,17). Patient serum is tested against a mix of about one hundred different beads, each covered with HLA molecules of the same specificity. The degree of antibody binding to a specific bead is expressed as mean fluorescence intensity (MFI). This assay appears to be far more sensitive than CDC and FCM for detecting HLA antibodies and DSA. As a consequence, the proportion of sensitized patients has significantly increased after the introduction of solid phase assays (18).

The clinical relevance of antibodies detectable in SAB assays is still a matter of debate (19). Individual centers have tried to make correlations between the already established clinical relevance of CDC and FCM and the MFI values obtained in SAB (20).

Although no absolute thresholds can be defined, it is generally accepted that the highest MFI values predict a positive CDC crossmatch, although exceptions exist as some high MFIs are associated with a negative CDC (21). As the SAB assay is very sensitive, positive reactions are obtained, usually with a lower MFI, which do not correlate with a positive FCM or CDC crossmatch. The clinical value of such antibodies has been extensively studied with some conflicting results (22,23).

Most centers use a cut-off MFI of 1,000–1,500 (21) but there is no general agreement on this value. HLA antibodies are directed against specific epitopes expressed on the target HLA antigen, but individual epitopes can be shared by (many) different HLA alleles (24), which may lead to differing MFI for the same antibody. These, and other issues, can make it difficult to determine the clinical significance of a given antibody.

Recently, an attempt has been made to introduce more reliable parameters for the definition of the degree of sensitization based on the antibody specificities present in the patient and the HLA phenotypes of the actual organ donor population. Different names are now circulating for this novel parameter: vPRA (virtual PRA) (25), cPRA (calculated PRA) (26) and cRF (calculated reaction frequency) (27) but they all reflect the chance that a patient has HLA antibodies reactive with a donor derived from the actual organ donor population. HLA incompatible transplantation (HLAi) is defined by a positive CDC or FCM crossmatch at baseline, since we believe that desensitization is only required in these cases. If these crossmatches are not routinely performed, centers are advised to define locally MFI values corresponding to positive CDC or flow crossmatches to be used for the selection of patients to be desensitized.

### Recommendations

• A parameter, which is based on the HLA frequencies of the actual organ donor population, such as vPRA, cPRA or cRF, should be used to estimate the chance that a sensitized patient can be transplanted with a compatible donor without the need for any special treatment (1C).
• When defining unacceptable mismatches in highly sensitized patients on the basis of (weak) antibody reactivities in single antigen bead assays only, one should consider the poorly defined risk of antibody-mediated rejection (ABMR) in the light of a prolonged waiting time and associated mortality and morbidity (2D).

#### Areas for Further Research

• Further standardization of single antigen bead assays and their interpretation is recommended (1C).
• Better HLA matching on the basis of antibody epitopes rather than antigens and a restricted transfusion policy will probably diminish the number of highly sensitized patients, but more data are needed.

## Comparison of Practices Across Europe for Transplanting Sensitized Patients

Both deceased and living donations are coordinated on either a national basis, or on behalf of a group of countries (http://www.accord-ja.eu/background). Eurotransplant (https://www.eurotransplant.org/) and Scandiatransplant (http://www.scandiatransplant.org/) each allocate donor organs for groups of countries. Larger donor pools would be expected to increase the likelihood of identifying a compatible donor for those who are hard to match. A survey of transplant practices around Europe was carried out during September and October 2021 for the purposes of this guideline, and the results form Table 1.

**TABLE 1:** Informal European survey of practices regarding transplantation, 2021.

Country or organization for deceased donor allocation	Population (million)	Living donation	Deceased donation			
		Is there access to a kidney exchange program?	Does the allocation scheme include prioritization for sensitized recipients?	Does the allocation scheme include an acceptable mismatch program?	Details	
Eurotransplant (Austria, Belgium, Croatia, Germany, Hungary, Luxembourg, Netherlands, Slovenia)	137	Yes: Austria (with the Czechia and Israel), Belgium (20), Netherlands	Yes	Yes	Acceptable antigens are defined by the lack of antibody-reactivity in complement-dependent cytotoxicity assays using target cells mismatched for a single HLA antigen, or single antigen-expressing cell lines	
ScandiaTransplant (Denmark, Finland, Iceland, Norway, Sweden, Estonia)	28.9	ScandiaTransplant Kidney Exchange Program launched April 2019	Yes	Yes, ScandiaTransplant Acceptable Mismatch Program (STAMP)^a^	Common waiting list and database system. STAMP patients have the highest priority for a deceased donor kidney	
Czechia	10.7	Yes	Yes	No	Patients are categorized according to their measured PRA: 0%–20%, 20%–80, and >80%, with higher priority for transplantation given to those with higher PRA values. Patients who have waited longer than 3 years for a transplant are prioritized, regardless of their PRA value	
		Recent expansion to include Austria and Israel			DSA are allowed, based on local protocols for desensitization	
France	67	Yes	Yes	Yes	Sensitized patients are prioritized according to waiting time and HLA compatibility	
Greece	10.4	Yes	Yes	Yes	Patients are prioritized based on waiting time and HLA mismatch	
Ireland	5	Yes—with the United Kingdom	Yes	Yes	All highly sensitized patients who are long waiting are screened to identify acceptable mismatches or windows in which they can be transplanted	
Italy	60.3	Yes	Yes	Yes	The Italian national allocation scheme prioritizes at national level patients with PRA >90% and who have been on dialysis >8 years	
					Recipients are selected according to a points score, based on	
					- PRA	
					- Age mismatch between donor and recipient	
					- Recipient age	
					- HLA mismatch	
					- Time spent on dialysis	
					- Time on waiting list	
Latvia	1.9	Yes (21)				
Lithuania	2.9	Yes (22) established in 2013, although up to 2019, the system has not been used			Although Lithuania is not a member of international organ procurement and allocation organizations yet, they do collaborate with neighboring Nordic countries and exchange organs with Latvia, Estonia and Poland	
Poland	38	Yes	Yes	Yes	Prioritization for patients with a PRA >80%; increased weighting for patients with PRA 50–79	
Portugal	10.2	Yes	Yes	No	Additional points for sensitized and highly sensitized patients	
Russia	146.2		NoEach transplant center has their own internal protocol	YesSome kidney centers may transplant if there is an acceptable mismatch	There is no common waiting list in Russia or any kind of program like Eurotransplant. Each center has its own waiting list, their own algorithm for prioritizing patients for transplantation (although many use UNOS, Intermax or other classification systems to help decisions) and their own protocol for post-transplant follow-up	
					Prioritization is based on donor and recipient risk index match, waiting time, and HLA mismatch	
Slovakia	5.4	No	No	No		
Spain	46.8	Yes	Yes	No	One kidney of all brain death donors is offered to a National Prioritization Scheme for sensitized patients with a cPRA >98%. Kidney acceptance for an individual patient based on virtual crossmatch (23)	
Switzerland	8.74	Yes	Yes	Yes	Prioritization for allocation is based on a continuum of increasing cPRA for each blood group. An MFI cut-off of 1,000 is used for both class 1 and class 2 DSA	
Turkey	85.6	Yes	No	No	Allocation is according to a scoring system	
					Criteria	Score
					HLA match	DR 150, B 50, A 5
					Region	1000
					Center	250
					Recipient age (<11 years / 12–17/≥18 years	HLA match score multiplied by 2.5/1.5/1
					Time on dialysis	3 points for each month
United Kingdom	68	Yes	Yes	No	Absolute priority for those with cRF >100%, matchability score 10, waiting time >7 years	
					Remaining patients prioritized on points score, based on	
					i. Donor and recipient risk index match	
					ii. Waiting time	
					iii. HLA mismatch	
					iv. Local region > non-local regions (of four national regions)	

a http://www.scandiatransplant.org/organ-allocation/Manual_STAMP_20_nov_2017_version_8.1.pdf.

http://www.scandiatransplant.org/organ-allocation/Kidney_exchange_11_november_2020.pdf.

Deceased donor offering schemes can adjust for the increased waiting time of sensitized patients, either by increasing the weighting given to those who are hard to match, as in the UK Kidney Offering Scheme (https://www.odt.nhs.uk/transplantation/kidney/kidney-offering-and-matching/) or by the development of an Acceptable Mismatch (AM) program (28). Enrolment in an AM program is reserved for those more highly sensitized patients, whose chance of receiving an offer is otherwise low. For example, to be considered for enrolment in the Eurotransplant AM program, recipients will have been receiving dialysis for at least 2 years and have a PRA of >85%. The Eurotransplant AM program has enabled successful transplantation of highly sensitized patients with excellent outcomes (29).

The EUROSTAM project has compared data from five European registries to determine whether expanding the donor pool across different populations will result in increased rates of transplantation for those with >95% sensitization (27). In total, 195 (27%) of the 724 highly sensitized patients who had been registered for at least 5 years at each organization had an increased chance of a compatible kidney transplant offer in a different European pool. This makes a strong case for sharing kidneys between European countries and registries for selected difficult to transplant patients.

Kidney exchange programs (KEP) in Europe began in Switzerland in 1999 (30), and the Dutch and UK schemes were initiated in 2004 and 2007 respectively (31,32); the latter has performed the greatest number of transplants (33). Over the last decade, programs have been established throughout Europe (33). Approaches to exchange schemes vary; altruistic donation is permitted in the United Kingdom, but is not possible in France, Poland, Greece or Switzerland. Similarly, compatible pairs are included in the United Kingdom, but not in France or Portugal (33). The European Network for Collaboration on Kidney Exchange Programs (ENCKEP, https://www.eurotransplant.org/) was established in 2016. The program has contributed to aspirations for future developments, including modelling of European KEPs with the aim of future optimization (34).

No European country has a published national consensus on their optimal recommended management pathway for highly sensitized patients, although several European centers have published their protocols and outcomes following HLAi transplantation (35–38). The survey referred to above demonstrated substantial variability in the definition of sensitization, approaches to improve opportunities for deceased and living transplantation and perceived success of HLAi transplantation.

### Recommendations

#### Organ Allocation

• We recommend an active policy of prioritizing highly sensitized patients for organ transplantation, using cPRA/cRF (1C).

## The Place of Kidney Exchange Programs for Highly Sensitized Patients

**FIGURE 1:**
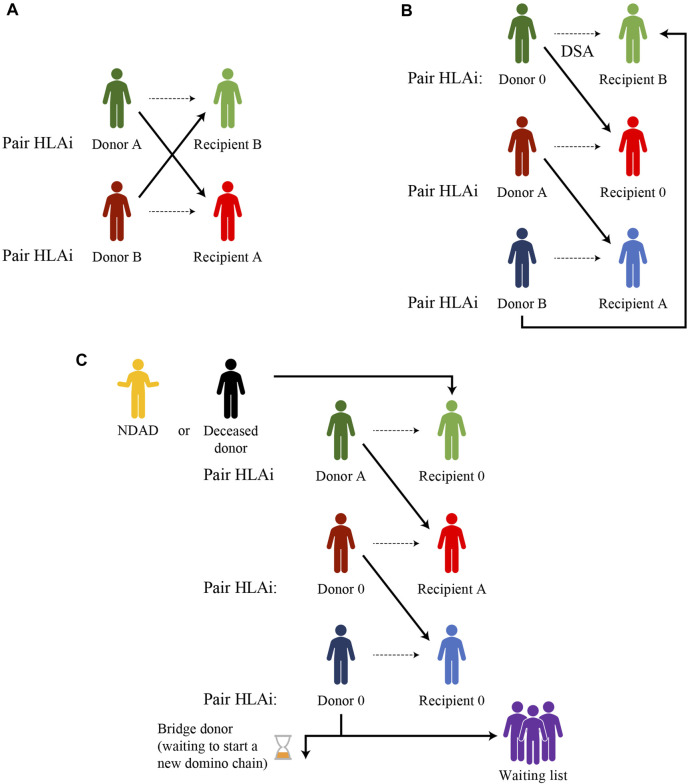
Examples of kidney paired donation exchanges **(A)** Two-way exchange **(B)** Three-way exchange **(C)** Domino-chain ending with a donation to a wait-list patient or a bridge donor and starting from a non-directed altruistic donor (NDAD), a non-simultaneous extended altruistic donor (NEAD), or a deceased donor (Dec-K program).

The simplest form of a KEP is a two-way exchange involving two incompatible pairs who swap their donors to achieve a compatible transplant for both recipients (Figure 1). The closed loop between three or more incompatible pairs whose recipients find a compatible kidney by exchanging their donors, represents another basic form of kidney paired donation.

Unfortunately, for highly sensitized patients with a wide range of anti-HLA antibodies or for blood type O recipients, it is very hard to find a compatible match for each pair involved in a closed loop.

The option of a non-directed altruistic (or unspecified) donor (NDAD) who is willing to donate his/her kidney with no intended recipient, avoids the need to “close the loop.” The NDAD’s kidney is matched with the recipient of an incompatible pair whose living donor donates to another incompatible recipient, initiating a domino-paired kidney exchange. The chain ends with the donor of the last pair donating to a recipient on the waiting list or waiting for another suitable match, starting another sequence of paired donations later (non-simultaneous extended altruistic donor chain), thus becoming a bridge donor. This model is potentially associated with an incremental risk of donor reneging. The occurrence of broken chains has been reported to be as low as 1.5%, with the most common causes for broken chains being bridge donor medical issues (0.46%), donors electing not to proceed (0.34%) and broken chains resulting from the kidney being declined by the recipient surgeon (0.23%) (39).

The first deceased donor-initiated chain was reported by Furian, et al in 2019 (40). In the DECeased donor kidney paired exchange (DEC-K) program, the chain-initiating kidney, selected from the deceased donor pool, is allocated to a recipient with an incompatible living donor and, at the end of the domino-chain, the living donor of the last pair donates to a waiting list patient. The major advantage of the DEC-K program is the ability to offer transplantation to recipients of incompatible donor pairs, but it also benefits waiting list candidates by allocating chain-ending kidneys from a living donor to them, prioritizing sensitized patients and those who have waited a long time for immunological reasons.

List exchange is another form of KEP, proposed by Delmonico et al. (41), to prevent the issue of donor reneging. In this scheme, the donor of the incompatible pair donates before the recipient has received their compatible transplant from the deceased donor pool but, after donation, the paired recipient acquires priority over the WL candidates.

Other novel KEP schemes take place in the setting of “chronological incompatibility” and constitute the advanced donation programs where a living donor donates his/her kidney at his/her convenience to a recipient of an incompatible pair in need of transplant while his/her intended recipient will receive the reciprocal compatible kidney later on, when he/she actually needs a transplant (42).

ABO or HLA compatible pairs may also be included in a KEP, in order to increase the pool and provide benefits (such as better age or HLA matching) for the compatible recipient. A recent report from the National Kidney Registry linked to data from the Scientific Registry of Transplant Recipients identified 154 compatible pairs involved in kidney exchange programs, seeking to improve their HLA matching through an exchange. These patients obtained a transplant from younger donors, with higher estimated glomerular filtration rate and body mass index and a better score on the living kidney donor profile index as compared with their original donor (43).

Another strategy to improve results is combining exchange programs with desensitization. ABO incompatible transplantation in the absence of DSA provides excellent transplantation results, so ABO incompatible living donors against whom recipients have lower anti-blood group antibody titers can be included in a KEP. This strategy has been successfully applied in the Australian program and at the John Hopkins Institute (44,45).

Trans-organ paired exchange represents the most innovative concept of KEP. It might be used, for example, when a living kidney donor who is not eligible for renal donation but can donate his/her liver to a liver recipient of a pair whose donor is ruled out from liver donation but is suitable for kidney donation. Torres, et al published the first case of trans-organ exchange, attracting many criticisms related to the surgical risk of donation that is very different for different organs (46).

### KEP Versus Desensitization

In 2005, Segev, et al. (47) showed, by a simulation based on UNOS data, the superiority of KEP over desensitization, guaranteeing better graft outcomes and higher transplantation rates for HLAi pairs. The authors clearly stated that KEP should be the preferred treatment for patients who have HLA incompatibilities with their willing donors.

However, despite the implementation of KEP strategies, in the United States, patients with a PRA of 99.9% remain the most disadvantaged transplant candidates with prolonged waiting times and high waiting list mortality (48). In fact, patients with a cPRA >80% were less likely to receive a living-donor kidney transplant (6.5%) compared with candidates with a cPRA <80% (26.7%), and in the 99% cPRA group, only 3.4% of all transplants were from a kidney paired donor, and only 1.3% in 100% cPRA candidates. This is why some transplantation centers still promote desensitization as a valid and needed approach to increase the probability of transplantation in highly sensitized patients (49). Others have proposed KEP only in cases of failed desensitization procedures, as a kind of “rescue” therapy (50).

### Recommendations

• Access to the donor pool should be increased through greater use of:
  - Increased access to and harmonization of kidney exchange programs with greater and standardized sharing of outcomes (1C)
  - Inclusion of unspecified kidney donations (if these are performed) in kidney exchange programs (1C)
  - Inclusion of compatible pairs and deceased donor organs in kidney exchange programs (1C)
• Entry into a kidney exchange program is the preferred initial option over desensitization given the better transplant outcomes and cost-effectiveness, unless there is a need for desensitization, there is clinical urgency or a low chance of a transplant (1C).

## Desensitization Strategies in Kidney Transplantation

If the strategies listed above have not yielded, or are unlikely to yield, a transplant, desensitization may be considered. There are several ways to desensitize HLA-immunized patients.

In a randomized trial (51), it was shown that IVIgs alone allowed more patients to be transplanted, but the overall benefit was still quite limited. It is relatively simple to decrease the global level of IVIgs through plasma exchange or by immune-adsorption—an equivalent method. The number of plasma exchanges necessary to lower the IgG level is about five and the gain of increasing the number of plasma exchanges beyond that is small (44).

Rituximab (anti-CD20) can be used to desensitize patients prior to transplantation. This drug aims to decrease the rebound effect linked to decreased levels of immunoglobulins in the plasma. Efficacy is monitored using the expression of CD19 on B cells. Currently, the two methods used to desensitize patients are either a combination of anti-CD20 antibodies and high-dose IVIgs (2 g/kg over 2–4 days) (52), or a combination of 3–5 sessions of plasma exchange followed after each session by an infusion of low-dose IVIgs (0.1 g/kg) to avoid rebound (44). New anti-CD20 monoclonal antibodies (such as ocrelizumab or obinutuzumab) may be more efficient, as well as anti-CD19 antibodies.

It is possible to decrease the synthesis of proteins (DSAs) using proteasome inhibitors such as the first-generation drug, bortezomib. This drug was tested in a study with such a complex design (including the testing of many other drugs) that it is difficult to clearly see its role in desensitization (53). Second generation drugs such as carfilzomib or ixazomib may be more efficient.

A logical approach to desensitization is to block the activity of complement in order to decrease the effect of antibodies such as DSAs. The anti-C5 monoclonal antibody, eculizumab, was the first to be tested in this indication. A randomized study was designed for living donor recipients and compared the use of eculizumab for 3 months post-transplantation with a control group who received desensitization (38). Unfortunately, the results were rather disappointing, with no significant difference found between the two groups. One explanation of these results is the difficulties in defining ABMR and probably more importantly, the use of anti-C5 in patients with DSAs not fixing the complement (54). In contrast, in a study in sensitized patients being transplanted with an organ from a deceased donor, it was possible to get a low incidence of ABMR using eculizumab. However, there were no controls in this study, so the overall results are not clear-cut, but it remains a logical approach that may be used in selected groups of patients. Other complement blockers (such as a C1-inhibitor) are the subject of current clinical trials (55).

Another approach, which is not strictly desensitization, is the use of a cysteine protease (IG endopeptidase, Ides, Imlifidase and Idefirix^®^). Imlifidase is currently the only approved therapy for use in the EU for desensitization treatment of highly sensitized adult kidney transplant patients with a positive crossmatch against an available deceased donor. It cleaves all IgGs, both intra- and extra-vascularly, without regard to their specificity, with an immediate action that lasts around 5–7 days; this drug cannot be re-dosed due to immunogenicity (56). It is important to stress that there is an anti-HLA antibodies rebound when the activity of the drug disappears, rebound that explains the frequency of ABMR. Imlifidase has been used in HLAi hyper-immunized patients with good and safe results and at 3 years, crossmatch positive patients who were converted to negative with imlifidase to enable transplantation had ABMR with a frequency equivalent to other desensitization methods. Three years after imlifidase-enabled desensitization and transplantation, the death-censored allograft survival was 84%, patient survival 90%, and mean eGFR was 55 ml/min/1.73 m^2^ (49 ml/min/1.73 m^2^ for those with ABMR and 61 ml/min/m^2^ for those without ABMR) (57).

An additional desensitization strategy is the manipulation of the cytokines involved in B cell activation. In this indication, tocilizumab, an anti-IL6 receptor monoclonal antibody has been giving promising results in a randomized trial, used in addition to current desensitization protocols (58). Antibodies to anti-IL6 have been studied in a randomized clinical trial showing promising efficacy regarding decreased DSA, less eGFR decline as well as changes in biopsies features but also a careful evaluation of safety data (diverticular complications) (59). Belimumab, an anti-BAFF monoclonal antibody, might be a useful adjunct to standard care immunosuppression in renal transplantation patients, as it shows no major increased risk of infection and potential beneficial effects on humoral alloimmunity (60).

### Recommendations

• The most efficacious desensitization strategy is to start with rounds of plasma exchanges/immunoadsorption together with IVIG or B-cell depletion with anti-CD20 monoclonal antibodies (1C).

#### Areas for Further Research

• As yet to be defined protocols including proteasome inhibitors and other anti-plasmocyte antibodies with costimulation blockade, B-cell immunomodulation targeting IL-6 as well as cleavage of IgG donor-specific antibodies with imlifidase are highly promising new strategies that deserve further investigation.

## Outcomes After HLA Incompatible Transplantation

Results from HLAi are often compared with those from compatible transplants, but many HLAi patients will never have the option of a compatible transplant, as the chance for the most highly sensitized to receive a deceased donor kidney, or matching in a KEP is essentially nil (48,61). It is important, therefore, when considering outcomes, to also include patients who remain on dialysis and who are waiting for an organ offer as comparators.

We have therefore considered the following:

• A comparison of mortality rates between HLAi and those who remain without a transplant
• A comparison of morbidity between HLAi and those who remain without a transplant
• A comparison of quality of life between HLAi and those who remain without a transplant

### Mortality

There are only four studies comparing mortality in those who have undergone HLAi with those who remain on the waiting list, and these are detailed in Table 2. The study by Montgomery (44) compared outcomes from a single center with those in patients taken from the United Network for Organ Sharing (UNOS) database. There was a clear survival advantage for those who underwent HLAi compared with remaining on the waiting list.

**TABLE 2:** Mortality in HLAi transplant recipients versus those not transplanted and remaining on the waiting list.

	Country	Time (years)	Patient survival, %		*p*-value
			HLAi transplant	No transplant, but on waiting list	
(44)	United States	8	80.6% *n* = 211	30.5% *n* = 1,050	*p* < 0.001^a^
(62)	United States	8	76.5% *n* = 1,025	43.9% *n* = 5,125	*p* < 0.001^a^
(61)	United Kingdom	7	78.3% *n* = 213	76.9% *n* = 852	*p* = NS^b^
(64)	Korea	7	96.3% *n* = 189^c^	88.2% *n* = 930	*p* < 0.001

a Kaplan Meier.

b Kaplan Meier and log rank test.

c Includes cross-match negative recipients.

NS, not significantly different.

However, it might have been possible that the survival benefit shown for HLAi was due to the approach in this (expert) center, so in 2016, a study by Orandi (62) considered HLAi in 1,025 patients from 22 centers in the United States (these included 185 DSA positive, crossmatch negative patients). The results were strikingly similar.

The results from these studies have been partly contradicted by a UK registry study, which found no difference in survival when comparing 213 HLAi patients with 852 well-matched controls who remained on the waiting list (61). It is unclear why findings differ between the United States and Europe, but one explanation may be a generally lower historical survival rate on dialysis in the United States (63).

More recently, a study from Korea compared 131 patients, from two hospitals, with a positive CDC or flow crossmatch (and 44 with DSA but a negative crossmatch) with a group of matched controls of those who were waiting for a transplant (*n* = 3,701), or who received a deceased donor transplant (*n* = 907). They found that patient survival was significantly better for those undergoing an HLAi transplant, compared with either control group (64).

It remains unclear whether there is a survival advantage from an HLAi transplant, compared with remaining on the waiting list; nevertheless, no survival disadvantage for HLAi was found.

### Morbidity

There are no studies that compare morbidity in those undergoing HLAi with those who remain on the waiting list. This is an important gap in our knowledge, particularly given the statements above regarding survival. There is one study by Orandi (65), which compared hospital readmissions in 379 HLAi transplants with matched controls who remained on the waiting list, using registry data from the United States. Those who underwent HLAi, unsurprisingly, had a higher readmission rate in the first month (RR 5.86; 95% CI 4.96–6.92; *p* < 0.001), but interestingly, had lower rates of hospitalization subsequently (at 3 years: RR 0.74; 95% CI 0.66–0.84; *p* < 0.001).

A report by Kim (66) compared 56 HLAi (positive T cell flow cytometric crossmatches were excluded) with 274 compatible transplants, providing data on infectious complications, which may help in considering the risk. Urinary tract infections (41% vs. 7.7%), cytomegalovirus viraemia (54% vs. 14%) and pneumocystis jiroveci pneumonia (PJP) (5% vs. 0%) were all significantly higher in the HLAi group (*p* < 0.001). Another study that compared 27 HLAi patients with 69 ABOi patients, found no significant difference in viral, bacterial or fungal infections between the two groups, although of note, 6% of the ABOi group had PJP, compared with none of the HLAi group (67).

### Quality of Life

We were unable to find any studies that compared quality of life in those undergoing HLAi, with those remaining on the waiting list and hoping for a compatible transplant.

### Recommendations

#### Areas for Further Research

• We recommend that data be collected prospectively for sensitized patients, in order to compare the effect of an HLA incompatible transplant with remaining on the waiting list. This data should include mortality, morbidity and quality of life.

## Strategies for Access to Kidney Transplantation for Highly Sensitized Patients

Some patients have cellular memory without current circulating antibodies detectable in the peripheral blood. There are currently no clinically validated and available tools that accurately assess such cellular memory responses. It is therefore difficult to propose well-substantiated recommendations for this type of risk.

Among the most successful transplant policies are 1) sliding scales or priority points programs; 2) an allocation system based on AM HLA antigens rather than the avoidance of unacceptable ones and 3) achieving HLA compatibility using living donor transplant options, such as ABO incompatible transplantation or KEP.

HLA immune responses are driven both by alloreactive T and B lymphocytes. However, while alloreactive T cells are key in allograft rejection, there is a lack of sensitive and validated immune tools that can be implemented clinically to mitigate these effects (68,69). Currently, immune-risk stratification in kidney transplant candidates is focused on the humoral effector pathway through the detection of serum anti-HLA antibodies directed against donor antigens, but interpretation of SAB data may be affected by antibody titer, prozone effect, or competition of shared epitopes on different beads, as well as irrelevant antibody reactivity against denatured HLA molecules (70–72). The ability of DSA identified by SAB to bind donor cells *ex vivo* in FCM is a good predictor of subsequent ABMR lesions and graft loss in 50% and 30% of recipients, respectively (73–76). Importantly, by accepting every SAB signal, a high number of patients would be defined as highly sensitized, with the consequently lower chance of receiving an organ offer through regular allocation systems—likely reducing a patient’s chance by up to five-fold (76). Therefore, an individualized risk-assessment of previous sensitizing events, adding a thorough epitope analysis and most importantly, the likelihood of receiving an HLA compatible transplant in their respective region, should be considered.

**FIGURE 2:**
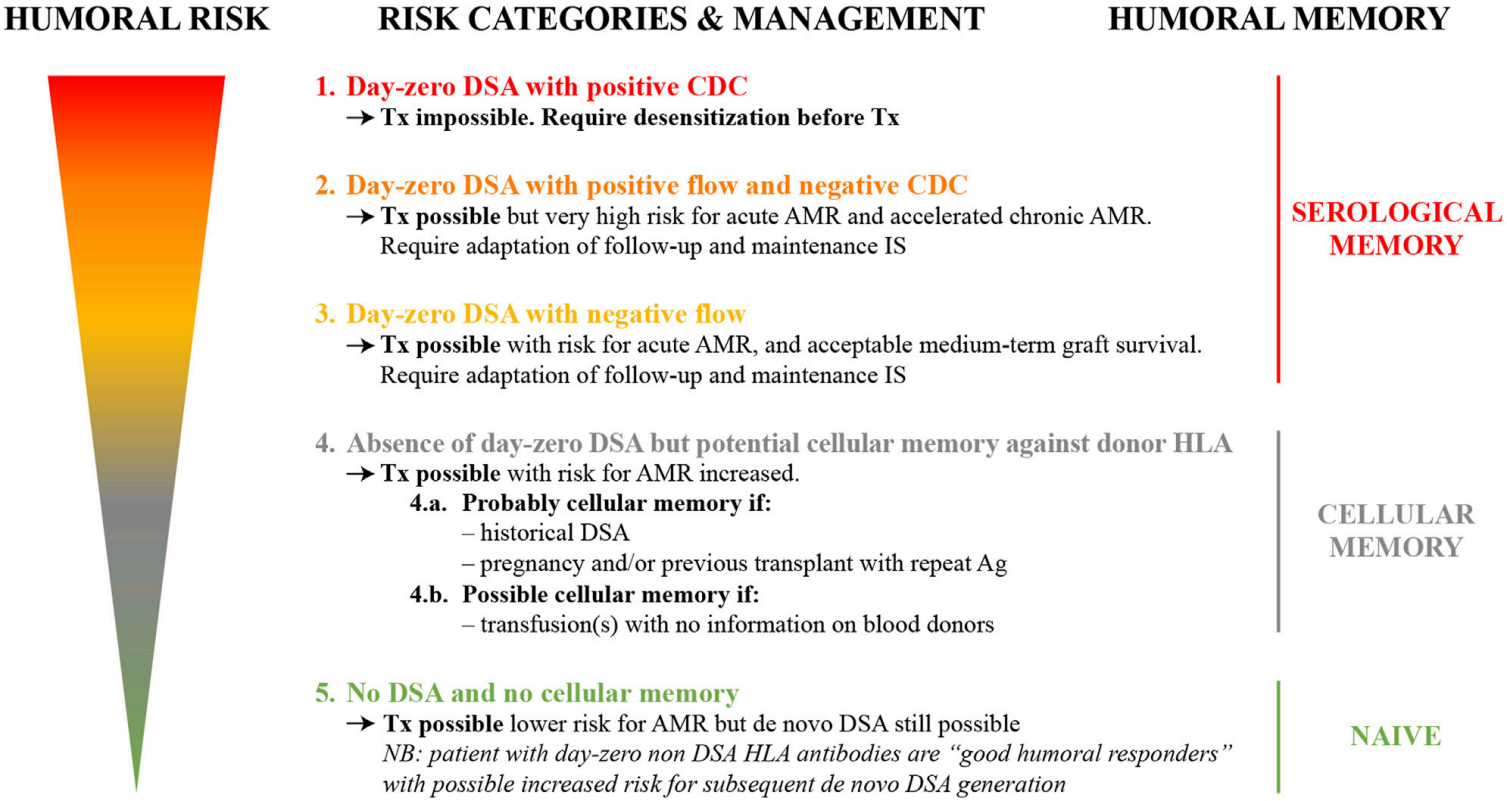
Humoral risk stratification of kidney transplant candidates (adapted from reference (1)) AMR, antibody-mediated rejection; CDC, complement-dependent cytotoxicity; DSA, donor-specific antibodies; HLA, human leukocyte antigen; IS, immunosuppression; Tx, transplant.

A European working group endorsed by the ESOT, ENGAGE, has put forward an initiative proposing an integrative consensus of the most consistent evidence to stratify kidney transplant candidates into five distinct risk categories with the aim of conferring the best chance of successful transplantation. These risk categories take into account an individual patient’s past immunological clinical background, integrated with an assessment of serological alloimmune memory using CDC-crossmatch, FCM crossmatch and SAB assays (1) (Figure 2).

**FIGURE 3:**
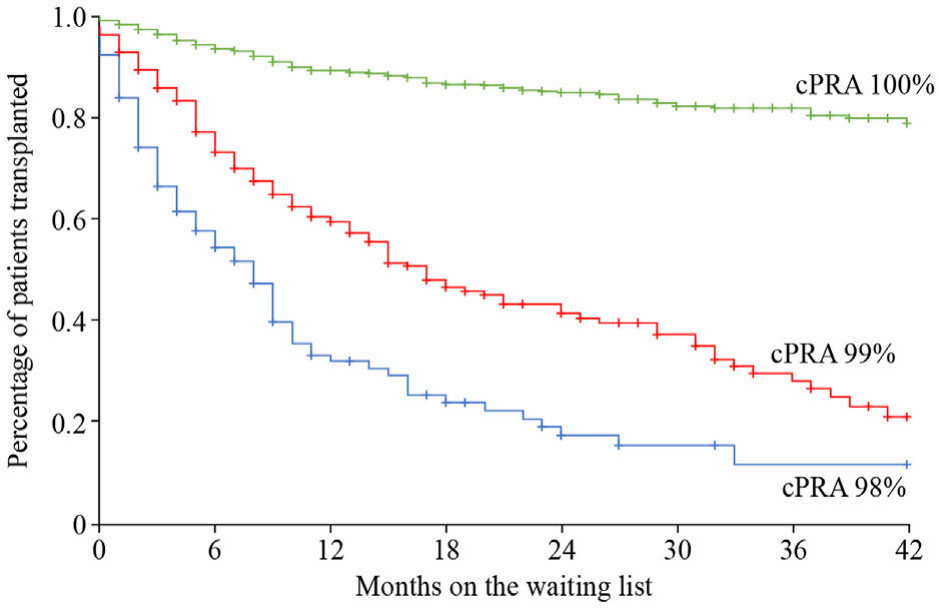
Time on the wait list and percentage of patients receiving a kidney transplant relative to patient cPRA in the priority program for highly sensitized kidney transplant patients in Spain. Image reproduced with thanks and with permission from the Spanish priority allocation programme (PATHI) from the Spanish National Transplant Organization (www-ONT.es). cPRA, calculated percentage of actual organ donors who express one or more unacceptable antigens.

The use of a sliding scale priority points system for allocation of deceased donor organs can increase the transplant rate for highly sensitized transplant candidates. In the United States, those with a cPRA ≥98% receive a higher sliding scale priority point score, in which ABO incompatible (A2/A2B to B organ) offers are also permitted due to their lower immunogenicity (77–79). Remarkably, kidney transplant rates among these patients dramatically increased when the scale was introduced, from 2.5% to 13.4% (80). A similar scheme exists in Spain, with a national sliding scale priority program using an ABO identical deceased organ donor allocation system (PATHI) (81). However, these programs have only significantly helped access to transplantation for those transplant candidates with a cPRA<100% (80,82,83). For those with 100% cPRA, sliding priority points schemes do not seem to increase their chance of receiving a kidney transplant, or even an organ offer, especially when stratifying the levels of sensitization into decimals (99.95%–100%) (84) (Figure 3).

KEP are discussed earlier but some important points with respect to risk stratification are:

• National demographics: the incidence of blood groups and HLA types varies across different countries, and will therefore affect the chances within a KEP
• The size of the pool: the larger the pool the greater the chances of a match, although there is probably a maximum size beyond which there is no incremental advantage
• Recipient characteristics: for example, those who are very highly sensitized (e.g., cPRA/cRF 100%) will have a low or even negligible chance in a KEP, for the same reasons that they will have a low chance of receiving a deceased donor transplant
• KEP algorithm: each KEP will have its own algorithm, which will affect the chances an individual has for a match in the scheme. This should be considered when entering a patient into the scheme.

The easiest way to address these factors is to access an online calculator which incorporates the factors into a probability of a match, ideally with confidence intervals. An example from the UK scheme is given at https://www.odt.nhs.uk/living-donation/tools-and-resources and at https://www.odt.nhs.uk/transplantation/tools-policies-and-guidance/calculators/ , which addresses the likelihood of a deceased donor transplant for sensitized patients.

**FIGURE 4:**
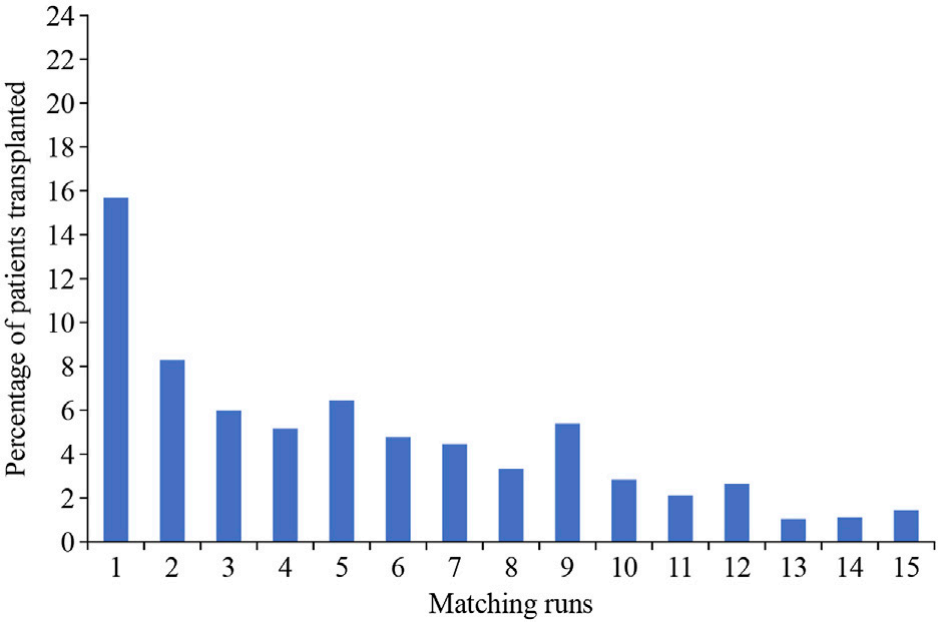
Correlation of the chance of a transplant relative to the number of matching runs (UK figures from National Health Service Organ Donation and Transplantation Clinical website: https://www.odt.nhs.uk).

Finally, an important point to consider is that entry into a KEP should not be considered as a definitive solution. Figures from the UK KEP show that the incremental chance of a match after 6 or 7 “runs” is low (Figure 4), and thus, at this stage, if there are alternatives, such as a direct antibody incompatible transplant, these should be considered.

**FIGURE 5:**
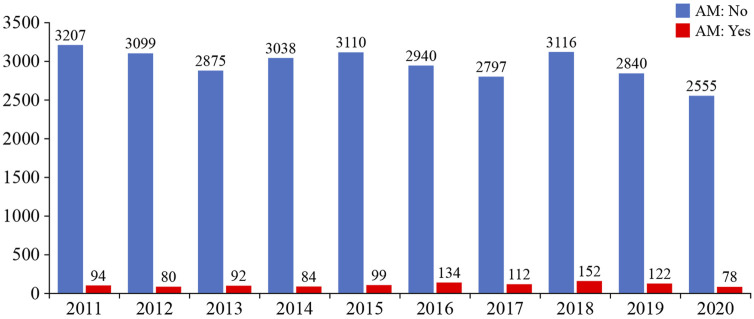
Relative numbers of kidney transplantations achieved by Eurotransplant and by the Acceptable Mismatch (AM) program (image reproduced with permission from Eurotransplant, www.eurotransplant.org. https://statistics.eurotransplant.org; accessed May 2021).

The Eurotransplant AM program fully prioritizes the allocation of compatible donor kidneys to highly sensitized patients (>85% cPRA), focusing on finding acceptable matches rather than to prohibit matches (29). The main advantage of the AM over prioritization schemes is that it entails better matching and thus may lead to better long-term outcomes. Unfortunately, it does not seem to increase access to transplantation for those very highly sensitized patients (>99% cPRA). Nevertheless, a considerable number of patients have already been transplanted within the AM program, both first and repeat transplantations (Figure 5). Interestingly, kidney transplant failure is significantly lower in the highly sensitized patients included in the AM program, compared with highly sensitized patients not included in the AM program. Furthermore, death-censored graft survival rate is similar to the rate in non-sensitized patients and is related to a lower chance of rejection in the highly sensitized patients included in the AM program (85).

### Recommendations

• To define the humoral risk in kidney transplantation, the use of the ENGAGE 5 strata system is recommended (1C).
• Prioritization policies should be linked across countries for equity of access (1C).
• The Eurotransplant Acceptable Mismatch program should be expanded to other European countries (that do not have this type of matching) to improve donor/recipient matching (1C).
• All kidney exchange programs should develop calculators to help assess the probability of an organ match (1C).
• Therapeutic options (including HLA- or ABO- incompatible transplantation) should be reconsidered if there are no organ offers for a patient in a kidney exchange program (1C).

#### Areas for Further Research

• Work to develop schemes to help patients with very high cPRA or cRF who may not be transplanted in kidney paired donations or under deceased donor priority schemes should continue.
• The role of induction immunosuppression in relation to sensitization and its role in long-term outcomes should be further explored.
• Whether better risk stratification, thorough immunological evaluation and avoidance of HLA-DSA can improve outcomes should be determined.

## An Integrated Approach Towards Sensitized Patients

**FIGURE 6:**
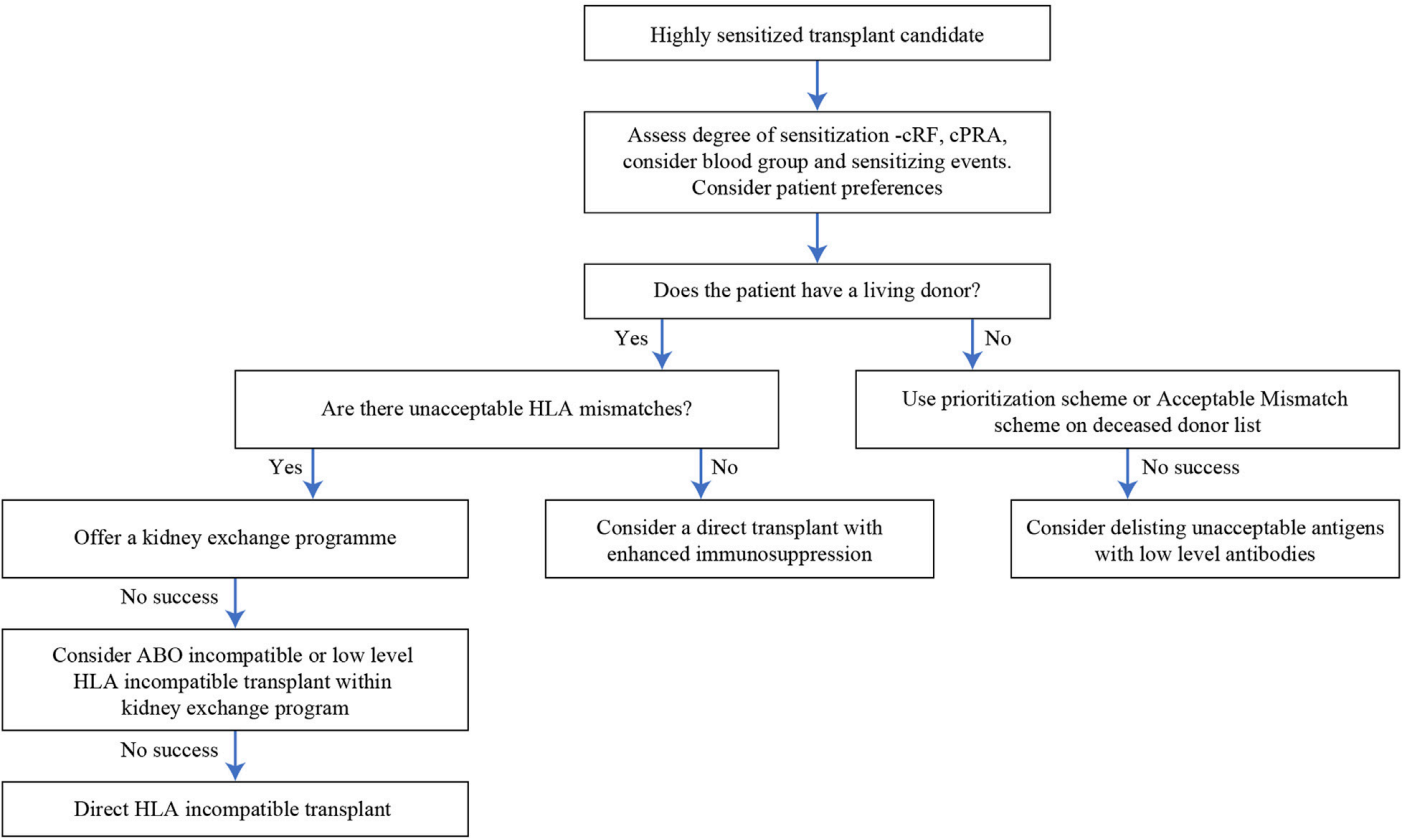
Algorithm of options for a highly sensitized transplant candidate.

We have given a suggested algorithm for approaching patients with HLA antibodies in Figure 6, since we believe that the options described above are not necessarily independent of each other but can be integrated in a clinical decision. This will not be applicable in all settings, since it will depend on the availability of the various modalities, but we hope it will prove to be a useful framework. Two points are worth emphasizing—firstly, that for individual patients, the risks of the various options (including no transplant) should be assessed and conveyed using the limited data that is available. Secondly, flexibility is important; a patient should not be left in a KSS indefinitely if other options are available, or if new treatments appear.
