# Supplementary material for: European Guideline for the Management of Kidney Transplant Patients With HLA Antibodies: By the European Society for Organ Transplantation Working Group
Source: Transpl Int. 2022 Aug 10;35:10511. doi: 10.3389/ti.2022.10511 (PMC9399356; doi:10.3389/ti.2022.10511)
Supplement: Supplementary file 1 [file DataSheet1.pdf]

ESOT

Leading the way in transplantation

European Guidelines for the Management of Kidney Transplant Patients with HLA antibodies

BY THE EUROPEAN SOCIETY FOR ORGAN TRANSPLANTATION (ESOT) WORKING GROUP

esot.org

European Guidelines for the Management of Kidney Transplant Patients with HLA antibodies: By the European Society for Organ Transplantation (ESOT) Working Group

Nizam Mamode,[1] Oriol Bestard,[2] Frans Claas,[3] Lucrezia Furian,[4] Siân Griffin,[5] Christophe Legendre,[6] Liset Pengel,[7] Maarten Naesens.[8]

[1]Department of Transplant Surgery, Guys Hospital, London, UK;
[2]Department of Nephrology and Kidney Transplantation, Vall d'Hebrón University Hospital, Barcelona, Spain;
[3]Department of Immunology, Leiden University Medical Center, Leiden, The Netherlands and University of Antwerp, LEMS, Antwerp, Belgium;
[4]Kidney and Pancreas Transplantation Unit, Department of Surgical Gastroenterological and Oncological Sciences, University Hospital of Padua, Padua, Italy;
[5]Department of Nephrology, University Hospital of Wales, Cardiff, UK;
[6]Department of Nephrology and Adult Kidney Transplantation, Hôpital Necker and Université de Paris, Paris, France;
[7]Centre for Evidence in Transplantation, University of Oxford, UK;
[8]Department of Microbiology, Immunology and Transplantation, KU Leuven, Leuven, Belgium;

Funding
The working group was supported by an unrestricted grant from Hansa Biopharma AB, Lund Sweden.

Acknowledgments
Support of the working group is gratefully acknowledged from ESOT: Giovanna Rossi, Ariane Brusselmans, Chloe Xilinas and Devi Mey.
Thanks to Fiona Loud (Kidney Care UK) for input and patient perspective on discussions.
Medical writing support was provided by Linda Edmondson and Rebecca Mant, independent medical writers, funded by the European Society of Organ Transplantation (ESOT).
This work was previously presented as part of the review process at the European Society of Organ Transplantation Workstream 06 Expert Working Group meeting on 28th August 2021 in Milan, Italy. Full list of delegates at this meeting is included in Appendix 2.

European **S**ociety for **O**rgan **T**ransplantation (ESOT) and the **T**ransplantation **L**earning **J**ourney (TLJ) project

Workstreams within the TLJ project help to achieve the primary aim of ESOT – to improve patient access to (and outcomes in) transplantation. TLJ workstreams facilitate objective discussion of scientific and clinical research, and expert opinion, to ensure that all perspectives on a topic are considered, with clinically relevant end goals in mind.

ESOT seeks to progress transplantation research, practice and education, and to collaborate with other international bodies, to ensure that policies and regulations are globally consistent and relevant, and based on strong scientific, ethical and clinical foundations.

ESOTLJ2.0
TRANSPLANTATION LEARNING JOURNEY

ESOT
Leading the way
in transplantation

Table of Contents

Introduction……………………………………………………………………….…………5

Chapter 1: Definition of Sensitization………..…………………………………..……10

Chapter 2: Comparison of Practices Across Europe for Dealing with Highly Sensitized Transplant Candidates ……………………………………………………….……......21

Chapter 3: Strategies for Access to Kidney Transplantation in Highly Sensitized Patients..……………………………………………………………………………….35

Chapter 4: Desensitization Strategies in Kidney Transplantation…..…………………..54

Chapter 5: Outcomes after HLA-incompatible Transplantation……………..……..…66

Chapter 6: The Place of Kidney Sharing Schemes for Sensitized Patients…………….71

Appendix 1: Bibliographic Searches……..…………………………………..……...86

Appendix 2: Participants in the Expert Working Group Meeting discussion of the recommendations, Milan, Italy and live-streamed, 28th August 2021……………….…92

Introduction

Nizam Mamode*, on behalf of Workstream 06 of the Transplantation Learning Journey of the European Society of Organ Transplantation
[1]*Department of Transplant Surgery, Guys Hospital, London, UK*

***Correspondence:** *Nizam Mamode, nizam.mamode@gstt.nhs.uk*

Keywords: kidney transplantation, HLA incompatible transplantation, recommendations

Word count (body text only): 1112
Number of figures: 2
Number of tables: 0

1 Background

Kidney transplantation offers patients with kidney failure an opportunity for a longer life and a chance of freedom from renal dialysis. Increasingly, however, highly sensitized patients are remaining on the transplant waiting list for a suitable organ. The screening and practice of transplantation of highly sensitized patients has evolved in tandem with increases in sensitivity of HLA antigen testing, helping to improve the matching of patients with donor organs. The chapters in this series explore the current state of knowledge around this issue and how innovation, immune-system manipulation, patient prioritization schemes and 'thinking outside the box' is increasing the likelihood that highly sensitized patients might safely obtain a transplant.

This working group, composed of leading transplant healthcare professionals from around Europe has undertaken a review of the literature in each of six key areas:

- Definition of sensitization
- Comparison of practices across Europe
- Strategies for access to kidney transplantation for highly sensitized patients
- Desensitization strategies
- Outcomes after HLA incompatible transplantation
- The place of kidney sharing schemes for sensitized patients

A standard systematic search strategy was predefined, using the PICO model to formulate clinical questions. Bibliographic searches were developed for each of the clinical questions by experienced staff from the Centre for Evidence in Transplantation, University of Oxford, UK. Systematic searches were conducted in the Transplant Library (www.transplantlibrary.com), Medline and Embase and consisted of a mixture of free text and controlled vocabulary terms. Full details of the search strategies including search dates can be found in the Appendix.

A clinical member of the work group (or a team of clinical members) then assessed the search results and wrote each chapter in this Supplement. The full development and review process is outlined in **Figure 1.**

An algorithm for patients who are highly sensitized was developed (**Figure 2**), and a series of recommendations:

2 Risk Stratification

- A parameter, which is based on the HLA frequencies of the actual organ donor population, such as cPRA or cRF, should be used to estimate the chance that a sensitized patient can be transplanted with a compatible donor without the need for any special treatment (Chapter 1, Definition of sensitization)
- Further standardization of solid phase assays is recommended (Chapter 1, Definition of sensitization)
- When defining unacceptable mismatches in highly sensitized patients on the basis of (weak) antibody reactivities in SAB assays only, one should consider the not well-defined risk of antibody-mediated rejection in the light of a prolonged waiting time and associated mortality and morbidity (Chapter 1, Definition of sensitization)
- To define the humoral risk in kidney transplantation, the use of the ENGAGE 5 strata system (1) is recommended (Chapter 3, Strategies for access to kidney transplantation for highly sensitized patients)

3 Organ Allocation

- We recommend all countries and centers have an active policy of prioritizing highly sensitized patients for organ transplantation (Chapter 4, Desensitization strategies)
- Access to the donor pool should be increased through greater use of:
  - Sliding scale priority score schemes based on cPRA values (Chapters 1 (Definition of sensitization) and 3, Strategies for access to kidney transplantation for highly sensitized patients)
  - Prioritization policies linked across countries for equity of access (Chapter 3, Strategies for access to kidney transplantation for highly sensitized patients)
  - Increased access to and harmonization of Kidney Exchange Programs, with greater and standardized sharing of outcomes (Chapters 2, Comparison of practices across Europe and 6, The place of kidney sharing schemes for sensitized patients)
  - Inclusion of unspecified kidney donations (if these are performed) in kidney sharing schemes (Chapters 2, Comparison of practices across Europe and 6, The place of kidney sharing schemes for sensitized patients)
  - Inclusion of compatible pairs and deceased donor organs in kidney sharing schemes (Chapter 6, The place of kidney sharing schemes for sensitized patients)
- The Eurotransplant Acceptable Mismatch program should be expanded to other European countries to improve donor/recipient matching (Chapter 3, Strategies for access to kidney transplantation for highly sensitized patients)

- Kidney Paired Donation is the preferred initial option over desensitization given the better transplant outcomes and cost-effectiveness, in both ABO and HLA incompatible pairs, unless there is a need for desensitization, there is clinical urgency or a low chance of a transplant (Chapter 6, The place of kidney sharing schemes for sensitized patients)
- All kidney sharing schemes should develop calculators to help assess the probability of an organ match (Chapter 3, Strategies for access to kidney transplantation for highly sensitized patients)
- Therapeutic options should be reconsidered if there are no organ offers for a patient in a kidney sharing scheme (Chapter 3, Strategies for access to kidney transplantation for highly sensitized patients)

4 Desensitization

- The most efficacious desensitization strategy is to start with rounds of plasma exchanges/immunoadsorption together with B-cell immunomodulation with IVIG or B-cell depletion with anti-CD20 monoclonal antibodies (Chapter 4, Desensitization strategies)
- As yet to be defined protocols including proteasome inhibitors and other anti-plasmocyte antibodies with costimulation blockade, B-cell immunomodulation targeting IL-6 as well as cleavage of IgG donor-specific antibodies with imlifidase are highly promising new strategies that deserve further investigation (Chapter 4, Desensitization strategies)

5 Areas for Further Research

- We recommend that data be collected prospectively for sensitized patients, in order to compare the effect of an HLA incompatible transplant with remaining on the waiting list. This data should include:
  - Mortality
  - Morbidity
  - Quality of Life (Chapters 2, Comparison of practices across Europe and 5, Outcomes after HLA incompatible transplantation)
- Work to develop schemes to help patients with very high cPRA who may not be transplanted in kidney paired donations or under deceased donor priority schemes should continue (Chapter 3, Strategies for access to kidney transplantation for highly sensitized patients)
- A further need for evidence-based information is in the role of induction immunosuppression in relation to sensitization and its role in long-term graft and patient outcomes (Chapter 3, Strategies for access to kidney transplantation for highly sensitized patients)
- Better risk stratification, thorough immunological evaluation and avoidance of HLA-DSA should be used to improve outcomes after kidney transplantation (Chapter 3, Strategies for access to kidney transplantation for highly sensitized patients)
- Better HLA matching and a restricted transfusion policy will probably diminish the number of highly sensitized patients, but more data are needed in this area (Chapter 1, Definition of sensitization)

6 Postscript

During the Expert Working Group meeting on 28th August 2021 to discuss these guidelines and the research supporting them, the particular issues facing children needing kidney transplant surgery early in life was raised. Although outside the initial scope of this group, the issues are relevant, and deserving of further time and research.

Abbreviations

cPRA, the calculated percentage of actual organ donors who express one or more unacceptable antigens; cRF, calculated reaction frequency; DSA, donor-specific antibodies; ENGAGE, EuropeaN Guidelines for the mAnagement of Graft rEcipients (ENGAGE) working group; HLA, human leukocyte antigen; SAB, single antigen bead; PICO, population, intervention, control, outcomes format for framing a research question.

Author Contributions

This chapter was written by Nizam Mamode. All members of WS06 of ESOT provided input and critical review of this chapter: Oriol Bestard, Frans Claas, Lucrezia Furian, Siân Griffin, Christophe Legendre, Maarten Naesens, Liset Pengel.

Conflict of Interest

The author declares that the research was conducted in the absence of any commercial or financial relationships that could be construed as a potential conflict of interest. The author has received honoraria from Hansa, Chiesi, Novartis and Takeda.

7 Reference

1. Bestard O, Couzi L, Crespo M, Kessaris N, Thaunat O. Stratifying the humoral risk of candidates to a solid organ transplantation: a proposal of the ENGAGE working group. Transpl Int (2021) 34:1005-18. doi: 10.1111/tri.13874

FIGURE 1. Schematic of preparation of chapters and recommendations

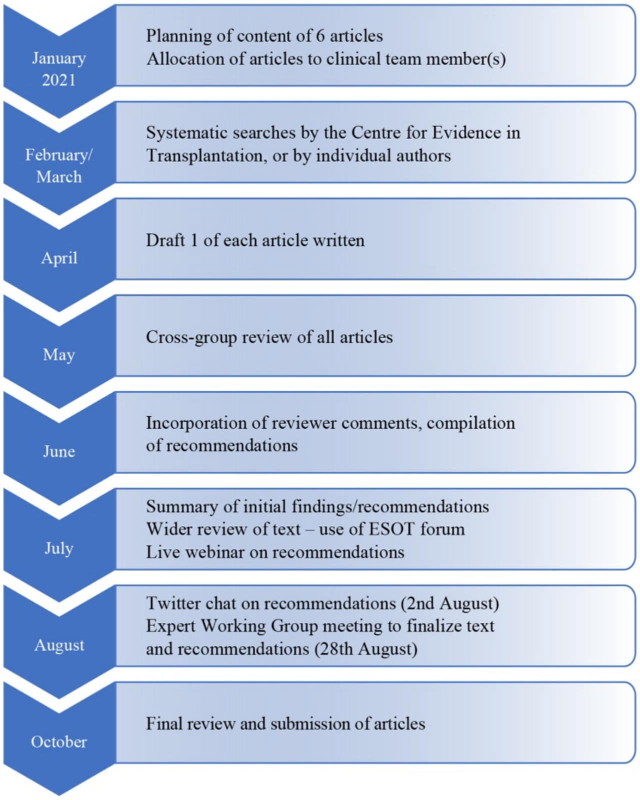

FIGURE 2. Algorithm of options for a highly sensitized transplant candidate

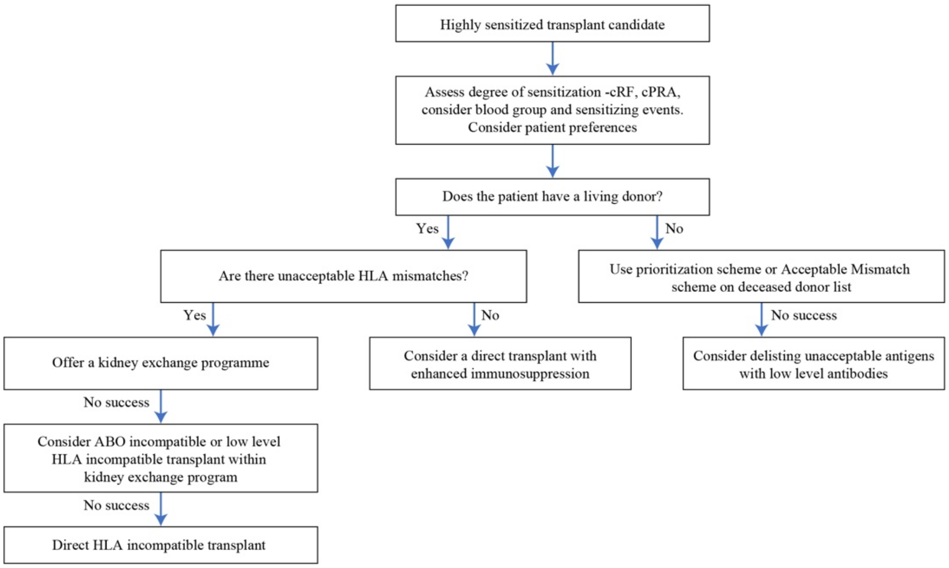

Chapter 1: Definition of Sensitization

Frans Claas*, on behalf of Workstream 06 of the Transplantation Learning Journey of the European Society of Organ Transplantation
Department of Immunology, Leiden University Medical Center, Leiden, The Netherlands and University of Antwerp, LEMS, Antwerp, Belgium

***Correspondence:** Frans Claas, f.h.j.claas@lumc.nl*

Keywords*:* sensitization, donor specific antibodies (DSA), renal transplantation, rejection, risk assessment

Word count (body text): 2365
Number of figures: 1
Number of tables: 0

Abstract
Finding a transplant match for a patient has evolved from defining sensitization based on the reactivity of a patient's serum in complement-dependent cytotoxicity (CDC) and the percentage of positive panel donors in an antibody screening assay (%PRA) to the use of virtual PRA (vPRA), calculated PRA (cPRA) or calculated reaction frequency (cRF). These methods are based on the HLA antigens recognized by the antibodies present in the serum of the patient in relation to the HLA phenotypes of the actual organ donor population. However, more sensitive solid phase assays have complicated the definition of a (highly) sensitized patient as the presence of specific antibodies in CDC was always considered a contra-indication for transplantation due to hyper-acute rejection, but HLA antibodies detectable in solid phase assays are considered risk factors, but not necessarily contra-indications. The challenge is to define which HLA antigens recognized by the antibodies in the serum of a patient should be unacceptable mismatches for a potential organ donor. Although different strategies have been developed to enhance transplantation of highly sensitized patients, the best option for the patient would be prevention. Novel molecular HLA matching strategies are likely to decrease the number of highly sensitized retransplant candidates.

1 Introduction

In the early days of renal transplantation, it became evident that the presence of donor-specific HLA antibodies (DSA) before transplantation was associated with a high incidence of hyper-acute rejection (1, 2). Such HLA antibodies can be induced by previous blood transfusions, pregnancies or transplants (3–5). The incidence of hyper-acute rejection was significantly diminished by the introduction of serological crossmatching and the exclusion of donors toward which the potential recipient had circulating HLA antibodies (2). However, this strategy had an enormous impact on the transplantation rate of highly sensitized patients. Due to their broad sensitization, these patients had positive crossmatches with virtually all potential donors and accumulated on transplant waiting lists. Without special strategies, many of these patients would be

unlikely to ever receive a suitable transplant and would have a high chance of dying while on the waiting list.
The introduction of sensitive laboratory techniques that detect the presence and specificity of HLA antibodies, and their impact on clinical outcome has led to much discussion on the definition of a sensitized and a highly sensitized patient, which is the topic of this chapter.

2 Historical Definitions

Historically, complement-dependent cytotoxicity (CDC) was the gold standard and the only assay available for the detection of HLA antibodies. Patients' sera were screened regularly against a panel of HLA typed blood donors and the degree of sensitization was expressed as a percentage of panel reactive antibodies (%PRA). This %PRA was defined by the percentage of panel donors reactive with the patient serum in CDC. As the composition of the panels varied enormously between laboratories, the same held true for the %PRA reported. Furthermore, the %PRA was based on the composition of a panel consisting of voluntary blood donors, which does not necessarily reflect the frequency of the HLA antigens in the actual organ donor population. The %PRA was often reported irrespective of the specificity of the antibodies, which made this parameter an inaccurate predictor of the chance that the patient would be confronted with a positive crossmatch as other antibodies, as well as HLA antibodies, are able to cause a positive CDC reaction. The definition of a highly sensitized patient also varied, but often a PRA>85% was considered the threshold for a highly sensitized patient (6).
Apart from the %PRA, an extensive analysis of the reaction patterns of the potential transplant patient to the HLA types of the panel donors could lead to the identification of specific antibodies, provided that the sensitization was not too broad. When an antibody reactive with a specific HLA antigen was identified i.e. anti-HLA-A2, this antigen was considered to be unacceptable and all HLA-A2-positive organ donors were excluded for this patient.
CDC crossmatch only detects those HLA antibodies that are able to activate complement i.e. IgM and the IgG subtypes IgG1 and IgG3. In order to also detect the non-complement fixing IgG subclasses IgG2 and IgG4, Flow Cytometric crossmatch (FCM) was introduced in several laboratories (7, 8). Donor-specific antibodies (DSA) detectable in FCM, but not in CDC, appeared to be clinically relevant and were associated with graft rejection and graft loss in a proportion of recipients (9). In contrast to CDC reactive DSAs, antibodies detected in FCM were considered more as a risk factor than a contra-indication for transplantation.
Both CDC and FCM are cell-based assays and a positive reaction in these assays does not necessarily mean that the target of the antibody is an HLA antigen. Clinically irrelevant antibodies reactive with other structures on lymphocytes can interfere in the outcome of both a CDC and an FCM crossmatch (10, 11) leading to false positive crossmatches. These irrelevant antibodies also include auto-antibodies, which react with the patients' own lymphocytes. In addition, the endothelial cells in the kidney can express alloantigens, which are not present on lymphocytes (12) and reliable assays to detect antibodies reactive with endothelial cell-specific antigens are currently lacking.

3 Impact of the Introduction of Solid Phase Assays

Solid phase assays were introduced to prevent non-HLA antibodies interfering in the establishment of HLA sensitization (13). Targets for antibody detection in these assays are isolated HLA molecules fixed to a solid surface. Any antibody reactivity detected in this assay is by definition directed against an HLA antigen. The introduction of single antigen beads (SAB) has facilitated the detection and, especially, the identification of specific HLA antibodies (although the results are not always straightforward (14, 15)). Patient serum is tested against a mix of about one hundred (and recently more) different beads, each individual bead covered with HLA molecules of the same specificity. The degree of antibody binding to a specific bead is expressed as mean fluorescence intensity (MFI). This assay appears to be far more sensitive than CDC and FCM for detecting HLA antibodies and DSA. As a consequence, the proportion of sensitized patients has significantly increased after the introduction of solid phase assays (16).

The clinical relevance of antibodies detectable in SAB assays is still a matter of debate (17). Individual centers have tried to make correlations between the already established clinical relevance of CDC and FCM and the MFI values obtained in SAB (i.e.(18)).

Although no absolute thresholds can be defined, it is generally accepted that the highest MFI values predict a positive CDC crossmatch, although exceptions exist as some high MFIs are associated with a negative CDC. Moderate and high values are thought to be associated with a positive FCM. The risks associated with the presence of DSA with these MFIs are presumed to be similar to the ones already established for a positive CDC or FCM (Figure 1)(19). As the SAB assay is very sensitive, positive reactions are obtained, usually with a lower MFI, which do not correlate with a positive FCM or CDC crossmatch. The clinical value of such antibodies has been extensively studied with some conflicting results (20, 21). Overall, there seems to be a suggestion of increased risk of early antibody-mediated rejection in DSA-positive transplantation, which may be related to the MFI. The impact of these increased rejection rates on graft function and survival are less certain (Supplemental Data 1).

There are several technical issues related to SAB assays. For MFI, the parameter used to indicate the strength of the antibody reactivity is just a semi-quantitative marker (22, 23) and for that reason it is virtually impossible to define an exact positive or negative reaction. Most centers use a cut-off of 1000–1500 (19) but there is no general agreement on this value. Also, the fact that two vendors provide kits with different sensitivities makes a general definition of a positive reaction impossible (24). Among other things, the MFI is affected by the affinity and avidity of the antibodies but also by the number of different beads reactive with the antibody. HLA antibodies are directed against specific epitopes expressed on the target HLA antigen, but individual epitopes can be shared by (many) different HLA alleles (25). If an antibody is directed against an epitope only expressed on one allele, its MFI will be higher than that of an antibody with exactly the same characteristic but reactive with 30 different HLA alleles as these will compete for antibody binding. Another complication is the fact that not all antibodies reactive in SAB are directed against intact HLA molecules. Studies in non-immunized males showed that their sera contained antibodies reactive with denatured HLA antigens attached to the beads leading to a positive reaction (26). Patients with DSA directed against denatured HLA appeared to have a similar rejection incidence and graft survival as non-immunized

patients (27). Therefore, it is important to link positive reactions in SAB to known sensitizing events such as pregnancies, blood transfusions, previous transplants before considering an antibody clinically relevant and a target antigen unacceptable on a future organ donor.

4 cPRA, vPRA, cRF, Novel Parameters to Define the Degree of Sensitization

The historical parameter for indicating the degree of sensitization is the %PRA, but this parameter is not very accurate as the specificity of the antibodies causing the positive reactions is often unknown and clinically irrelevant antibodies could contribute to the %PRA.

The introduction of solid phase assays has improved the possibility of determining the HLA specificities of the antigens recognized by the antibodies present in the patient's sera. The specificities recognized in solid phase assays are also instrumental for clarifying the specificity of the antibody patterns observed in CDC and FCM. This has led to a solid basis for the introduction of more reliable parameters for the definition of the degree of sensitization based on the antibody specificities present in the patient and the HLA phenotypes of the actual organ donor population. Different names are now circulating for this novel parameter: vPRA (virtual PRA) (28), cPRA (calculated PRA) (29) and cRF (calculated reaction frequency) (30) but they all reflect the chance that a patient has HLA antibodies reactive with a donor derived from the actual organ donor population.

The definition of highly sensitized patients and making them eligible for prioritization in organ allocation is variable between transplant centers and between countries as became clear from a recent informal survey by the European Society of Transplantation (ESOT). Each transplant center responding to the survey had their own threshold for calling a patient highly sensitized, and also for including a patient in a special program for organ allocation. Several recent studies have shown that, especially in patients with a vPRA, cPRA or cRF >98%, there are difficulties in finding a suitable donor without the help of a special program or treatment (31–33).

5 Risk Estimation in Sensitized Patients

As mentioned, serological crossmatching and HLA antibody screening have been introduced to prevent the occurrence of hyper-acute rejection. However, the definition of HLA antibodies and/or unacceptable mismatches has a broader application and is mainly aimed at immune risk assessment (17, 19, 34). Patients with DSA detectable in CDC, FCM and SAB (high MFI) are still at risk for hyper-acute rejection. Patients with DSA in FCM and SAB (medium MFI) but not in CDC are at risk for early antibody-mediated rejection. Patients with DSA only in SAB (lower MFI) are at a lower risk for antibody-mediated rejection and it remains to be established whether further fine-tuning SAB antibody detection will contribute to a better risk assessment. Assays have been developed to measure the complement binding capacity (35, 36), or to identify the IgG subclass of the antibodies reactive in SAB (37) but it is not clear whether these modified assays really contribute to further risk assessment when performed before transplantation (34, 38).

The actual challenge, in the case of SAB reactive antibodies with a low MFI, is to define whether their target antigen should be considered an unacceptable mismatch or not. A

link with a specific sensitization event may help but, especially for highly sensitized patients, one should consider the low risk for a rejection event in the light of the risk of mortality or morbidity due to the fact that the patient will not be transplanted and will remain on dialysis (39, 40).

6 Conclusions

The definition of a sensitized patient has changed enormously since the introduction of solid phase assays. Rather than the reactivity of the patient's serum against a panel of blood donors as the basis for the %PRA, the specificity of the HLA antibodies now plays a pivotal role.

Calculating the chance that specific patient antibodies react with the HLA antigens of the actual organ donor population, has created a more reliable parameter (vPR, cPRA, cRF) for the degree of sensitization. However, the sensitivity of the solid phase assays has also led to complications including the assessment of which antibodies should be considered as clinically relevant and a contra-indication for transplantation. Not every HLA antigen reactive in a solid phase assay should be considered as an unacceptable mismatch.

What has not changed is the fact that highly sensitized patients have a very low chance of being transplanted with a compatible donor organ and that special strategies are required to enable successful transplantation of these patients.

One approach is to try to prevent patients from becoming (highly) sensitized. A recent analysis of the background of highly sensitized patients transplanted via the Eurotransplant acceptable mismatch program showed that more than 70% had been immunized by a previous transplant (41). Better HLA matching of the first transplant and avoiding blood transfusions prevents the induction of these HLA antibodies (42). Although classical HLA antigen or allele matching might help to some extent, novel match strategies, which take advantage of the fact that the amino acid sequence of the different HLA antigens is known, show very promising results. This has led to the identification of those amino acids responsible for the induction and reactivity of HLA antibodies called eplets or epitopes. Every HLA antigen consists of a unique set of epitopes but the individual epitopes can be expressed on different HLA antigens (25). A patient will not make antibodies to epitopes expressed on their own HLA antigens even when these self-epitopes are expressed on a mismatched HLA antigen. As a consequence, the number of foreign epitopes on a single HLA mismatch varies and depends on the patient's HLA type. Many recent studies show a clear beneficial effect of molecular HLA matching of donors and recipients (43–47), (based on epitopes, eplets, amino acids or electrostatic properties (44)), on the induction of de novo DSA (Supplemental Data 2). Inclusion of these novel matching strategies in the allocation of donor kidneys will certainly decrease the number of (highly) sensitized retransplant candidates on the waiting list. This is especially true and will be of benefit for children requiring a transplant, who are very likely to need more than one transplant during their lifetime.

7 Recommendations

Risk Stratification

- A parameter, which is based on the HLA frequencies of the actual organ donor population, such as cPRA or cRF, should be used to estimate the chance that a sensitized patient can be transplanted with a compatible donor without the need for any special treatment
- Further standardization of solid phase assays is recommended
- When defining unacceptable mismatches in highly sensitized patients on the basis of (weak) antibody reactivities in SAB assays only, one should consider the not well-defined risk of antibody-mediated rejection in the light of a prolonged waiting time and associated mortality and morbidity

Organ Allocation

- Increase access to the donor pool, through greater use of:
  - Sliding scale priority score schemes based on cPRA values (Chapters 1 and 3)

Areas for Further Research

- Better HLA matching and a restricted transfusion policy will probably diminish the number of highly sensitized patients, but more data are needed in this area

Abbreviations

CDC, complement-dependent cytotoxicity; cPRA, calculated PRA; cRF, calculated reaction frequency; DSA, donor-specific antibodies; FCM, flow cytometric crossmatch; HLA, human leukocyte antigen; MFI, mean fluorescence intensity; %PRA, the percentage of positive panel donors in an antibody screening assay; SAB, single antigen bead; vPRA, virtual PRA

Author Contributions

The members of WS06 of ESOT provided input and critical review of this chapter: Oriol Bestard, Lucrezia Furian, Siân Griffin, Christophe Legendre, Nizam Mamode, Maarten Naesens, Liset Pengel.

Conflict of Interest

The author declares that the research was conducted in the absence of any commercial or financial relationships that could be construed as a potential conflict of interest.

FIGURE 1. The association between the mean fluorescence intensity of donor specific antibodies detected in single antigen beads and the outcome of complement-dependent cytotoxicity and flow cytometric crossmatch is reflected in the risk estimation

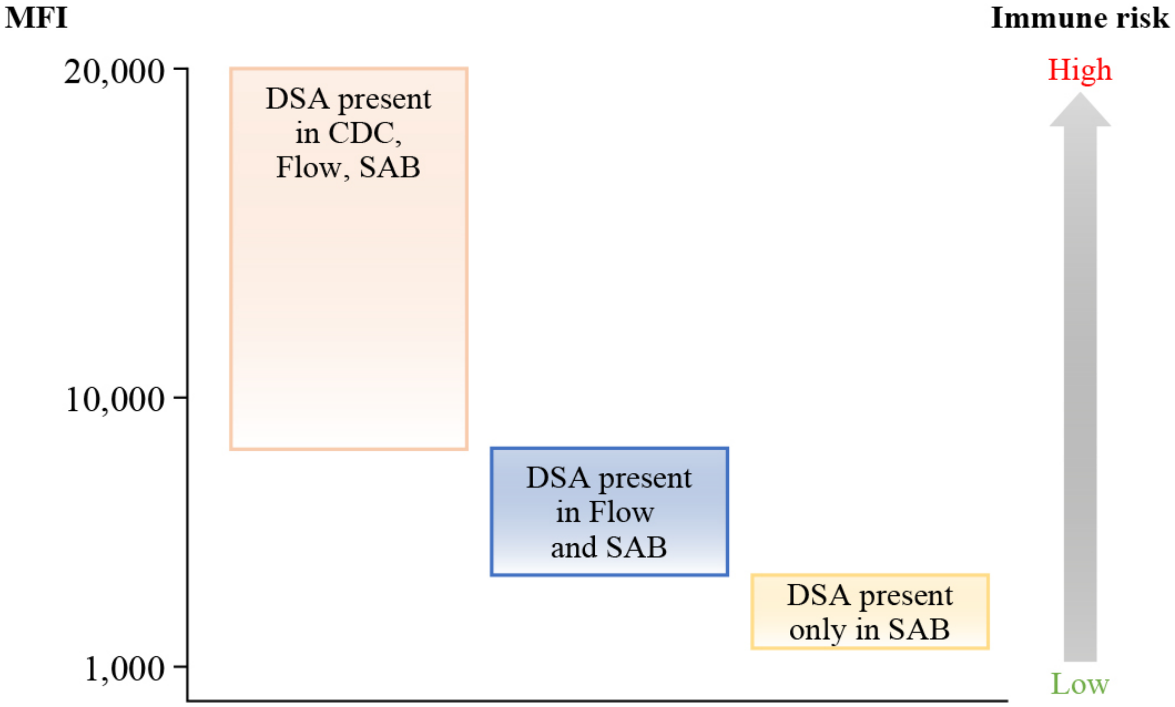

DSA, donor-specific antibodies; Flow, flow cytometric crossmatch; SAB, single antigen beads

References

Supplemental Data 1: Evidence Report: The Sir Peter Morris Centre for Evidence in Transplantation: Clinical significance of pre-transplant donor-specific antibodies (DSA)
Supplemental Data 2: Evidence Report: The Sir Peter Morris Centre for Evidence in Transplantation: Effect of eplet mismatches on development of donor specific antibodies (DSA)

1. Kissmeyer-Nielsen F, Olsen S, Petersen V, Fjeldborg O. Hyperacute rejection of kidney allografts, associated with pre-existing humoral antibodies against donor cells. Lancet (1966) 24(2(7465)):662-5. doi: 10.1016/s0140-6736(66)92829-7.
2. Patel R, Terasaki PI. Significance of the positive crossmatch test in kidney transplantation. N Engl J Med (1969) 280(14):735-9. doi: 10.1056/NEJM196904032801401
3. Dausset J, Nenna A, Brecy H. Leukoagglutinins in chronic idiopathic or symptomatic pancytopenia and in paroxysmal nocturnal hemoglobinuria. Blood (1954) 9:696-720.
4. Van Rood JJ, Eernisse JG, Van Leeuwen A. Leucocyte antibodies in sera from pregnant women. Nature (1958) 181(4625):1735-6. doi: 10.1038/1811735a0.
5. Morris PJ, Williams GM, Hume DM, Mickey MR, Terasaki PI. Serotyping for homotransplantation. XII. Occurrence of cytotoxic antibodies following kidney transplantation in man. Transplantation (1968) 6:392-9.
6. Claas FH, van Rood JJ. The hyperimmunized patient: from sensitization toward transplantation. Transpl Int (1988) 1:53-7. doi: 10.1007/BF00353819.
7. Talbot D. The flow cytometric crossmatch in perspective. Transpl Immunol (1993) 1:155-62. doi: 10.1016/0966-3274(93)90042-7.
8. Cook DJ, Terasaki PI, Iwaki Y, Terashita G, Fujikawa J, Gera J, et al. Flow cytometry crossmatching for kidney transplantation. Clin Transp (1988) 375-80.
9. Ogura K, Terasaki PI, Johnson C, Mendez R, Rosenthal JT, Ettenger R, et al. The significance of a positive flow cytometry crossmatch test in primary kidney transplantation. Transplantation (1993) 56:294-8. doi: 10.1097/00007890-199308000-00007.
10. Park MS, Terasaki PI, Bernoco D. Autoantibody against B lymphocytes. Lancet (1977) 2(8036):465-7. doi: 10.1016/s0140-6736(77)91598-7.
11. Lobo PI, Isaacs RB, Spencer CE, Pruett TL, Sanfey HA, Sawyer RG, McCullough C. Improved specificity and sensitivity when using pronase-digested lymphocytes to perform flow-cytometric crossmatch prior to renal transplantation. Transpl Int (2002) 15(11):563-9. doi: 10.1007/s00147-002-0469-y.
12. Freischlag K, Pearl MH, Chambers ET. The clinical impact of non-HLA antibodies in solid organ transplantation. Clin Transpl (2016) 32:31-43.
13. Gebel HM, Liwski RS, Bray RA. Technical aspects of HLA antibody testing. Curr Opin Organ Transplant (2013) 18:455-62. doi: 10.1097/MOT.0b013e32836361f1.
14. Zoet YM, Brand-Schaaf SH, Roelen DL, Mulder A, Claas FH, Doxiadis II. Challenging the golden standard in defining donor-specific antibodies: does the solid phase assay meet the expectations? Tissue Antigens (2011) 77:225-8. doi: 10.1111/j.1399-0039.2010.01608.x.

15. Tait BD. Detection of HLA antibodies in organ transplant recipients - triumphs and challenges of the solid phase bead assay. Front Immunol (2016) 7:570. doi: 10.3389/fimmu.2016.00570.
16. Gombos P, Opelz G, Scherer S, Morath C, Zeier M, Schemmer P, Susal C. Influence of test technique on sensitization status of patients on the kidney transplant waiting list. Am J Transplant (2013) 13:2075-82. doi: 10.1111/ajt.12332.
17. Tait BD, Susal C, Gebel HM, Nickerson PW, Zachary AA, Claas FH, et al. Consensus guidelines on the testing and clinical management issues associated with HLA and non-HLA antibodies in transplantation. Transplantation (2013) 95:19-47. doi: 10.1097/TP.0b013e31827a19cc.
18. Pandey P, Pande A, Kumar Devra A, Kumar Sinha V, Prasad Bhatt A. Comparative analysis of complement-dependent lymphocytotoxicity crossmatch and flow cytometry crossmatch results versus Luminex single-antigen bead-based donor-specific IgG class I antibody MFI values in live related renal transplant cases; a retrospective observation in 102 cases. J Immunoassay Immunochem (2021) 42:300-13. doi: 10.1080/15321819.2020.1862865.
19. Tambur AR, Campbell P, Claas FH, Feng S, Gebel HM, Jackson AM, et al. Sensitization in Transplantation: Assessment of Risk (STAR) 2017 Working Group Meeting Report. Am J Transplant (2018) 18:1604-14. doi: 10.1111/ajt.14752.
20. Buttigieg J, Ali H, Sharma A, Halawa A. Positive Luminex and negative flow cytometry in kidney transplantation: a systematic review and meta-analysis. Nephrol Dial Transplant (2019) 34:1950-60. doi: 10.1093/ndt/gfy349.
21. Mohan S, Palanisamy A, Tsapepas D, Tanriover B, Crew RJ, Dube G, et al. Donor-specific antibodies adversely affect kidney allograft outcomes. J Am Soc Nephrol (2012) 23:2061-71. doi: 10.1681/ASN.2012070664.
22. Sullivan HC, Gebel HM, Bray RA. Understanding solid-phase HLA antibody assays and the value of MFI. Hum Immunol (2017) 78:471-80. doi: 10.1016/j.humimm.2017.05.007.
23. Sullivan HC, Liwski RS, Bray RA, Gebel HM. The road to HLA antibody evaluation: Do not rely on MFI. Am J Transplant (2017) 17:1455-61. doi: 10.1111/ajt.14229.
24. Sullivan HC, Krummey SM, Gebel HM, Bray RA. The utility of second single antigen bead assay: Clearing the water or stirring up mud? Hum Immunol (2020) 81:663-70. doi: 10.1016/j.humimm.2020.09.002.
25. Tambur AR, Claas FH. HLA epitopes as viewed by antibodies: What is it all about? Am J Transplant (2015) 15:1148-54. doi: 10.1111/ajt.13192.
26. Morales-Buenrostro LE, Terasaki PI, Marino-Vazquez LA, Lee JH, El-Awar N, Alberu J. "Natural" human leukocyte antigen antibodies found in nonalloimmunized healthy males. Transplantation (2008) 86:1111-5. doi: 10.1097/TP.0b013e318186d87b.
27. Cai J, Terasaki PI, Anderson N, Lachmann N, Schonemann C. Intact HLA not beta2m-free heavy chain-specific HLA class I antibodies are predictive of graft failure. Transplantation (2009) 88:226-30. doi: 10.1097/TP.0b013e3181ac6198.
28. Huber L, Lachmann N, Niemann M, Naik M, Liefeldt L, Glander P, et al. Pretransplant virtual PRA and long-term outcomes of kidney transplant recipients. Transpl Int (2015) 28:710-9. doi: 10.1111/tri.12533.

29. Cecka JM. Calculated PRA (CPRA): the new measure of sensitization for transplant candidates. Am J Transplant (2010) 10:26-9. doi: 10.1111/j.1600-6143.2009.02927.x.
30. Mumford L, Fuggle SV, Martorell J, Slavcev A, Iniotaki A, Haasnoot GW, et al. A Europe wide acceptable mismatch program will enable transplantation of long waiting highly sensitised patients with a compatible donor. Transpl Immunol (2020) 64:101354. doi: 10.1016/j.trim.2020.101354.
31. Jackson KR, Covarrubias K, Holscher CM, Luo X, Chen J, Massie AB, et al. The national landscape of deceased donor kidney transplantation for the highly sensitized: Transplant rates, waitlist mortality, and posttransplant survival under KAS. Am J Transplant (2019) 19:1129-38. doi: 10.1111/ajt.15149.
32. Kransdorf EP, Pando MJ. Calculated panel reactive antibody with decimals: A refined metric of access to transplantation for highly sensitized candidates. Hum Immunol (2017) 78:252-6. doi: 10.1016/j.humimm.2016.12.009.
33. Keith DS. Parsing the 100% calculated panel reactive antibody kidney transplant candidates: Who gets transplanted? HLA (2020) 95:23-9. doi: 10.1111/tan.13692.
34. Tambur AR, Campbell P, Chong AS, Feng S, Ford ML, Gebel H, et al. Sensitization in transplantation: Assessment of risk (STAR) 2019 Working Group Meeting Report. Am J Transplant (2020) 20:2652-68. doi: 10.1111/ajt.15937.
35. Lachmann N, Todorova K, Schulze H, Schonemann C. Systematic comparison of four cell- and Luminex-based methods for assessment of complement-activating HLA antibodies. Transplantation (2013) 95:694-700. doi: 10.1097/TP.0b013e31827b3dc3.
36. Bouquegneau A, Loheac C, Aubert O, Bouatou Y, Viglietti D, Empana JP, et al. Complement-activating donor-specific anti-HLA antibodies and solid organ transplant survival: A systematic review and meta-analysis. PLoS Med (2018) 15:e1002572. doi: 10.1371/journal.pmed.1002572.
37. Lefaucheur C, Viglietti D, Bentlejewski C, Duong van Huyen JP, Vernerey D, Aubert O, et al. IgG donor-specific anti-human HLA antibody subclasses and kidney allograft antibody-mediated injury. J Am Soc Nephrol (2016) 27:293-304. doi: 10.1681/ASN.2014111120.
38. Gebel HM, Bray RA. A diagnostic 'C' saw: the ups and downs of C1q testing. Curr Opin Organ Transplant (2019) 24:402-10. doi: 10.1097/MOT.0000000000000659.
39. Hernandez D, Castro-de la Nuez P, Muriel A, Ruiz-Esteban P, Alonso M. Mortality on a renal transplantation waiting list. Nefrologia (2015) 35:18-27. doi: 10.3265/Nefrologia.pre2014.Oct.12681.
40. Prezelin-Reydit M, Combe C, Harambat J, Jacquelinet C, Merville P, Couzi L, Leffondre K. Prolonged dialysis duration is associated with graft failure and mortality after kidney transplantation: results from the French transplant database. Nephrol Dial Transplant (2019) 34:538-45. doi: 10.1093/ndt/gfy039.
41. Heidt S, Haasnoot GW, van Rood JJ, Witvliet MD, Claas FHJ. Kidney allocation based on proven acceptable antigens results in superior graft survival in highly sensitized patients. Kidney Int (2018) 93:491-500. doi: 10.1016/j.kint.2017.07.018.
42. Kosmoliaptsis V, Gjorgjimajkoska O, Sharples LD, Chaudhry AN, Chatzizacharias N, Peacock S, et al. Impact of donor mismatches at individual HLA-A, -B, -C, -DR,

and -DQ loci on the development of HLA-specific antibodies in patients listed for repeat renal transplantation. Kidney Int (2014) 86:1039-48. doi: 10.1038/ki.2014.106.
43. Wiebe C, Nickerson PW. Molecular mismatch - the renaissance of HLA in kidney transplantation. J Am Soc Nephrol (2020) 31:1922-5. doi: 10.1681/ASN.2020071011.
44. Wiebe C, Kosmoliaptsis V, Pochinco D, Taylor C, Nickerson P. A comparison of HLA molecular mismatch methods to determine HLA immunogenicity. Transplantation (2018) 102:1338-43.doi: 10.1097/TP.0000000000002117.
45. Kosmoliaptsis V, Bradley JA, Sharples LD, Chaudhry A, Key T, Goodman RS, Taylor CJ. Predicting the immunogenicity of human leukocyte antigen class I alloantigens using structural epitope analysis determined by HLAMatchmaker. Transplantation (2008) 85:1817-25. doi: 10.1097/TP.0b013e31817441d6.
46. Senev A, Coemans M, Lerut E, Van Sandt V, Kerkhofs J, Daniels L, et al. Eplet mismatch load and de novo occurrence of donor-specific anti-HLA antibodies, rejection, and graft failure after kidney transplantation: An observational cohort study. J Am Soc Nephrol (2020) 31:2193-2204. doi: 10.1681/ASN.2020010019.
47. Lachmann N, Niemann M, Reinke P, Budde K, Schmidt D, Halleck F, et al. Donor-recipient matching based on predicted indirectly recognizable HLA epitopes independently predicts the incidence of de novo donor-specific HLA antibodies following renal transplantation. Am J Transplant (2017) 17:3076-86. doi: 10.1111/ajt.14393.

Chapter 2: Comparison of Practices Across Europe for Dealing with Highly Sensitized Transplant Candidates

Siân Griffin*, on behalf of Workstream 06 of the Transplantation Learning Journey of the European Society of Organ Transplantation

Department of Nephrology, University Hospital of Wales, Cardiff, UK

** **Correspondence:** Siân Griffin, Sian.griffin2@wales.nhs.uk*

Keywords: sensitization, HLA antibodies, organ allocation, Europe, kidney sharing schemes

Word count (body text only): 1531
Number of tables: 1
Number of figures: 0

Abstract

The management of highly sensitized recipients is a challenge for all European countries, and initiatives have been introduced to increase the opportunities for this disadvantaged group to receive a transplant from either a deceased or living donor. Although there is some variability in approach between countries, there is broad recognition that to allow equity of access, novel strategies are needed. In the setting of deceased donor transplantation, this includes prioritization for highly sensitized patients should a compatible donor become available or the development of an acceptable mismatch program. For living donor transplantation, Kidney Exchange Programs have been established to allow compatible transplantation, and many individual units have undertaken antibody removal to allow HLA incompatible transplantation to proceed. Challenges remain, in particular to achieve a consensus on best practice and ensure there is the potential for all patients to receive a successful transplant.

1 Introduction

Organ transplantation has been one of the major medical advances of the 20th Century, providing life-saving treatment to millions. Assessment of sensitization and the detection of HLA antibodies has become increasingly sophisticated, and potential recipients now have a detailed antibody profile compiled prior to transplantation. Around one in 4 potential kidney transplant candidates are highly sensitized, limiting their available donor pool and increasing their waiting time before receiving a transplant. The barrier of sensitization is a frustrating situation for both clinicians and transplant candidates, but fortunately several options are now available to increase transplant opportunities for this group. In addition to the interventions developed in single centers, there have also been national initiatives to facilitate deceased organ donation, and a rapidly increasing number of countries have established Kidney Exchange Programs (KEP) for those with a living donor. Living donation offers the greatest opportunity for treatment to modulate antibody

levels, but this has also been applied in the context of deceased organ donation. There is considerable variability in practice across Europe, not only in terms of rates of deceased and living donation, but also in approaches to antibody removal and access to a KEP. The best outcome for an individual recipient depends both on their degree of sensitization and risk stratification, and the availability of different treatment options in their locality.

2 Organization of Transplantation in Europe

Both deceased and living donations are coordinated on either a national basis (for example the United Kingdom, France, Switzerland, Italy, Spain and Portugal), or on behalf of a group of countries (http://www.accord-ja.eu/background). Eurotransplant (https://www.eurotransplant.org/) is responsible for allocation of donor organs in Austria, Belgium, Croatia, Germany, Hungary, Luxembourg, the Netherlands and Slovenia. Scandiatransplant (http://www.scandiatransplant.org/) is the organ exchange organization for Denmark, Finland, Iceland, Norway, Sweden and Estonia. Larger donor pools would be expected to increase the likelihood of identifying a compatible donor for those who are hard to match, either due to an unusual tissue type or a high level of sensitization. An informal survey of transplant practices around Europe was carried out during September and October 2021, and the results form Table 1.

3 Deceased Organ Offering and Allocation

Deceased donor allocation schemes that do not take HLA sensitization into account will inevitably lead to fewer offers being made to these candidates and longer waiting times. Offering schemes can adjust for this bias, either by increasing the weighting given to those who are hard to match, as in the UK Kidney Offering Scheme (https://www.odt.nhs.uk/transplantation/kidney/kidney-offering-and-matching/) or by the development of an Acceptable Mismatch (AM) program, first established by Eurotransplant over 30 years ago (1). The Eurotransplant AM program has enabled successful transplantation of highly sensitized patients (HSP) with excellent outcomes (2). Despite this success, a subgroup of patients will not receive the offer of a transplant because a suitable donor is not available in the Eurotransplant donor population.

A similar observation has been made in other transplant organizations in North America (3, 4). The EUROSTAM project (a Europe-wide Strategy to enhance Transplantation of highly sensitized patients on the basis of Acceptable HLA Mismatches) has compared data from five European registries (Eurotransplant, UK National Health Service Blood and Transplant, Barcelona, Athens and Prague), to determine whether expanding the donor pool across different populations will result in increased rates of transplantation for those with >95% sensitization (5). In total, 195 (27%) of the 724 HSP who had been registered for at least 5 years at each organization had an increased chance of a compatible kidney transplant offer in a different European pool. This makes a strong case for sharing kidneys between European countries for selected difficult to transplant patients.

4 Living Donor Transplantation - KEPs

Europe's first kidney exchange was carried out in Switzerland in 1999 (6), however, the Netherlands was the first country to establish a nationally coordinated KEP in 2004 (7). The UK Kidney Sharing Schemes (KSS) were initiated in 2007 (8), and to date this

program has performed the greatest number of transplants (9). The Spanish national program began in 2009. Over the last decade, there has been a further rapid expansion in the number of programs, which are now established in Austria, the Czech Republic, Poland, Belgium, France, Italy and Portugal, and are developing elsewhere (9).

The active KEPs are organized centrally. Approaches to living donation vary between countries (10), which has an impact on the number of donors enrolled and the chance of an HSP receiving an offer. For example, in the UK, altruistic donation is permitted, and all altruistic donors are enrolled in the KSS to initiate donor chains. The inclusion of compatible pairs is also permitted, with the most usual reasons for consideration of inclusion being a significant age difference between donor and recipient and poor HLA match. In addition to short chains, the UK scheme also allows three-way exchanges. Altruistic donation is not possible in France, Poland, Greece and Switzerland. In France, only two-way exchanges are possible, and in France and Portugal only incompatible pairs can participate (9).

The Austrian and Czech programs both commenced in 2011, and merged in 2015, including the option for altruistic donor-initiated chains (11). This transnational merger has demonstrated the feasibility of increasing the size of the donor pool, although while matching rates in Austria doubled, those in the Czech program actually fell, partly due to the introduction of more stringent threshold criteria for HLA antibodies. Further collaborations have been introduced between Italy, Portugal and Spain (12), and Sweden, Norway and Denmark, although these collaborations have mainly considered patients left unmatched in their national KEPs.

The European Network for Collaboration on Kidney Exchange Programs (ENCKEP, https://www.eurotransplant.org/) was established in 2016 “in order to establish and foster a preferential discussion channel for the various essential themes that have to be addressed for the implementation of a collaborative KEP”. The program has contributed to aspirations for future developments, including modeling of European KEPs with the aim of future optimization (13).

5 HLA Incompatible Transplantation

Rates of HLA incompatible (HLAi) transplantation vary considerably between centers and countries, depending on the availability of alternative approaches, likelihood of achieving a compatible transplant, the clinician’s interpretation of the individual patient’s risk and the acceptability of the proposed antibody removal regimen and predicted outcome to the recipient.

No country has a published national consensus on their optimal recommended management pathway for HSP. Several European centers have published their protocols and outcomes following HLAi transplantation (14–17), but a more general overview of how widely this option is offered is not available. A recent survey of European transplant centers demonstrated substantial variability in the definition of sensitization, approaches to improve opportunities for deceased and living transplantation and perceived success of HLAi transplantation. This was an informal survey carried out by the European Society of Organ Transplantation, which queried European transplantation professionals regarding approaches to patients with HLA antibodies. There were 47 responses from 25 European countries (21 complete responses). The majority of respondents (>80%)

agreed that new strategies were needed to more effectively manage highly sensitized transplant candidates.
In the UK, the recognition that results following HLAi transplantation may be inferior to compatible (18), albeit with similar recipient survival, has led to increased reliance on the KSS. Although the success of the KSS has been to the benefit of many, there are a subgroup of patients who have little chance of receiving a compatible transplant and at present may not be offered the opportunity and potential benefit of an HLAi transplant (19).

6 Conclusion

The barrier of sensitization remains a significant hurdle for many transplant candidates. In the future, on-going research will improve the accuracy of risk stratification for HLAi transplantation, and prospective data collection of patient outcomes from the time of initiation of dialysis will contribute to more informed decision making by transplant candidates and their clinicians.

7 Recommendations

Organ Allocation

- We recommend all countries and centers have an active policy of priorizing highly sensitized patients for organ transplantation (also Chapter 4, Desensitization strategies)
- Access to the donor pool should be increased through greater use of:
  - Increased access to and harmonization of Kidney Exchange Programs, with greater and standardized sharing of outcomes (Chapters 2 and 6)
  - Inclusion of unspecified kidney donations (if these are performed), in kidney sharing schemes (Chapters 2 and 6)

Areas for Further Research

We recommend that data be collected prospectively for sensitized patients, in order to compare the effect of an HLA incompatible transplant with remaining on the waiting list. This data should include:

- Mortality
- Morbidity
- Quality of Life (Chapters 2 and 5)

Abbreviations

AM, acceptable mismatch; ENKAP, European Network for Collaboration on Kidney Exchange Programs; EUROSTAM project; a Europe-wide Strategy to enhance Transplantation of highly sensitized patients on the basis of Acceptable HLA Mismatches); HLA, human leukocyte antigen; HLAi, incompatible HLA antigens; HSP, highly sensitized patient; KEP, kidney exchange program; KSS, kidney sharing scheme

Author Contributions

The members of WS06 of ESOT provided input and critical review of this chapter: Oriol Bestard, Frans Claas, Lucrezia Furian, Christophe Legendre, Nizam Mamode, Maarten Naesens, Liset Pengel.

Conflict of Interest

The author declares that the research was conducted in the absence of any commercial or financial relationships that could be construed as a potential conflict of interest.

Acknowledgments

The author gratefully acknowledges the kind assistance of Professor Baris Akin (Demiroglu Bilim University Florence Nightingale Hospital, Istambul, Turkey), Professor John Boletus (National and Kapodistrian University of Athens, Greece), Professor Peter Conlon (Beaumont Hospital, Dublin, Ireland), Dr Caterina Di Bella (University Hospital of Padua, Italy), Associate Professor Piotr Domagala (Medical University of Warsaw, Poland), Mr Frank Dor (Imperial College NHS Healthcare Trust, London, UK), Dr Farsad-Alexander Eskandary (Medical University Vienna, Austria), Professor Fadi Haidar (HUG, Switzerland), Assistant Professor Nataša Katalinić (University of Rijeka, Croatia), Professor Christophe Legendre (Necker Hospital, Paris, France), Dr Gabriel Oniscu (Edinburgh Transplant Centre, UK), Professor Vassilios Papalois (Hammersmith Hospital, Imperial College, London, UK), Professor Søren Schwartz Sørensen (Copenhagen University Hospital, Denmark), Dr Maria Simonenko (Almazov National Medical Research Centre, St Petersburg, Russia); Professor Ondrej Viklicky (Transplant Center, Prague, Czech Republic), Professor Stela Zivcić-Cosi (University Hospital Center Rijeka, Croatia), Assistant Professor Renata Žunec (University Hospital Center, Zagreb, Croatia) for providing information on current practice in each of their countries for Table 1.

TABLE 1. Informal European Survey of Practices Regarding Transplantation, 2021

Country or organization for deceased donor allocation	Population (million)	Living Donation	Deceased Donation		
		**Is there access to a Kidney Exchange Program?**	**Does the allocation scheme include prioritization for sensitized recipients?**	**Does the allocation scheme include an Acceptable Mismatch program?**	**Details**
Eurotransplant (Austria, Belgium, Croatia, Germany, Hungary, Luxembourg, the Netherlands, Slovenia)	137	Yes: Austria (with the Czech Republic and Israel), Belgium[20], the Netherlands	Yes	Yes	Acceptable antigens are defined by the lack of antibody-reactivity in complement-dependent cytotoxicity assays using target cells mismatched for a single HLA antigen, or single antigen-expressing cell lines
ScandiaTransplant (Denmark, Finland, Iceland, Norway, Sweden, Estonia)	28.9	ScandiaTransplant Kidney Exchange Program launched April 2019	Yes	Yes, ScandiaTransplant Acceptable Mismatch Program (STAMP)[a]	Common waiting list and database system. STAMP patients have the highest priority for a deceased donor kidney[b]
Czech Republic	10.7	Yes<br>Recent expansion to include Austria and Israel	Yes	No	Patients are categorized according to their measured PRA: 0–20%, 20–80, and >80%, with higher priority for

Country or organization for deceased donor allocation	Population (million)	Living Donation	Deceased Donation		
		Is there access to a Kidney Exchange Program?	Does the allocation scheme include prioritization for sensitized recipients?	Does the allocation scheme include an Acceptable Mismatch program?	Details
					transplantation given to those with higher PRA values. Patients who have waited longer than 3 years for a transplant are prioritized, regardless of their PRA value<br>DSA are allowed, based on local protocols for desensitization
France	67	Yes	Yes	Yes	Sensitized patients are prioritized according to waiting time and HLA compatibility
Greece	10.4	Yes	Yes	Yes	Patients are prioritized based on waiting time and HLA mismatch
Ireland	5	Yes – with the UK	Yes	Yes	All<br>highly sensitized patients who are long waiting are screened to identify acceptable mismatches or windows in which they can be transplanted

Country or organization for deceased donor allocation	Population (million)	Living Donation	Deceased Donation		
		Is there access to a Kidney Exchange Program?	Does the allocation scheme include prioritization for sensitized recipients?	Does the allocation scheme include an Acceptable Mismatch program?	Details
Italy	60.3	Yes	Yes	Yes	The Italian national allocation scheme prioritizes at national level patients with PRA >90% and who have been on dialysis >8 years.<br>Recipients are selected according to a points score, based on:<br>- PRA<br>- Age mismatch between donor and recipient<br>- Recipient age<br>- HLA mismatch<br>- Time spent on dialysis<br>- Time on waiting list
Latvia	1.9	Yes[21]			
Lithuania	2.9	Yes[22] established in 2013, although up to 2019, the system has not been used			Although Lithuania is not a member of international organ procurement and allocation organizations yet, they do collaborate with neighboring Nordic countries and exchange

**Country or organization for deceased donor allocation**	**Population (million)**	**Living Donation**	**Deceased Donation**		
		**Is there access to a Kidney Exchange Program?**	**Does the allocation scheme include prioritization for sensitized recipients?**	**Does the allocation scheme include an Acceptable Mismatch program?**	**Details**
					organs with Latvia, Estonia and Poland
Poland	38	Yes	Yes	Yes	Prioritization for patients with a PRA >80%; increased weighting for patients with PRA 50–79.
Portugal	10.2	Yes	Yes	No	Additional points for sensitized and highly sensitized patients
Russia	146.2		No<br>Each transplant center has their own internal protocol	Yes<br>Some kidney centers may transplant if there is an acceptable mismatch	There is no common waiting list in Russia or any kind of program like Eurotransplant. Each center has its own waiting list, their own algorithm for prioritizing patients for transplantation (although many use UNOS, Intermax or other classification systems to help decisions) and their own protocol for post-transplant follow-up. Prioritization is based on donor and recipient risk

<table>
<tr><th rowspan="2">Country or organization for deceased donor allocation</th><th rowspan="2">Population (million)</th><th>Living Donation</th><th colspan="3">Deceased Donation</th></tr>
<tr><th>Is there access to a Kidney Exchange Program?</th><th>Does the allocation scheme include prioritization for sensitized recipients?</th><th>Does the allocation scheme include an Acceptable Mismatch program?</th><th>Details</th></tr>
<tr><td></td><td></td><td></td><td></td><td></td><td>index match, waiting time, and HLA mismatch</td></tr>
<tr><td>Slovakia</td><td>5.4</td><td>No</td><td>No</td><td>No</td><td></td></tr>
<tr><td>Spain</td><td>46.8</td><td>Yes</td><td>Yes</td><td>No</td><td>One kidney of all brain death donors is offered to a National Prioritization Scheme for sensitized patients with a cPRA >98%. Kidney acceptance for an individual patient based on virtual crossmatch[23]</td></tr>
<tr><td>Switzerland</td><td>8.74</td><td>Yes</td><td>Yes</td><td>Yes</td><td>Prioritization for allocation is based on a continuum of increasing cPRA for each blood group. An MFI cut-off of 1000 is used for both class 1 and class 2 DSA</td></tr>
<tr><td>Turkey</td><td>85.6</td><td>Yes</td><td>No</td><td>No</td><td>Allocation is according to a scoring system:
<table>
<tr><th>Criteria</th><th>Score</th></tr>
<tr><td>HLA match</td><td>DR 150, B 50, A 5</td></tr>
<tr><td>Region</td><td>1000</td></tr>
<tr><td>Center</td><td>250</td></tr>
</table></td></tr>
</table>

<table>
<tr><th rowspan="2">Country or organization for deceased donor allocation</th><th rowspan="2">Population (million)</th><th>Living Donation</th><th colspan="3">Deceased Donation</th></tr>
<tr><th>Is there access to a Kidney Exchange Program?</th><th>Does the allocation scheme include prioritization for sensitized recipients?</th><th>Does the allocation scheme include an Acceptable Mismatch program?</th><th>Details</th></tr>
<tr><td></td><td></td><td></td><td></td><td></td><td><table><tr><td>Recipient age (<11 years / 12-17 / ≥18 years</td><td>HLA match score multiplied by 2.5 / 1.5 / 1</td></tr><tr><td>Time on dialysis</td><td>3 points for each month</td></tr></table></td></tr>
<tr><td>United Kingdom</td><td>68</td><td>Yes</td><td>Yes</td><td>No</td><td>Absolute priority for those with cRF >100%, matchability score 10, waiting time >7 years
Remaining patients prioritized on points score, based on:
i. Donor and recipient risk index match
ii. Waiting time
iii. HLA mismatch
iv. Local region > non-local regions</td></tr>
</table>

**Country or organization for deceased donor allocation**	**Population (million)**	**Living Donation**	**Deceased Donation**		
		**Is there access to a Kidney Exchange Program?**	**Does the allocation scheme include prioritization for sensitized recipients?**	**Does the allocation scheme include an Acceptable Mismatch program?**	**Details**
					(of four national regions)

a. http://www.scandiatransplant.org/organ-allocation/Manual_STAMP_20_nov_2017_version_8.1.pdf
b. http://www.scandiatransplant.org/organ-allocation/Kidney_exchange_11_november_2020.pdf.

8. References

1. Heidt S, Witvliet MD, Haasnoot GW, Claas FH. The 25th anniversary of the Eurotransplant Acceptable Mismatch program for highly sensitized patients. Transpl Immunol (2015) 33:51-7. doi: 10.1016/j.trim.2015.08.006.
2. Heidt S, Haasnoot GW, van Rood JJ, Witvliet MD, Claas FHJ. Kidney allocation based on proven acceptable antigens results in superior graft survival in highly sensitized patients. Kidney Int (2018) 93:491-500. doi: 10.1016/j.kint.2017.07.018.
3. Bingaman AW, Murphey CL, Palma-Vargas J, Wright F. A virtual crossmatch protocol significantly increases access of highly sensitized patients to deceased donor kidney transplantation. Transplantation (2008) 86:1864-8. doi: 10.1097/TP.0b013e318191404c.
4. Bray RA, Brannon P, Breitenbach C, Bryan C, Chen DF, Lai J, et al. The new OPTN kidney allocation policy: potential for inequitable access among highly sensitized patients. Am J Transplant (2015) 15:284-5. doi: 10.1111/ajt.13061.
5. Mumford L, Fuggle SV, Martorell J, Slavcev A, Iniotaki A, Haasnoot GW, et al. A Europe wide acceptable mismatch program will enable transplantation of long waiting highly sensitised patients with a compatible donor. Transpl Immunol (2020) 64:101354. doi: 10.1016/j.trim.2020.101354.
6. Hadaya K, Fehr T, Rusi B, Ferrari-Lacraz S, Jean V, Ferrari P. Kidney paired donation: a plea for a Swiss National Programme. Swiss Med Wkly (2015) 145:w14083. doi: 10.4414/smw.2015.14083.
7. de Klerk M, Keizer KM, Claas FH, Witvliet M, Haase-Kromwijk BJ, Weimar W. The Dutch national living donor kidney exchange program. Am J Transplant (2005) 5:2302-5. doi: 10.1111/j.1600-6143.2005.01024.x.
8. Johnson RJ, Allen JE, Fuggle SV, Bradley JA, Rudge C, Kidney Advisory Group UKTN. Early experience of paired living kidney donation in the United kingdom. Transplantation (2008) 86:1672-7. doi: 10.1097/TP.0b013e3181901a3d.
9. Biro P, Haase-Kromwijk B, Andersson T, Asgeirsson EI, Baltesova T, Boletis I, et al. Building kidney exchange programmes in Europe - An overview of exchange practice and activities. Transplantation (2019) 103:1514-22. doi: 10.1097/TP.0000000000002432.
10. Burnapp L, Van Assche K, Lennerling A, Slaats D, Van Dellen D, Mamode N, et al. Raising awareness of unspecified living kidney donation: an ELPAT view. Clin Kidney J (2020) 13:159-65. doi: 10.1093/ckj/sfz067.
11. Viklicky O, Krivanec S, Vavrinova H, Berlakovich G, Marada T, Slatinska J, et al. Crossing borders to facilitate live donor kidney transplantation: the Czech-Austrian kidney paired donation program - a retrospective study. Transpl Int (2020) 33:1199-210. doi: 10.1111/tri.13668.
12. Valentin MO, Ruiz JC, Vega R, Martin C, Matesanz R, working group P. Implementation of a national priority allocation system for hypersensitized patients in Spain, based on virtual crossmatch: Initial results. Transplant Proc (2016) 48:2871-5. doi: 10.1016/j.transproceed.2016.09.024.
13. Biró P, van de Klundert J, Manlove D, Pettersson W, Andersson T, Burnapp L, et al. Modelling and optimisation in European kidney exchange programmes. Eur J Oper Res (2021) 291:447-56. doi:10.1016/j.ejor.2019.09.006

14. Higgins R, Lowe D, Hathaway M, Williams C, Lam FT, Kashi H, et al. Human leukocyte antigen antibody-incompatible renal transplantation: excellent medium-term outcomes with negative cytotoxic crossmatch. Transplantation (2011) 92:900-6. doi: 10.1097/TP.0b013e31822dc38d.
15. McCaughan JA, Courtney AE. Successful kidney transplantation in highly sensitized, ultra-long-term dialysis patients. Transpl Int (2017) 30:844-6. doi: 10.1111/tri.12970.
16. Amrouche L, Aubert O, Suberbielle C, Rabant M, Van Huyen JD, Martinez F, et al. Long-term outcomes of kidney transplantation in patients with high levels of preformed DSA: The Necker High-Risk Transplant Program. Transplantation (2017) 101:2440-8. doi: 10.1097/TP.0000000000001650.
17. Marks WH, Mamode N, Montgomery RA, Stegall MD, Ratner LE, Cornell LD, et al. Safety and efficacy of eculizumab in the prevention of antibody-mediated rejection in living-donor kidney transplant recipients requiring desensitization therapy: A randomized trial. Am J Transplant (2019) 19:2876-88. doi: 10.1111/ajt.15364.
18. Manook M KL, Ahmed Z, Robb M, Johnson R, Shaw O, Kessaris N, Dorling A, Mamode, N Post-listing survival for highly sensitised patients on the UK kidney transplant waiting list: a matched cohort analysis. Lancet (2017) 389:8. doi: 10.1016/S0140-6736(16)31595-1.
19. Manook M, Johnson R, Robb M, Burnapp L, Fuggle SV, Mamode N. Changing patterns of clinical decision making: are falling numbers of antibody incompatible transplants related to the increasing success of the UK Living Kidney Sharing Scheme? A national cohort study. Transpl Int (2021) 34:153-62. doi: 10.1111/tri.13776.
20. Detry O, Van Deynse D, Van Vlierberghe H, Pirenne J, on behalf of the Belgian Transplantation Society (BTS). Organ procurement and transplantation in Belgium, Transplantation (2017) 101:1953-5. doi: 10.1097/TP.0000000000001866
21. Ziedina I, Jushinskis J, Lejniece S, Strike E. Organ donation and transplantation in Latvia, Transplantation (2019) 103:2211-3. doi: 10.1097/TP.0000000000002764
22. Miglinas M, Vaiciuniene R, Vickiene A. Organ transplantation in Lithuania, Transplantation (2019) 103:1287-9. doi: 10.1097/TP.0000000000002575
23. Valentin M, Mahillo B, Martinez I, Garcia M, Ormeño M, Calderari, E, et al. Living kidney donation in Spain, a global strategy to increase this modality of transplantation. Transplantation (2018) 102:S133. doi: 10.1097/01.tp.0000542751.82168.ad

Chapter 3: Strategies for Access to Kidney Transplantation in Highly Sensitized Patients

Oriol Bestard,[1*] Maarten Naesens,[2] Nizam Mamode[3] on behalf of Workstream 06 of the Transplantation Learning Journey of the European Society of Organ Transplantation
[1]*Department of Nephrology and Kidney Transplantation, Vall d'Hebrón University Hospital, Barcelona, Spain*
[2]*Department of Microbiology, Immunology and Transplantation, KU Leuven, Leuven, Belgium*
[3]*Department of Transplant Surgery, Guys Hospital, London, UK*

**** Correspondence:*** *Oriol Bestard, obestard@vhebron.net*

Keywords: humoral immunity, sensitization, access to transplantation

Word count (body text only): 3367
Number of figures: 8
Number of tables: 0

Abstract
Highly sensitized patients face a longer-than-average wait for a kidney transplant. Strategies to maximize the opportunity of an HLA-compatible transplant for these patients include priority points systems in deceased-donor kidney-allocation programs, living-donor kidney-paired exchange programs and acceptable mismatch programs. Despite these measures, patients with exotic HLA types, antibodies against very frequent HLA antigens or a cPRA of 100% tend to remain on the transplant wait list with a very low chance of receiving an organ offer. An HLA incompatible transplant should be considered for those in whom these strategies are unlikely to yield a transplant, and the use of online calculators may help in this. This chapter explores the strategies for successfully matching highly sensitized patients with donor organs, looking particularly at how immunological risk can be defined and minimized for an individual patient.

1 Introduction

A main barrier to successful transplantation is the Human Leukocyte Antigen (HLA) molecular disparity between donors and recipients, which triggers a robust recall alloimmune response, ultimately leading to allograft rejection and graft loss (1). Notably, previous HLA antigen encounters through blood transfusions, pregnancies or previous transplants may lead to the development of a long-lasting allogeneic immune memory, mostly characterized by the presence of serum IgG antibodies directed to distinct HLA antigens (2). With currently available immunological tools, detection of circulating anti-HLA antibodies can be accurately assessed and thus, transplant candidates may be stratified by their immunological risk of humoral rejection of a transplant organ. While the outstanding sensitivity and specificity of these assays in detecting serum antibodies has allowed a clear reduction of severe, hyperacute antibody-mediated rejection, a

progressively increasing proportion of kidney transplant candidates worldwide may be considered as highly sensitized to HLA antigens. These patients have a significantly lower chance of finding an HLA compatible kidney organ donor and remain for longer periods of time on the waiting list for transplantation. Importantly, a precise understanding of the different biological features of circulating anti-HLA antibodies according to different immunological tests, will determine their clinical relevance in predicting the precise risks of post-transplant allograft rejection and survival. Some patients have immunological and serological memory without current circulating antibodies detectable in the peripheral blood. There are currently no clinically validated and available tools that accurately assess such memory responses. In any case, the risk associated with these responses seems lower than with current circulating antibodies. The risk for a booster reaction after transplantation exists, but is not predictable. It is therefore difficult to propose well-substantiated recommendations for this type of risk.

For highly sensitized patients with present circulating HLA antibodies, desensitization therapies have shown poorer mid-/long-term transplant outcomes than seen in those undergoing HLA compatible transplants. In light of this, in recent years, transplant physicians have developed a number of strategies, both for deceased and living-donor transplantation, aimed at facilitating access to HLA compatible transplantation for these highly sensitized patients before they undergo desensitization therapies.

Among the most successful transplant policies are i) sliding scales - local, regional or national priority points programs; ii) establishing an allocation system based on acceptable mismatch (AM) HLA antigens rather than in the avoidance of unacceptable ones to improve donor/recipient matching; iii) favoring different living donor kidney transplantation modalities to achieve HLA compatibility, such as overcoming ABO incompatibility or kidney paired donation exchange programs.

In this chapter we discuss the different approaches to establish a definition of the immunological risk of a transplant candidate, as well as different major straightforward strategies to increase transplant rates in highly immunized transplant candidates.

2 Stratification of the Immunological Risk of Kidney Transplant Candidates

The molecular basis of the HLA system relies on a highly polymorphic system that allows for strong adaptive immune responses driven both by alloreactive T and B lymphocytes. However, while alloreactive T cells are key players promoting and facilitating allograft rejection, there is a lack of sensitive and validated immune tools that can be implemented in clinical transplantation to mitigate these effects (3, 4). Conversely, current immune-risk stratification in kidney transplant candidates is solely focused on the humoral effector pathway of adaptive immunity through the detection of serum anti-HLA antibodies directed against donor antigens using a plethora of highly sensitive *in vitro* immune assays (please see **Chapter 1,** Definition of sensitization).

Since a high number of unacceptable alloantigens diminishes the likelihood of an organ offer, precise identification of circulating anti-HLA antibodies is highly warranted. In addition, the risk of undergoing kidney transplantation should be balanced with the risk of post-transplant rejection, allograft survival, as well as life expectancy and quality of life while remaining on the transplant waiting list for an extended period of time.

It is widely accepted that patients with a positive complement-dependent cytotoxicity (CDC) crossmatch test, targeting complement-fixing class I and/or II donor-specific antibodies (DSA), have a very high risk of hyperacute rejection and graft failure (5). With the implementation of flow-cytometric crossmatching (FCM), serum DSA against either class I or II donor antigens may be more accurately detected than by CDC, reducing the high risk of early post-transplant acute antibody-mediated rejection. However, a positive FCM in the absence of detectable DSA by single antigen bead (SAB) assays may not necessarily be predictive of rejection (6, 7), most likely because these antibodies may recognize antigens not present at the endothelial cell surface. Notably, with the development of highly sensitive SAB assays, the identification of unacceptable antigens has become easier, but entails a high degree of interpretation and expertise in each laboratory. SAB assays detect amounts of antibodies present in the serum (and quantified as Mean Fluorescence Intensity [MFI]), and can identify purified class I and II antigens adhered to plastic beads with fluorescent-labeled antibodies to IgG, thus providing a reliable virtual crossmatch, which does not require donor cells. Several modifications of SAB assays have been developed, including an assay to evaluate complement-binding DSA (both C1q or C3d), although their absence does not rule out the negative impact of DSA (8 –10). IgG subclasses can also be delineated and have also been associated with a diverse range of severity of graft damage due to their complement binding potential (11), as well as their Fcγ receptors, which trigger innate immune responses (12). The impact of preformed DSA has classically been linked to the degree of MFI, although there is no general consensus regarding MFI cut-off levels (as discussed in **Chapter 1**, Definition of sensitization). Notably, it is important to bear in mind that a number of distinct factors may impact the interpretation of SAB data, such as antibody titer, prozone effect, competition of shared epitopes on different beads, as well as irrelevant antibody reactivity against denatured HLA molecules (13–15). Thus, the ability of DSA identified by SAB to bind donor cells *ex vivo* in FCM is a good predictor of subsequent antibody-mediated rejection lesions and graft loss (in 50% and 30% of recipients, respectively (16–18)). Importantly, by accepting every SAB signal, a high number of patients would be defined as highly sensitized, with the consequently lower chance of receiving an organ offer through regular allocation systems – likely reducing a patient's chance by up to five-fold (5). Therefore, an individualized risk-assessment of previous sensitizing events, adding a thorough epitope analysis and most importantly, the likelihood of receiving an HLA compatible transplant in their respective region, should be taken into account.

Indeed, there is still no precise definition of the different strata for the humoral risk in kidney transplantation, which ultimately represents a major barrier to evolve clinical care in this area. Currently, a wide range of different patient profiles are mixed together. Aiming to move this field forward, a European working group endorsed by the European Society of Organ Transplantation (ENGAGE), has put forward an initiative proposing an integrative consensus of the most consistent evidence to stratify kidney transplant candidates into five distinct risk categories with the aim of conferring the best chance of successful transplantation. These risk categories take into account an individual patient's past immunological clinical background, integrated with an assessment of serological alloimmune memory using CDC-crossmatch, FC-crossmatch and SAB assays (19) (**Figure 1**). While further novel technologies assessing the impact of other immune

effector pathways favoring transplant rejection are in development and validation, these different immunological humoral-risk categories should help stratify the risks of kidney transplant candidates.
The high humoral immune-risk, together with higher mortality rates of patients remaining on the waiting list for long periods of times (20, 21), have prompted a number of strategies to be put forward worldwide aiming to increase transplantation rates in highly sensitized kidney transplant candidates.

3 Sliding Scale Priority Points for Deceased-Donor Kidney Transplantation

A widely established policy to enhance the transplant rate of highly sensitized kidney transplant candidates is based on the implementation of a local, regional or national sliding scale priority points system for the regular allocation of deceased donor organs. The aim is to increase the pool of suitable donors with compatible kidneys based on a virtual crossmatch. These systems award extra points based on calculated panel-reactive antibody (cPRA) and by implementing local, regional, and national sharing for those with a high calculated cPRA, which may vary between different countries. The kidney allocation system promoted in the United States, helps patients from a starting cPRA threshold of 20%. Those with a cPRA ≥98% receive a higher sliding scale priority point score, in which ABO incompatible (A2/A2B to B organ) offers are also permitted due to their lower immunogenicity, and these patients are eligible for local, regional and national priority donor allocation (22–24). Remarkably, kidney transplant rates among these patients dramatically increased from 2.5% to 13.4% during the first year after implementation, notwithstanding an important bolus effect (25). Consequently, the median waiting time dropped from >19 years to 3.2 years (26). The implementation of this kidney allocation system also increased sharing of high Kidney Donor Profile Index kidneys and decreased the hazard of graft loss without an impact on patient survival (27). A similar scheme has been developed in Spain, with a national sliding scale priority program using an ABO identical deceased organ donor allocation system (PATHI) (28). However, while these programs have significantly helped the access to transplantation for this increasingly prevalent patient population, these outcomes only hold true for those transplant candidates with a cPRA <100% (25, 29, 30). For those with 100% cPRA, sliding priority points schemes do not seem to increase their chance of receiving a kidney transplant, or even an organ offer, especially when stratifying the levels of sensitization into decimals (99.95–100%) (31), **Figure 2**.
An illustrative example of how the interpretation of the SAB cut-offs defining unacceptable HLA antigens may directly impact on access to transplantation was clearly reported by Houp and colleagues. This group showed that including very weak MFI levels of anti-HLA antibodies as unacceptable antibodies in intermediately sensitized patients, deleteriously impacted on severely sensitized patients competing for similar priority organ donors (30). Importantly, excellent short-term kidney graft and patient outcomes under this new priority system have been reported, with acceptable low rejection rates. Although the organs had longer cold ischemia time and subsequently a higher incidence of delayed graft function, this did not negatively impact graft outcomes (25, 32). Whether long-term graft survival will mimic the short-term outcomes still remains to be evaluated and certain concerns have been raised. These relate to the generally low donor/recipient HLA matching for

these patients – using an unacceptable HLA antigen policy rather than an acceptable antigen mismatch program thus eventually leading to poorer long-term graft outcomes (33, 34).
In summary, while sliding scale priority points strategies have enabled highly sensitized transplant candidates to have access to kidney transplantation, showing optimal short/mid-term graft outcomes, important questions still remain. Patients with the highest sensitization status (cPRA 100%) do not seem to be positively impacted, with their chance of receiving a transplant offer remaining extremely low. Furthermore, whether HLA matching within these programs should emphasize donor/recipient acceptable antigen matching rather than concentrating on prohibited ones to ultimately gain longer transplant survival rates is unclear.

4 Living-Donor Kidney Sharing Scheme (KSS) / ABOi but HLA Compatible

For those patients with an antibody incompatible (ABO or HLA) living donor, a kidney sharing scheme (KSS) remains an option. This is discussed in detail in **Chapter 6** (The place of kidney sharing schemes for sensitized patients), but one of the difficulties faced by clinicians is assessing the likelihood of success through a KSS for an individual patient. This will clearly vary according to a number of factors:

- National demographics: the incidence of blood groups and HLA types varies across different countries, and will therefore affect the chances within a KSS - for example, where blood group B is uncommon (as in most Western European countries) the chance for group B recipients will be lower, **Figure 3**
- The size of the scheme: generally speaking, the larger the scheme the greater the chances of a match, although there is probably a maximum size beyond which there is no incremental advantage
- Recipient characteristics: for example, those who are very highly sensitized (eg cPRA/CRF 100%) will have a low or even negligible chance in a KSS, for the same reasons that they will have a low chance of receiving a deceased donor transplant
- KSS algorithm: each KSS will have its own algorithm, which will affect the chances an individual has for a match in the scheme. This should be considered when entering a patient into the scheme.

The easiest way to address these factors is to access an online calculator which incorporates the factors into a probability of a match, ideally with confidence intervals. An example from the UK scheme is given at https://www.odt.nhs.uk/living-donation/tools-and-resources and at https://www.odt.nhs.uk/transplantation/tools-policies-and-guidance/calculators/, which addresses the likelihood of a deceased donor transplant for sensitized patients.
Finally, an important point to consider is that entry into a KSS should not be considered as a definitive solution. Figures from the UK KSS show that the incremental chance of a match after 6 or 7 'runs' is low, and thus, at this stage, if there are alternatives, such as a direct antibody incompatible transplant, these should be considered, **Figure 4**.

5 Acceptable Mismatch Program

Although a priority point strategy is anticipated to improve access to transplantation for sensitized patients, this is not necessarily helpful for the most highly sensitized patients,

who may still have difficulty securing a transplantation. While desensitization strategies (See **Chapter 4,** Desensitization strategies) could offer a solution, the Eurotransplant Acceptable Mismatch (AM) program (33) represents a valid alternative for patients in Austria, Belgium, Croatia, Germany, Hungary, Luxembourg, Slovenia and The Netherlands. This program fully prioritizes the allocation of compatible donor kidneys to highly sensitized patients (>85% cPRA), to increase their chances for transplantation, focusing on finding acceptable matches rather than to prohibit matches. It is an alternative approach to the sliding scale programs of the US and Spain mentioned previously. The main advantage of the AM over prioritization schemes is that it entails better matching and thus may lead to better long-term outcomes because of less susceptibility to primary alloimmune activation. Unfortunately, it does not seem to increase access to transplantation for those very highly sensitized patients (>99% cPRA).
Donor kidney offers (<65 years) are first allocated in the AM program, and only if no match is found in this program, are the kidneys allocated to the standard Eurotransplant Kidney Allocation System (ETKAS). The first priority is therefore checking immunological compatibility for the most highly sensitized patients, without considering other factors. In the AM program, the HLA mismatches that most likely will not result in a positive crossmatch are defined, based on detailed evaluation of the HLA antigens to which the patient has not yet reacted, and therefore might be acceptable for the patient. This detailed evaluation also encompasses computer algorithms such as HLAMatchmaker which helps in defining acceptable HLA or epitope mismatches (35, 36). Only patients who have been included on the standard Eurotransplant (ETKAS) waiting list for at least 2 years are eligible, and their cPRA% should be ≥85%. After the antibodies detected with CDC, only antibodies identified using solid phase assays are considered for the evaluation of the cPRA%, if they can be explained by previous immunizing events, e.g., HLA mismatches with previous donor(s) or a specific sensitization of the recipient such as HLA antigens of their partner or children in women. Not only are the classic HLA loci (HLA-A, B and -DR) considered, but also HLA-C and HLA-DQ. Access to the AM program is strictly controlled by the Eurotransplant Reference Laboratory (ETRL) in Leiden, The Netherlands, which reviews all relevant patient-level data before enrolling patients. Not all patients with a cPRA >85% are registered in the AM program, for several reasons, including the strict criteria for inclusion (**Figures 5 and 6**).
By fully prioritizing patients who are very highly sensitized by well-defined HLA antibodies, the Eurotransplant AM program clearly increases the chances for transplantation for this group of patients. The number of actively waiting patients included in the AM program has remained relatively constant over the past decade, as well as the numbers of patients transplanted within this program (**Figures 7 and 8**). A considerable number of patients have already been transplanted within the AM program (33), both first and repeat transplantations. Waiting times for transplantations in the AM program (thus of very highly sensitized patients) are significantly shorter than seen in similarly sensitized patients (cPRA >85%) not included in this program (33), illustrating that the AM program fulfills its primary goal, to increase access to transplantation for the most difficult to transplant patients.
In addition to benefits in terms of access to transplantation, there are also other very important messages we gain from detailed evaluation of the AM program. Kidney

transplant failure is significantly lower in the highly sensitized patients included in the AM program, compared with highly sensitized patients not included in the AM program. Furthermore, death-censored graft survival rate is similar to the rate in non-sensitized patients, and better than seen in mildly sensitized patients (33); and is related to a lower chance of rejection in the highly sensitized patients included in the AM program (34). Although at first sight counterintuitive, this important finding, that the most highly sensitized patients have the lowest risk of rejection, clearly illustrates that better risk stratification, thorough immunological evaluation (as in the AM program), and avoidance of HLA-DSA is highly beneficial for outcomes after kidney transplantation. This message is important beyond the implications for highly sensitized patients and makes the case for better (molecular) matching for general kidney transplants as a means to further decrease the risk of graft failure.

Although the AM program is highly successful (33) in terms of access to transplantation and outcomes after transplantation, a subset of patients enrolled in the AM program remain on the transplant waiting list because no compatible donors are available in the Eurotransplant donor population. This is exemplified by the discrepancy between the number of patients waiting in the AM program and the number of patients effectively transplanted each year (**Figure 8**), and represents the population of cPRA 99–100% mentioned previously. The patients remaining without transplants are mainly those with a rare HLA type compared with the HLA types of the actual donor population. Part of the population, therefore, remains waiting for a transplant, even in the AM program, with very limited chance of finding a suitable (HLA-DSA negative) donor. For this, the EUROSTAM project was initiated, which intends to expand the Eurotransplant AM program to a Europe-wide acceptable mismatch program (37). Simulations suggest that one in four of the highly sensitized patients who have been waiting a long time for a transplant (in total >700 patients identified), registered at each partner organization, have increased chances of transplant in a different European donor pool. Although the simulation exercises make a strong case for kidney sharing between European countries for selected patients, further practical and logistical work is needed before this Europe-wide AM program is implemented clinically (37).

6 Recommendations

Risk Stratification

- To define the humoral risk in kidney transplantation, the use of the ENGAGE 5 strata system is recommended

Organ Allocation

- Access to the donor pool should be increased through greater use of:
    - Sliding scale priority score schemes based on cPRA values (Chapters 1 and 3)
- Prioritization policies should be linked across countries for equity of access
- The Eurotransplant Acceptable Mismatch program should be expanded to other European countries to improve donor/recipient matching
- All kidney sharing schemes should develop calculators to help assess the probability of an organ match

- Therapeutic options should be reconsidered if there are no organ offers for a patient in a kidney sharing scheme

Areas for Further Research

- Work to develop schemes to help patients with very high cPRA who may not be transplanted in kidney paired donations or under deceased donor priority schemes should continue
- A further need for evidence-based information is in the role of induction immunosuppression in relation to sensitization and its role in long-term graft and patient outcomes
- Better risk stratification, thorough immunological evaluation and avoidance of HLA-DSA should be used to improve outcomes after kidney transplantation

Abbreviations

AM, acceptable mismatch; CDC, complement-dependent cytotoxicity; cPRA, calculated percentage of positive panel donors in an antibody screening assay; DSA, donor-specific antibodies; ENGAGE, EuropeaN Guidelines for the mAnagement of Graft rEcipients (ENGAGE) working group; ETKAS, Eurotransplant Kidney Allocation System; FCM, flow cytometric crossmatch; HLA, human leukocyte antigen; KSS, kidney sharing scheme; MFI, mean fluorescence intensity; SAB, single antigen bead

Author Contributions

The members of WS06 of ESOT provided input and critical review of this chapter: Frans Claas, Lucrezia Furian, Siân Griffin, Christophe Legendre, Liset Pengel.

Conflict of Interest

OB: Research funding from Chiesa and has served as an advisor for Hansa Biopharma
MN: No conflicts of interest with regard to this manuscript.
NM: Honoraria from Hansa, Chiesi, Novartis and Takeda.

FIGURE 1. Humoral risk stratification of kidney transplant candidates (adapted from reference 19)

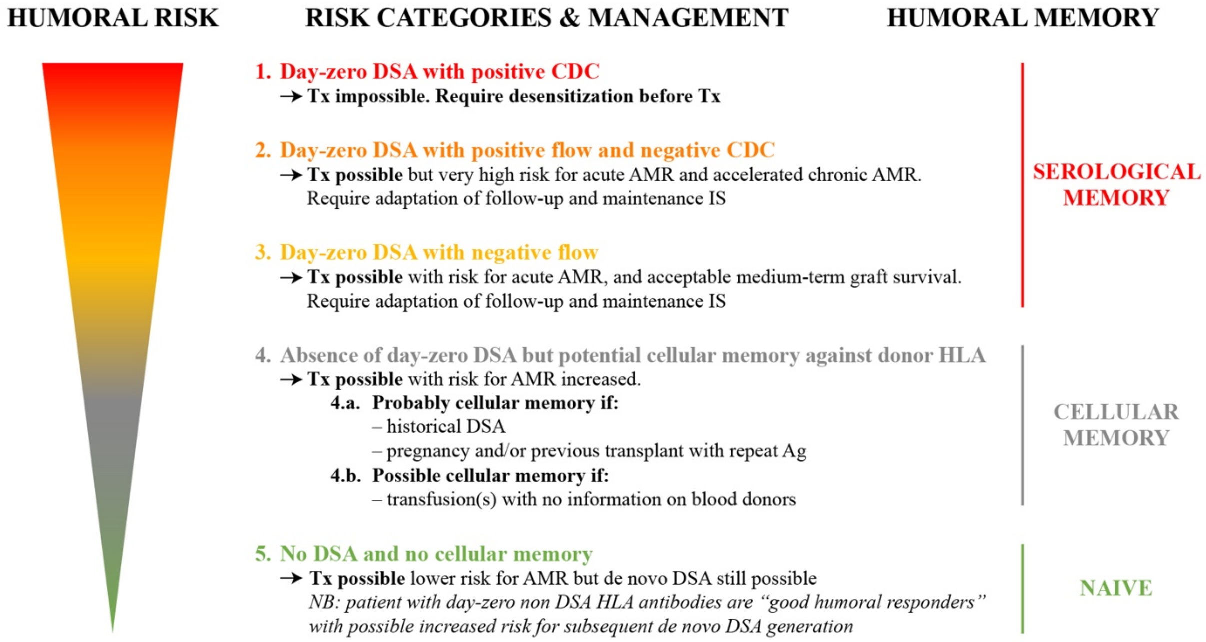

AMR, antibody-mediated rejection; CDC, complement-dependent cytotoxicity; DSA, donor-specific antibodies; HLA, human leukocyte antigen; IS, immunosuppression; Tx, transplant

FIGURE 2. Time on the wait list and percentage of patients receiving a kidney transplant relative to patient cPRA in the priority program for highly sensitized kidney transplant patients in Spain. Image reproduced with thanks and with permission from the Spanish priority allocation programme (PATHI) from the Spanish National Transplant Organization ((www-ONT.es).

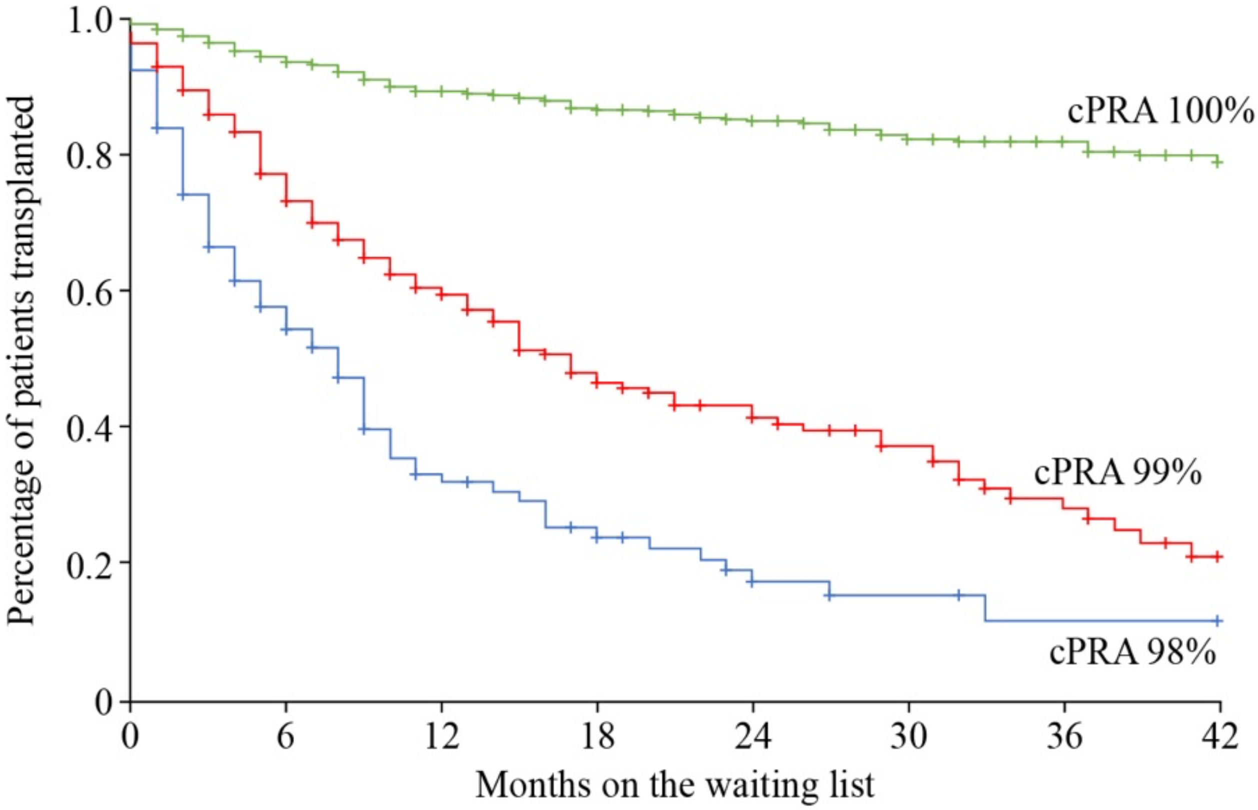

cPRA, calculated percentage of actual organ donors who express one or more unacceptable antigens

FIGURE 3. UK figures for the chance of a transplant by blood group, figures from 2012 onwards. National Health Service Organ Donation and Transplantation Clinical website (https://www.odt.nhs.uk)

2012 onwards

Recipient ABO

Donor ABO	O	A	B	AB
O	95/307 (31%)	81/144 (56%)	21/43 (49%)	6/12 (50%)
A	106/429 (25%)	56/192 (29%)	27/57 (47%)	2/14 (14%)
B	32/107 (30%)	21/54 (39%)	13/44 (30%)	1/6 (17%)
AB	4/17 (24%)	8/16 (50%)	3/14 (21%)	0/5 (0%)

FIGURE 4. Correlation of the chance of a transplant relative to the number of matching runs (UK figures from National Health Service Organ Donation and Transplantation Clinical website: https://www.odt.nhs.uk)

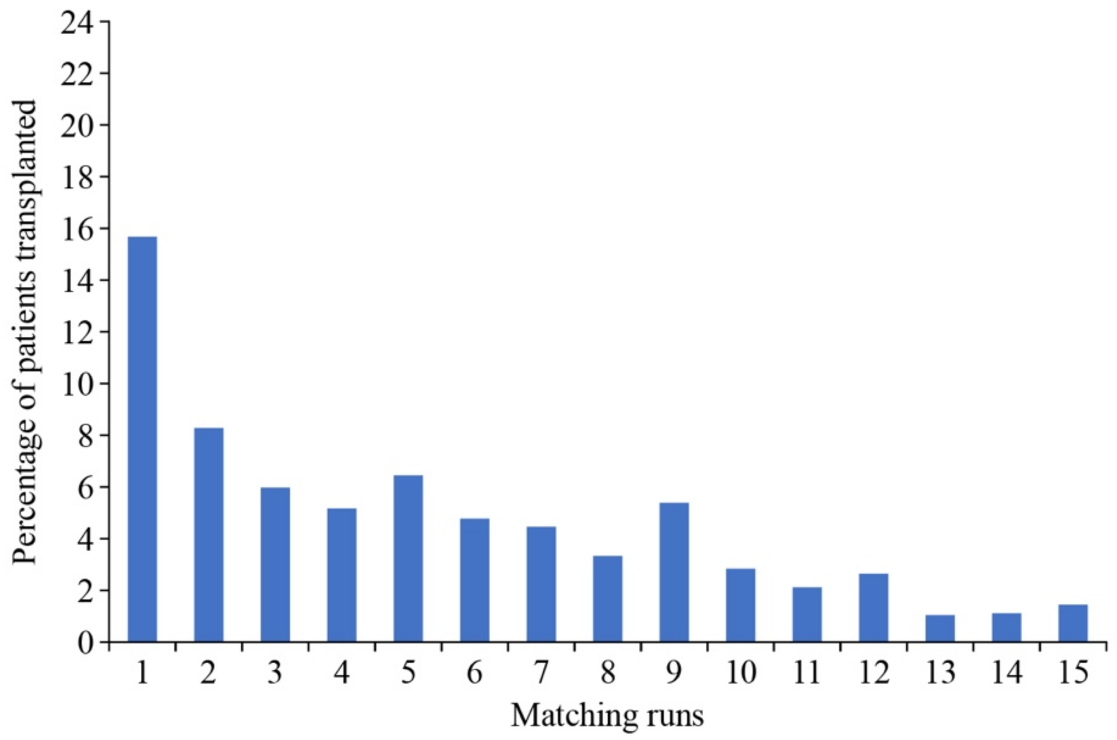

FIGURE 5. Relative numbers of patients in the Eurotransplant and Acceptable Mismatch (AM) programs (image reproduced with permission from Eurotransplant, www.eurotransplant.org. https://statistics.eurotransplant.org; accessed May 2021)

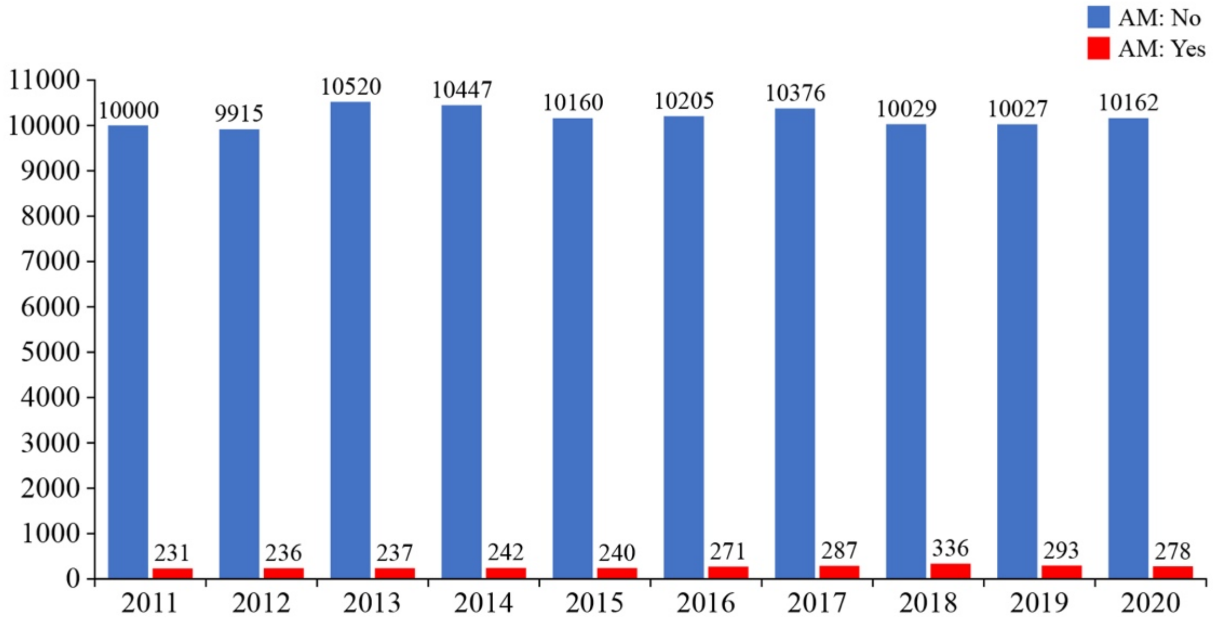

FIGURE 6: Relative numbers of highly sensitized patients in the Eurotransplant and Acceptable Mismatch (AM) programs (image reproduced with permission from Eurotransplant, www.eurotransplant.org. https://statistics.eurotransplant.org; accessed May 2021)

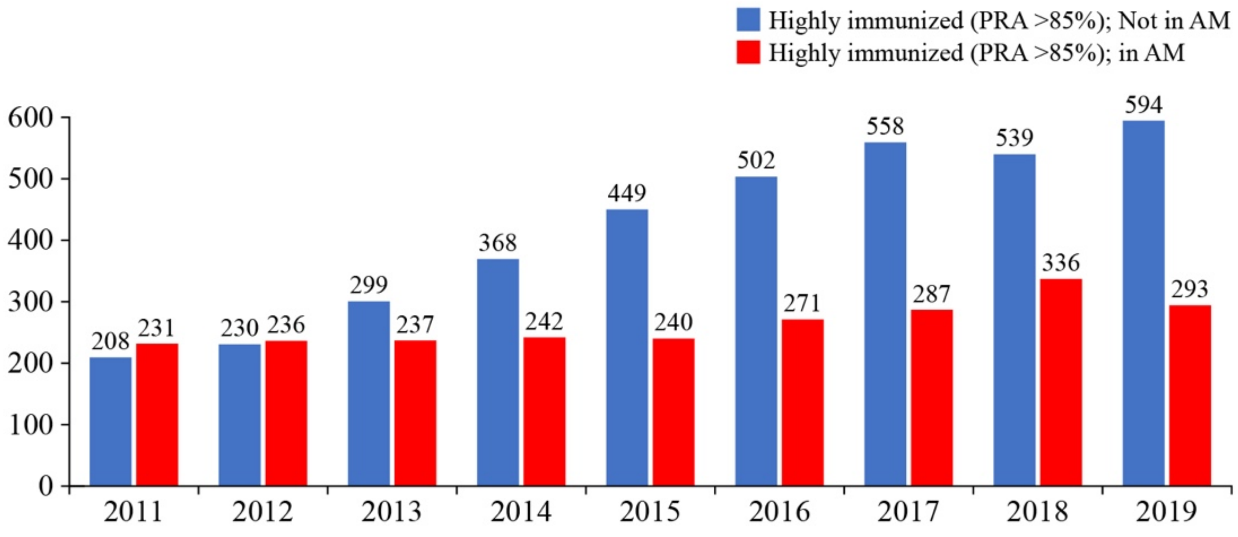

PRA, percentage of positive panel donors in an antibody screening assay

FIGURE 7. Relative numbers of kidney transplantations achieved by Eurotransplant and by the Acceptable Mismatch (AM) program (image reproduced with permission from Eurotransplant, www.eurotransplant.org. https://statistics.eurotransplant.org; accessed May 2021)

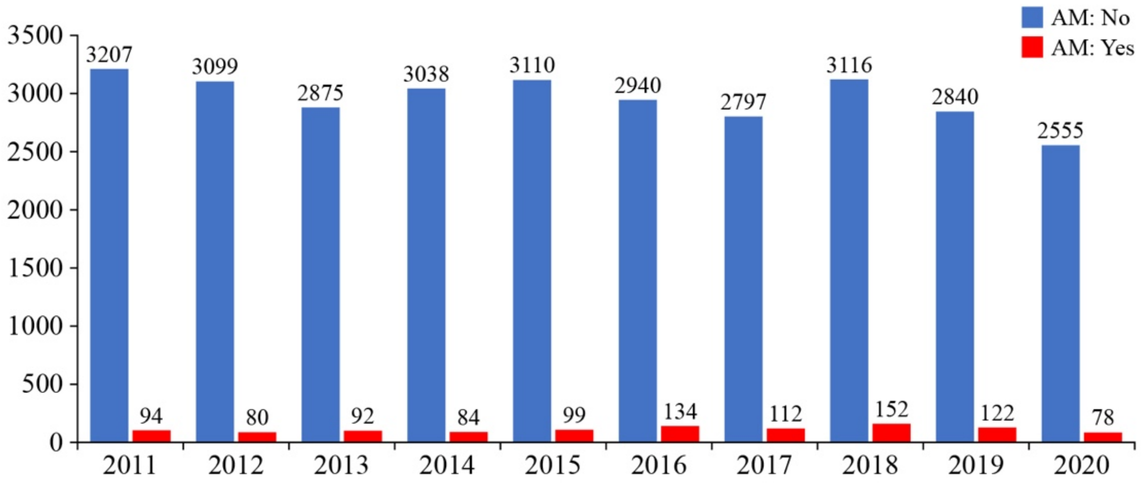

FIGURE 8. Relative number of kidney transplantations versus those remaining on the wait list within the Acceptable Mismatch program (image reproduced with permission from Eurotransplant, www.eurotransplant.org. https://statistics.eurotransplant.org; accessed May 2021)

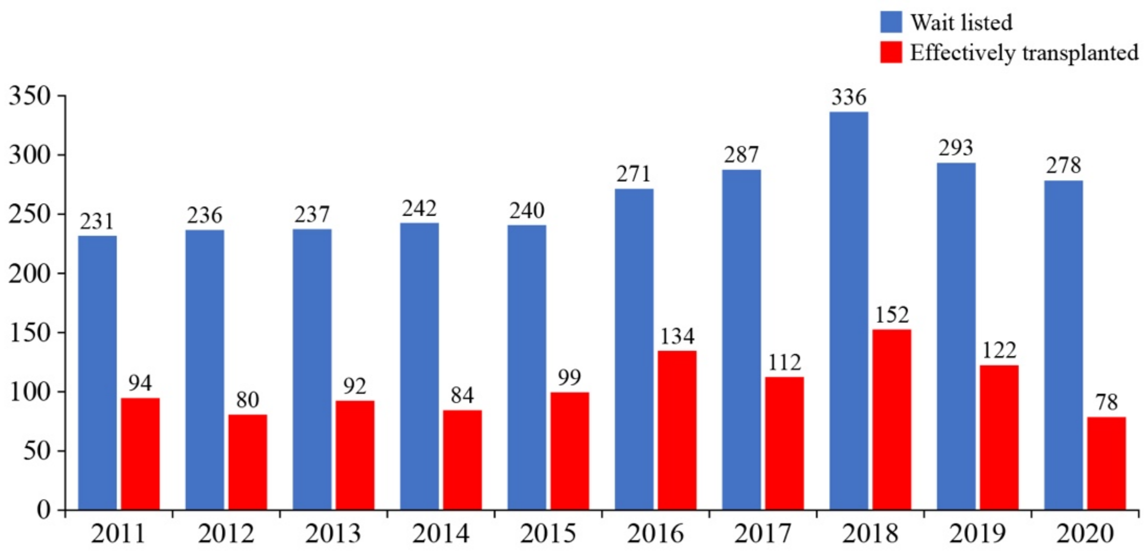

7 References

1. Kissmeyer-Nielsen F, Olsen S, Petersen V, Fjeldborg O. Hyperacute rejection of kidney allografts, associated with pre-existing humoral antibodies against donor cells. Lancet (1966) 24(2(7465)):662-5. doi: 10.1016/s0140-6736(66)92829-7.
2. Nadazdin O, Boskovic S, Murakami T, Tocco G, Smith RN, Colvin RB, et al. Host alloreactive memory T cells influence tolerance to kidney allografts in nonhuman primates. Sci Transl Med (2011) 3:86ra51. doi: 10.1126/scitranslmed.3002093.
3. Heeger PS, Greenspan NS, Kuhlenschmidt S, C Dejelo, D E Hricik, J A Schulak, et al. Pretransplant frequency of donor-specific, IFNgamma-producing lymphocytes is a manifestation of immunologic memory and correlates with the risk of posttransplant rejection episodes. J Immunol (1999) 163:2267-75.
4. Bestard O, Crespo E, Stein M, M Lúcia, D L Roelen, Y J de Vaal, et al. Cross-validation of IFN-gamma Elispot assay for measuring alloreactive memory/effector T cell responses in renal transplant recipients. Am J Transplant (2013) 13:1880-90. doi: 10.1111/ajt.12285.
5. Doxiadis II, Roelen D, Claas FH. Mature wines are better: CDC as the leading method to define highly sensitized patients. Curr Opin Organ Transplant (2010) 15:716-9. doi: 10.1097/MOT.0b013e3283402beb.
6. Couzi L, Araujo C, Guidicelli G, Thomas Bachelet, Karine Moreau, Delphine Morel, et al. Interpretation of positive flow cytometric crossmatch in the era of the single-antigen bead assay. Transplantation (2011) 91:527. doi: 10.1097/TP.0b013e31820794bb.
7. Schinstock CA, Gandhi MJ, Stegall MD. Interpreting anti-HLA antibody testing data: a practical guide for physicians. Transplantation (2016) 100:1619. doi: 10.1097/TP.0000000000001203.
8. Guidicelli G, Guerville F, Lepreux S, Wiebe C, Thaunat O, Dubois V, et al. Non-complement-binding de novo donor-specific anti-HLA antibodies and kidney allograft survival. J Am Soc Nephrol (2016) 27:615. doi: 10.1681/ASN.2014040326.
9. Hirohashi T, Uehara S, Chase CM, DellaPelle P, Madsen JC, Russell PS, et al. Complement independent antibody-mediated endarteritis and transplant arteriopathy in mice. Am J Transplant (2010) 10:510. doi: 10.1111/j.1600-6143.2009.02958.x.
10. Pouliquen E, Koenig A, Chen CC, Sicard A, Rabeyrin M, Morelon E, et al. Recent advances in renal transplantation: antibody-mediated rejection takes center stage. F1000Prime Rep (2015) 7:51. doi: 10.12703/P7-51.
11. Tao MH, Smith RI, Morrison SL. Structural features of human immunoglobulin G that determine isotype-specific differences in complement activation. J Exp Med (1993) 178:661. doi: 10.1084/jem.178.2.661.
12. Woof JM, Burton DR. Human antibody-Fc receptor interactions illuminated by crystal structures. Nat Rev Immunol (2004) 4:89. doi: 10.1038/nri1266.
13. Taylor CJ, Kosmoliaptsis V, Martin J, Knighton G, Mallon D, Bradley JA, et al. Technical limitations of the C1q single-antigen bead assay to detect complement binding HLA-specific antibodies. Transplantation (2017) 101:1206-14. doi: 10.1097/TP.0000000000001270.

14. Zachary AA, Lucas DP, Detrick B, Leffell MS. Naturally occurring interference in Luminex assays for HLA-specific antibodies: characteristics and resolution. Hum Immunol (2009) 70:496-501. doi: 10.1016/j.humimm.2009.04.001.
15. Roelen DL, Doxiadis II, Claas FH. Detection and clinical relevance of donor specific HLA antibodies: a matter of debate. Transpl Int (2012) 25:604-10. doi: 10.1111/j.1432-2277.2012.01491.x.
16. Schinstock CA, Gandhi M, Cheungpasitporn W, Mitema D, Prieto M, Dean P, et al. Kidney transplant with low levels of DSA or low positive B-flow crossmatch: an underappreciated option for highly sensitized transplant candidates. Transplantation (2017) 101:2429. doi: 10.1097/TP.0000000000001619.
17. Bachelet T, Visentin J, Guidicelli G, Merville P, Couzi L, Taupin JL. Anti-HLA donor-specific antibodies are not created equally. Don't forget the flow…. Transpl Int (2016) 29:508. doi: 10.1111/tri.12745.
18. Meneghini M, Melilli E, Martorell J, Revuelta I, Rigol-Monzó E, Manonelles A, Montero N, et al. Combining sensitive crossmatch assays with donor/recipient human leukocyte antigen eplet matching predicts living-donor kidney transplant outcome. Kidney Int Rep (2018) 3:926. doi: 10.1016/j.ekir.2018.03.015.
19. Bestard O, Couzi L, Crespo M, Kessaris N, Thaunat O. Stratifying the humoral risk of candidates to a solid organ transplantation: a proposal of the ENGAGE working group. Transpl Int (2021) 34:1005-18. doi:10.1111/tri.13874.
20. Sapir-Pichhadze R, Tinckam KJ, Laupacis A, Logan AG, Beyene J, Kim SJ. Immune sensitization and mortality in wait-listed kidney transplant candidates. J Am Soc Nephrol (2016) 27:570-8. doi: 10.1681/ASN.2014090894.
21. Bostock IC, Alberú J, Arvizu A, Hernández-Mendez EA, De-Santiago A, González-Tableros N, et al. Probability of deceased donor kidney transplantation based on % PRA. Transpl Immunol (2013) 28:154-8. doi: 10.1016/j.trim.2013.05.002.
22. Israni AK, Salkowski N, Gustafson S, Snyder JJ, Friedewald JJ, Formica RN, et al. New national allocation policy for deceased donor kidneys in the United States and possible effect on patient outcomes. J Am Soc Nephrol (2014) 25:1842-8. doi: 10.1681/ASN.2013070784.
23. Colovai AI, Ajaimy M, Kamal LG, Masiakos P, Chan S, Savchik C, et al. Increased access to transplantation of highly sensitized patients under the new kidney allocation system. A single center experience. Hum Immunol (2017) 78:257-62. doi: 10.1016/j.humimm.2016.12.003.
24. Hickey MJ, Zheng Y, Valenzuela N, Zhang Q, Krystal C, Lum E, et al. New priorities: analysis of the new kidney allocation system on UCLA patients transplanted from the deceased donor waitlist. Hum Immunol (2017) 78:41-8. doi: 10.1016/j.humimm.2016.10.020.
25. Stewart DE, Kucheryavaya AY, Klassen DK, Turgeon NA, Formica RN, Aeder MI. Changes in deceased donor kidney transplantation one year after KAS implementation. Am J Transplant (2016) 16:1834-47. doi: 10.1111/ajt.13770.
26. Stewart DE, Klassen DK. Kidney transplants from HLA-incompatible live donors and survival. N Engl J Med (2016) 375:287-8. doi: 10.1056/NEJMc1604523.

27. Samoylova ML, Shaw BI, Irish W, McElroy LM, Connor AA, Barbas AS, et al. Decreased graft loss following implementation of the kidney allocation score (KAS). Am J Surg (2020) 220:1278-83. doi: 10.1016/j.amjsurg.2020.06.061.
28. Valentin MO, Ruiz JC, Vega R, Martín C, Matesanz R; working group PATHI. Implementation of a national priority allocation system for hypersensitized patients in Spain, based on virtual crossmatch: Initial results. Transplant Proc (2016) 48:2871-5. doi: 10.1016/j.transproceed.2016.09.024.
29. Massie AB, Luo X, Lonze BE, Desai NM, Bingaman AW, Cooper M, et al. Early changes in kidney distribution under the new allocation system. J Am Soc Nephrol (2016) 27:2495-501. doi: 10.1681/ASN.2015080934.
30. Houp JA, Schillinger KP, Eckstein AJ, Vega RM, Desai NM, Lonze BE, et al. Casting a smaller net into a bigger donor pool: a single center's experience with the new kidney allocation system. Hum Immunol (2017) 78:49-53. doi: 10.1016/j.humimm.2016.11.004.
31. Kransdorf EP, Pando MJ. Calculated panel reactive antibody with decimals: a refined metric of access to transplantation for highly sensitized candidates. Hum Immunol (2017) 78:252-6. doi: 10.1016/j.humimm.2016.12.009.
32. Jackson KR, Chen J, Kraus E, Desai N, Segev DL, Alachkar N. Outcomes of cPRA 100% deceased donor kidney transplant recipients under the new Kidney Allocation System: A single-center cohort study. Am J Transplant (2020) 20:2890-8. doi: 10.1111/ajt.15956.
33. Heidt S, Haasnoot GW, van Rood JJ, Witvliet MD, Claas FHJ. Kidney allocation based on proven acceptable antigens results in superior graft survival in highly sensitized patients. Kidney Int (2018) 93:491-500. doi: 10.1016/j.kint.2017.07.018.
34. Heidt S, Haasnoot GW, Witvliet MD, van der Linden-van Oevelen MJH, Kamburova EG, Wisse BW, et al. Allocation to highly sensitized patients based on acceptable mismatches results in low rejection rates comparable to nonsensitized patients. Am J Transplant (2019) 19:2926-33. doi: 10.1111/ajt.15486.
35. Claas FH, Doxiadis II. Management of the highly sensitized patient. Curr Opin Immunol (2009) 21:569-72. doi: 10.1016/j.coi.2009.07.010.
36. Chen M, Zoet Y, Roelen D, Martorell J, Middleton D, Slavcev A, et al. Towards uniformity in the definition of acceptable mismatches for highly sensitized patients. HLA (2019) 94:147-53. doi: 10.1111/tan.13607.
37. Mumford L, Fuggle SV, Martorell J, Slavcev A, Iniotaki A, Haasnoot GW, et al. A Europe wide acceptable mismatch program will enable transplantation of long waiting highly sensitised patients with a compatible donor. Transpl Immunol (2020) 64:101354. doi: 10.1016/j.trim.2020.101354.

Chapter 4: Desensitization Strategies in Kidney Transplantation

Christophe Legendre*, on behalf of Workstream 06 of the Transplantation Learning Journey of the European Society of Organ Transplantation

Department of Nephrology and Adult Kidney Transplantation, Hôpital Necker and Université de Paris, Paris, France

*** Correspondence:** *Christophe Legendre, Christophe.legendre@aphp.fr*

Keywords: desensitization, kidney transplantation, immunization, donor-specific antibodies

Word count (body text only): 2393
Number of figures: 3
Number of tables: 0

Abstract

As the number of HLA-sensitized or even highly sensitized patients grows on transplant waiting lists, the need for desensitization strategies becomes ever more important. In this short review, first we describe the current treatments that are or have been used in order to transplant these patients, and secondly, we examine the best ways to evaluate the results from using these strategies. It is currently possible to remove or block donor-specific antibodies, but the problem of rebound with a high incidence of antibody-mediated rejection is still unsolved.

1 Introduction

The percentage of patients registered on kidney transplant lists who are anti-HLA immunized or even highly immunized is currently increasing in the majority of transplant teams throughout the world (1). For example, in our group (Necker Hospital, Paris, France), 62% of listed patients are anti-HLA immunized and 33% are highly immunized – both these groups of patients spend significantly more time waiting for a suitable kidney, and are therefore on dialysis until an organ is available and can be transplanted. Time on dialysis is a bad prognostic factor in the long-term, mainly because of cardiovascular complications. Therefore, there is a need to improve the transplantability of these patients – a fact especially true for patients waiting for retransplantations.

Children needing dialysis fare more poorly than adults, with treatment impacting growth, and development, (2) as well as psychosocial functioning, with children on dialysis more likely to report depression/anxiety, issues with self-esteem, and behavioural problems than their peers who do not require dialysis (3). A long wait for a donor organ is particularly traumatic for children, and to counter this, they are often transplanted quickly but not necessarily with a very well-matched kidney. This can cause problems later in life for these recipients, as children who receive a donor organ are highly likely to require at least one retransplantation during their life-time and retransplant patients are often highly

sensitized and difficult to match with donor organs. In addition, pediatric kidney transplant recipients have a high incidence of poor adherence to medication (estimates of non-adherence range from 30 to 70% among pediatric patients) (4), which increases the risk of graft failure and mortality.

2 Strategies to Improve Patient Transplantability

- Strategy 1 is to wait for a well-matched donor kidney, but the anticipated price to pay is a long time spent on the waiting list!
- Strategy 2 relies on acceptable mismatch programs (5), which look for a donor with or without low titers of donor-specific antibodies (DSA); the donor kidney can be coupled to kidney-paired exchange programs (6), or not. This strategy is providing excellent long-term results and increases the number of patients transplanted. However, it is not the solution for all patients.
- Strategy 3 is desensitization and consists of interfering with DSAs either before transplantation (when a potential living donor has been identified) (7), immediately before transplantation in order to facilitate the crossmatch (8) or just after transplantation, in the case of transplantation with a kidney from a deceased donor (9). This chapter will focus mainly on the third strategy.

3 Desensitization in Kidney Transplantation: What are the Current Options?

There are several ways to desensitize HLA-immunized patients, which involve utilizing specific drugs or monoclonal antibodies. Historically, polyvalent intravenous immunoglobulins (IVIgs) were used alone (10). In a randomized trial (11), it was shown that IVIgs alone allowed more patients to be transplanted, but the overall benefit was still quite limited. IVIgs exert their effects by several complex modes of action, including modulation of antibody action, anti-complement effects and anti-cytokine effects (12).

It is relatively simple to decrease the global level of IVIgs through plasma exchange (PE) or by immune-adsorption - an equivalent method. Both methods have drawbacks including the frequent need for a central catheter (with the incumbent risk of infection this creates) and modification of coagulation factors, which increases the risk of bleeding. The number of PEs necessary to lower the IgG level is about five and the gain of increasing the number of PEs beyond that is small (7).

Rituximab, the anti-CD20 monoclonal antibody that is expressed on pre-plasmocyte precursors can be used to desensitize patients prior to transplantation. This drug aims to decrease the rebound effect linked to decreased levels of immunoglobulins in the plasma. Efficacy is monitored using the expression of CD19 on B cells.

Currently, the two methods used to desensitize patients are either a combination of anti-CD20 antibodies and high-dose IVIgs (2 g/kg over 2 to 4 days) (13), probably useful for immunized patients but less so for those highly sensitized, or a combination of 3 to 5 sessions of PE followed after each session by an infusion of low-dose IVIgs (0.1 g/kg) to avoid the rebound following a decreased level of circulating IVIgs (7). New anti-CD20 monoclonal antibodies (such as ocrelizumab or obinutuzumab) may be more efficient, as well as anti-CD19 antibodies.

It is possible to decrease the synthesis of proteins (DSAs) using proteasome inhibitors such as the first-generation drug, bortezomib (14). This drug was tested in a study with such a complex design (including the testing of many drugs as well as bortezomib) that it

is difficult to clearly see its role in desensitization (15)! Second generation drugs such as carfilzomib or ixazomib may be more efficient. These drugs require administration of steroids at the same time, and their main problem is their neurological toxicity that might not be reversible (15).

A logical approach to desensitization is to block the activity of complement in order to decrease the effect of antibodies such as DSAs. The anti-C5 monoclonal antibody, eculizumab, was the first to be tested in this indication. In a non-randomized study using eculizumab in addition to desensitization and historical controls, Stegall et al (16) showed a very significant decrease in the incidence of antibody-mediated rejection (ABMR) and transplant glomerulopathy on screening biopsies. Unfortunately, increasing the number of biopsies led to an equivalent incidence of transplant glomerulopathy in this study. A randomized study was designed for patients receiving a kidney from a living donor and comparing the use of eculizumab for 3 months post-transplantation with a control group who received desensitization (17). Unfortunately, the results were rather disappointing or at least, difficult to interpret, with no significant difference found between the two groups. One explanation of these results is the difficulties in defining ABMR (with results varying depending on whether biopsies were graded by local or central pathologists) and probably more importantly, the use of anti-C5 in patients with DSAs not fixing the complement (18). It is likely that this treatment could be efficient in certain circumstances, but it remains to be demonstrated. In contrast, in a study in patients being transplanted with an organ from a deceased donor, it was possible to get a low incidence of ABMR (around 10% during the first 3 months) (19). However, there were no controls in this study, so the overall results are not clear-cut, but it remains a logical approach that may be used in selected groups of patients. Other complement blockers (such as a C1-inhibitor) are the subject of current clinical trials (20).

Another approach is the use of a cysteine protease (IG endopeptidase, Ides, Imlifidase and Idefirix®). Imlifidase is currently the only approved therapy for use in the EU for desensitization treatment of highly sensitized adult kidney transplant patients with a positive crossmatch against an available deceased donor. (https://www.ema.europa.eu/en/medicines/human/EPAR/idefirix). It cleaves all IgGs, both intra- and extravascularly, without regard to their specificity, with an immediate action that lasts around 5 to 7 days; this drug cannot be re-dosed due to immunogenicity (21) (**Figure 1**). Imlifidase has been used in HLA incompatible hyper-immunized patients with good and safe results and at 3 years, crossmatch positive patients who were converted to negative with imlifidase to enable transplantation had rebound effect and ABMR with a frequency equivalent to other desensitization methods. Three years after imlifidase-enabled desensitization and transplantation, the death-censored allograft survival was 84%, patient survival 90%, and mean eGFR was 55 mL/min/1.73 m^2 (49 mL/min/1.73 m^2 for those with AMR and 61 ml/min/m^2 for those without AMR)(22). Ease of administration includes rapid infusion time over 15 minutes without the need of a central intravenous line; however, due to its unique mechanism of action, timing of co-administration with antibody-based therapies such as rabbit IgG, need to be taken into consideration (23).

An additional desensitization strategy is the manipulation of the cytokines involved in B cells action. In this indication, tocilizumab, an anti-IL6 receptor monoclonal antibody has been giving promising results in a randomized trial, used in addition to current

desensitization protocols (24). Antibodies to anti-IL6 are also undergoing study for this indication. Belimumab, an anti-BAFF monoclonal antibody, might be a useful adjunct to standard care immunosuppression in renal transplantation patients, as it shows no major increased risk of infection and potential beneficial effects on humoral alloimmunity (25) (**Figure 2**)

Finally, it would be logical to use a monoclonal antibody against plasmocytes (such as daratumumab), which gives promising results in non-human primate models and in a few patients (26).

4 How to Evaluate the Efficacy of Desensitization?

This is the most important issue, but not a simple one.

The main goal of desensitization is to allow patients who would otherwise not have been transplanted to receive a donor organ with an 'acceptable result'. In the literature, patient and graft survival have been used to show efficacy. For example, at the Johns Hopkins Hospital in Baltimore, USA, patient survival of desensitized recipients (transplanted with a living donor kidney) was statistically better than survival either in a group of non-listed patients receiving dialysis or in a group of listed patients, transplanted or not, but without desensitization (7). This provides outcomes on the efficacy of desensitization as well as on the 'quality of dialysis', but this result may not be generalizable.

Using data from several transplant centers in the USA, the same group showed that there was a negative correlation between number of graft losses and immunological risk (27). Graft survival at 5 years was highest in the reference group, then decreased in order to patients without DSAs, then to patients with DSAs but a negative flow crossmatch, to patients with DSAs but a positive flow crossmatch and finally to patients with DSAs and a positive CDC crossmatch.

More recently (28), it was shown that patients with a living donor and desensitization had a better graft outcome than patients either listed or not listed but in dialysis. These data outline very clearly that defining what is an 'acceptable transplantation' is very subjective and variable from one country to another.

The experience from the UK is very interesting (29). Survival of sensitized patients undergoing HLA-incompatible transplantation is comparable with those on dialysis awaiting a compatible organ, many of which are unlikely to receive a transplant. Choosing a direct HLAi transplant has no detrimental effect on survival, but offers no survival benefit, which is in contrast with similar patients studied in a North American multicenter cohort (27).

In Seoul, Republic of Korea, the average waiting time for an HLA-compatible deceased donor kidney transplant (DDKT) is long, >5 years, which impacts the relative benefit of each transplant option. In a study of outcomes, significantly better patient survival was seen in those undergoing HLAi living-donor kidney transplant (LDKT) compared with those remaining on the waiting list and compared with those on the waiting list or who had received an HLA-compatible DDKT. In addition, the HLAi LDKT group survival benefit was seen at all strengths of donor-specific antibodies, suggesting that HLAi LDKT is a good option for sensitized patients with kidney failure in countries with prolonged waiting times for DDKT, such as the Republic of Korea (30).

Finally, in our group (9), patients with DSAs at the time of transplantation (mean of 9421 mean fluorescence intensity (MFI)) and desensitized not before, but after transplantation, exhibited a graft survival of 78% at 7 years, which is not very different from patients transplanted without DSAs. An increased incidence of infections was unfortunately the price to pay.
Overall, there is no doubt that transplanting patients with DSAs negatively impacts the results of transplantation and there is a necessary balance between the benefits of transplantation and complications, especially infectious ones.
The results of desensitization also rely on a careful analysis of DSAs (Luminex SA®, MFI, acceptable threshold, dilution test etc.), flow cytometry crossmatch (channel shift, positivity or negativity) and also CDC crossmatch. There is a correlation between DSA titers and histological lesions from normal biopsies to clinically active humoral rejection. In most studies about desensitization, the frequency of acute antibody-mediated rejection is still around 30 to 40% during the first year. The transplant glomerulopathy (cg in the Banff classification) lesion may also be a prognostic factor. Finally, renal function and proteinuria are also good prognostic factors; however, as there are not many randomized trials in this group of patients, there is still discussion about this.
Even though the Luminex® test to detect DSAs is only semi-quantitative, there are some correlations between MFI and clinical events, immunological risk and final graft survival. Also, the correlation between level of MFI and positivity of crossmatches, either cellular or flow-cytometric, is far from perfect (**Figure 3**).

5 Conclusion

Desensitization is an option that will need to be used more and more often in organ transplantation for those patients who can not otherwise benefit from a transplant and need to remain on dialysis. Overall, there is no doubt that transplanting patients with DSAs negatively impacts the results of transplantation and there is a necessary balance between the benefits of transplantation and complications, especially infectious ones. Desensitization will also have to be considered in allocation policies as they are updated, because the number of patients who are immunized and highly immunized is growing in most countries. Cocktails of medications will be necessary to manage desensitization efficiently with an ‘acceptable’ safety profile.

6 Recommendations

Organ Allocation

- We recommend all countries and centers have an active policy of prioritizing highly sensitized patients for organ transplantation

Desensitization

- The most efficacious desensitization strategy is to start with rounds of plasma exchanges/immunoadsorption together with B-cell immunomodulation with IVIG or B-cell depletion with anti-CD20 monoclonal antibodies
- As yet to be defined protocols including proteasome inhibitors and other anti-plasmocyte antibodies with costimulation blockade, B-cell immunomodulation

targeting IL-6 as well as cleavage of IgG donor-specific antibodies with imlifidase are highly promising new strategies that deserve further investigation

Abbreviations

ABMR, antibody-mediated rejection; CDC, complement-dependent cytotoxicity; DDKT, deceased-donor kidney transplant; DSA, donor-specific antibodies; HLA, human leukocyte antigen; LDKT, living-donor kidney exchange; MFI, mean fluorescence intensity; PE, plasma exchange

Author Contributions

The members of WS06 of ESOT provided input and critical review of this chapter: Oriol Bestard, Frans Claas, Lucrezia Furian, Siân Griffin, Nizam Mamode, Maarten Naesens, Liset Pengel.

Conflict of Interest

The author declares that the research was conducted in the absence of any commercial or financial relationships that could be construed as a potential conflict of interest. The author has received travel grants and has been an invited speaker for Novartis, Atellas, Alexion, CSL Behring, and has consulted for Hansa Biopharma AB.

FIGURE 1. Donor-specific antibody levels over 6 months after a dose of imlifidase (From N Engl J Med, Jordan SC, Lorant T, Choi J, Kjellman C, Winstedt L, Bengtsson M, et al., Endopeptidase in highly sensitized patients undergoing transplantation, Volume

No. 377(5), Page No. 442-53. Copyright © (2021) Massachusetts Medical Society. Reprinted with permission from Massachusetts Medical Society.)

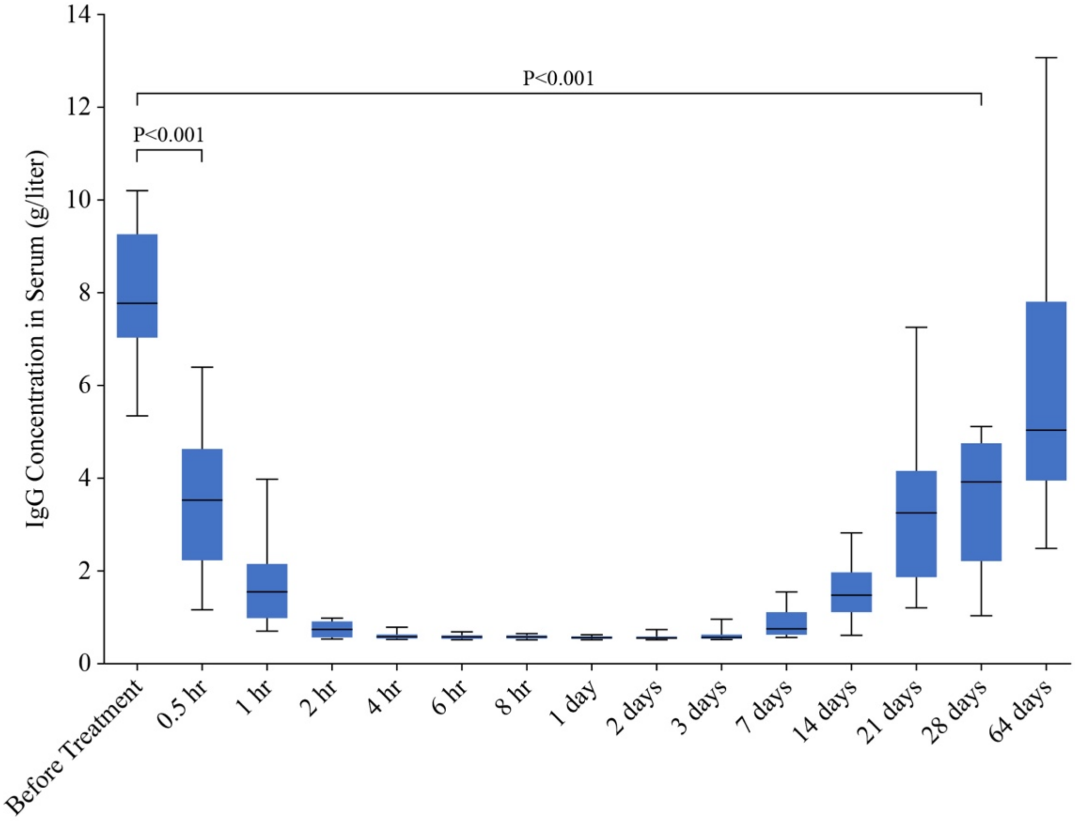

DSA, donor-specific antibody; MFI, mean fluorescence intensity

FIGURE 2. Representation of the mode of action of desensitizing drugs and monoclonal antibodies (31; Copyright 2021 Wiley, used with permission from Clatworthy MR. Targeting B Cells and Antibody in Transplantation. John Wiley and Sons)

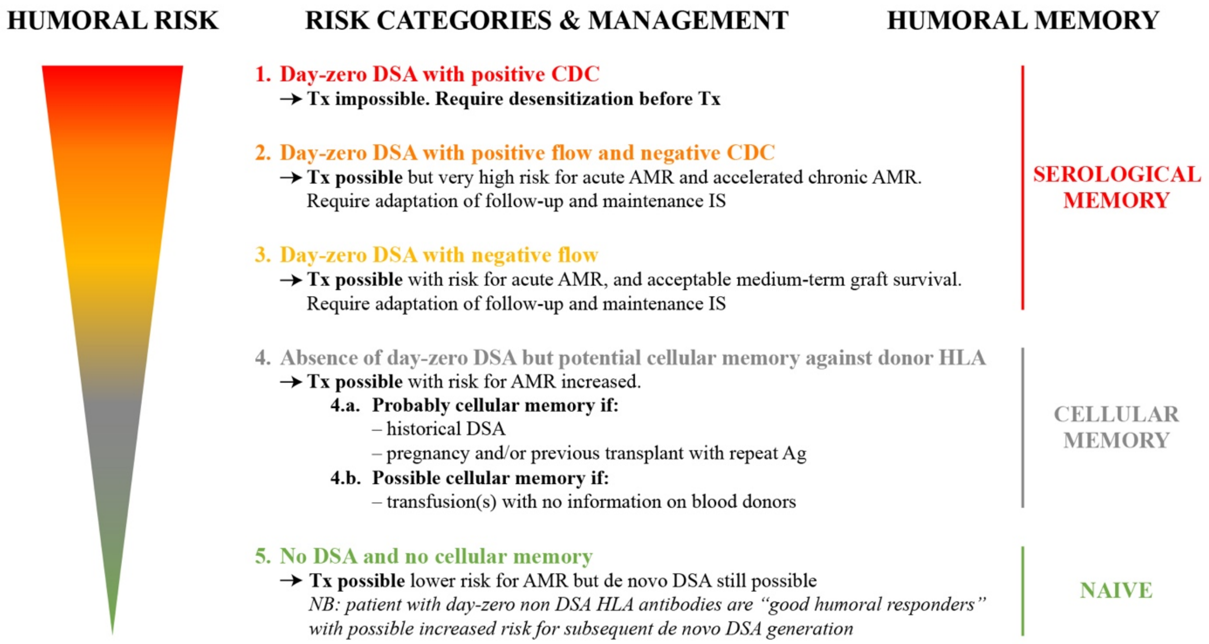

FIGURE 3. A schematic representation of the relationship between risk of rejection and the level of donor-specific antibodies, measured by mean fluorescence intensity. The positivity of either cytometry or complement-dependent cytotoxicity crossmatches does not occur at the same value of mean fluorescence intensity, making interpretation of the immunological risk complex at the very least

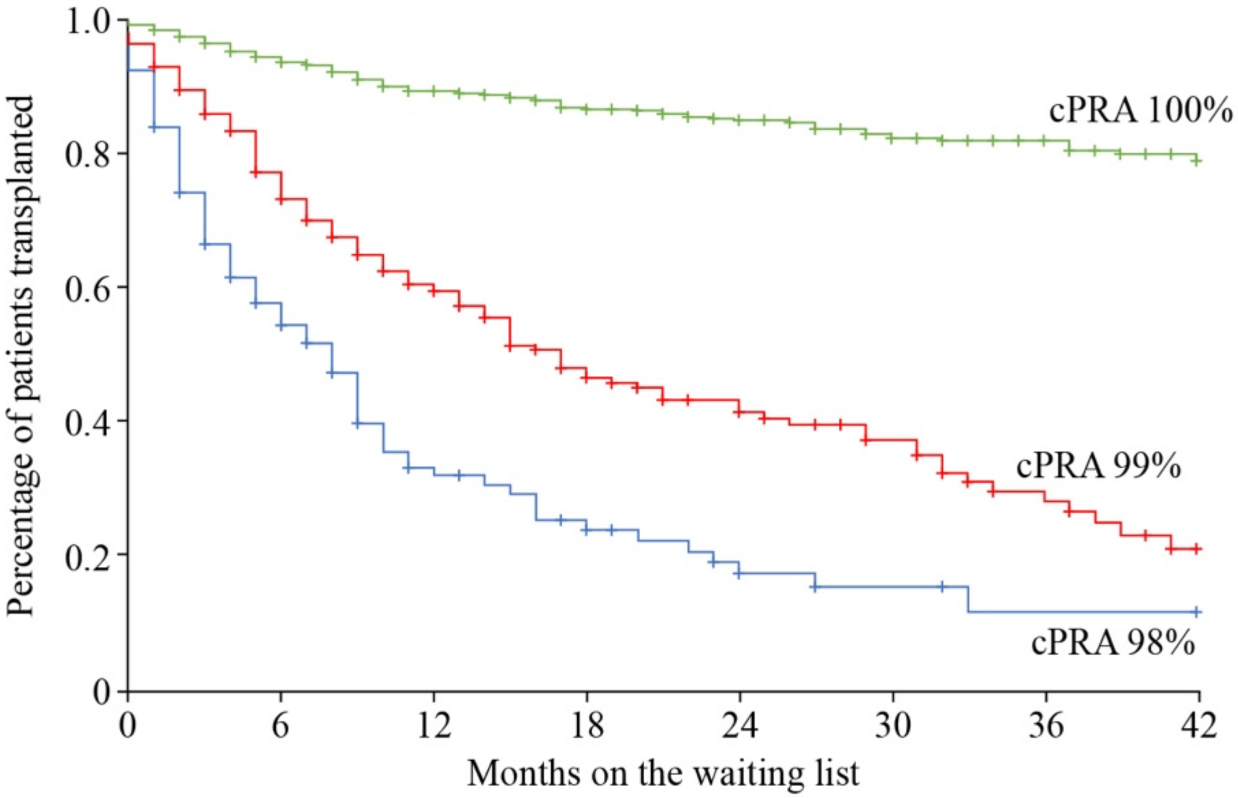

AMR, antibody-mediated rejection; CDC-XM, complement-dependent cytotoxicity crossmatch; Flow-XM, flow cytometric crossmatch; MFI, mean fluorescence intensity
The red dotted lines and question marks indicate that the thresholds for mean fluorescence intensity that define immunological risk are unknown

7 References

1. Tambur AR, Campbell P, Claas FH, Feng S, Gebel HM, Jackson AM, et al. Sensitization in Transplantation: Assessment of Risk (STAR) 2017 Working Group Meeting Report. Am J Transplant (2018) 18:1604-14. doi: 10.1111/ajt.14752.
2. Verghese PS. Pediatric kidney transplantation: a historical review. Pediatr Res (2017) 81(1-2):259-64. doi: 10.1038/pr.2016.207.
3. Clementi MA, Zimmerman CT. Psychosocial considerations and recommendations for care of pediatric patients on dialysis. Pediatr Nephrol (2020) 35:767-75. doi: 10.1007/s00467-019-04227-5.
4. Steinberg EA, Moss M, Buchanan CL, Goebel J. Adherence in pediatric kidney transplant recipients: solutions for the system. Pediatr Nephrol (2018) 33:361-72. doi: 10.1007/s00467-017-3637-0.
5. Mumford L, Fuggle SV, Martorell J, Slavcev A, Iniotaki A, Haasnoot GW, et al. A Europe acceptable mismatch program will enable transplantation of long waiting highly sensitized patients with a compatible donor. Transpl Immunol (2020) 64:101354. doi: 10.1016/j.trim.2020.101354.
6. Gentry SE, Montgomery RA, Segev DL. Kidney paired donation: fundamentals, limitations and expansions. Am J Kidney Dis (2011) 57:144-51. doi: 10.1053/j.ajkd.2010.10.005.
7. Montgomery RA, Lonze BE, King KE, Kraus ES, Kucirka LM, Locke JE, et al. Desensitization in HLA-incompatible kidney recipients and survival. N Engl J Med (2011) 305:318-26. doi: 10.1056/NEJMoa1012376.
8. Bartel G, Warhmann M, Regele H, Kikić Z, Fischer G, Druml W, et al. Peritransplant immunoadsorption for positive crossmatch deceased donor kidney transplantation. Am J Transplant (2010) 10:2033-42. doi: 10.1111/j.1600-6143.2010.03226.x.
9. Amrouche L, Aubert O, Suberbielle C, Rabant M, Van Huyen JD, Martinez F, et al. Long-term outcomes of kidney transplantation in patients with high levels of preformed DSA: the Necker high-risk transplant program. Transplantation (2017) 101:2440-8. doi: 10.1097/TP.0000000000001650.
10. Glotz D, Haymann JP, Sansonetti N, Francois A, Menoyo-Calonge V, Bariety J, et al. Suppression of HLA-specific alloantibodies by high-dose intravenous immunoglobulins (IVIg). A potential tool for transplantation of immunized patients. Transplantation (1993) 56:335-7. doi: 10.1097/00007890-199308000-00015.
11. Jordan SC, Tyan D, Stablein D, McIntosh M, Rose S, Vo A, et al. Evaluation of intravenous immunoglobulin as an agent to lower allosensitization and improve transplantation in highly sensitized adult patients with end-stage renal disease: report of the NIH IG02 trial. J Am Soc Nephrol (2004) 15:3256-62. doi: 10.1097/01.ASN.0000145878.92906.9F.
12. Tedla FM, Roche-Recinos A, Brar A. Intravenous immunoglobulin in kidney transplantation. Curr Opin Organ Transplant (2015) 20:630-7. doi: 10.1097/MOT.0000000000000250.
13. Vo AA, Lukovsky M, Toyoda M, Wang J, Reinsmoen NL, Lai CH, et al. Rituximab and intravenous immune globulin for desensitization during renal transplantation. N Engl J Med (2008) 359:241-51. doi: 10.1056/NEJMoa0707894.

14. Woodle ES, Tremblay S, Driscol J. Targeting plasma cells with proteasome inhibitor: principles from primates. Am J Soc Nephrol (2017) 28:1951-53. doi: 10.1681/ASN.2017040443.
15. Woodle ES, Shields AR, Ejaz NS, Sadaka B, Girnita A, Walsh RC, et al. Prospective iterative trial of proteasome inhibitor-based desensitization. Am J Transplant (2015) 15:101-18. doi: 10.1111/ajt.13050.
16. Stegall MD, Diwan T, Raghavaiah S, Cornell LD, Burns J, Dean PG, et al. Terminal complement inhibition decreases antibody-mediated rejection in sensitized renal transplant recipients. Am J Transplant (2011) 11:2405-13. doi: 10.1111/j.1600-6143.2011.03757.x.
17. Marks WH, Mamode N, Montgomery RA, Stegall MD, Ratner LE, Cornell LD, et al. Safety and efficacy of eculizumab in the prevention of antibody-mediated rejection in living-donor kidney transplant recipients requiring desensitization therapy: a randomized trial. Am J Transplant (2019) 19:2876-88. doi: 10.1111/ajt.15364.
18. Loupy A, Lefaucheur C, Vernerey D, Prugger C, Duong van Huyen JP, Mooney N, et al. Complement-binding anti-HLA antibodies and kidney-allograft survival. N Engl J Med (2013) 369:1215-26. doi: 10.1056/NEJMoa1302506.
19. Glotz D, Antoine C, Julia P, Pegaz-Fiornet B, Duboust A, Boudjeltia S, et al. Intravenous immunoglobulins and transplantation for patients with anti-HLA antibodies. Transpl Int (2004) 17:1-8. doi: 10.1007/s00147-003-0674-3.
20. Berger M, Lefaucheur C, Jordan SC. C1 esterase inhibitor in human solid organ transplantation. Transplantation (2019) 103:1763-75. doi: 10.1097/TP.0000000000002717.
21. Jordan SC, Lorant T, Choi J, Kjellman C, Winstedt L, Bengtsson M, et al. IgG Endopeptidase in highly sensitized patients undergoing transplantation. N Engl J Med (2017) 377:442-53. doi: 10.1056/NEJMoa1612567. Erratum in: N Engl J Med. 2017 Oct 26;377(17):1700.
22. Kjellman C, Maldonado AQ, Sjöholm K, Lonze BE, Montgomery RA, Runström A, et al. Outcomes at 3 years post-transplant in imlifidase-desensitized kidney transplant patients. Am J Transplant (2021) Jul 8. doi: 10.1111/ajt.16754.
23. Huang E, Maldonado AQ, Kjellman C, Jordan SC. Imlifidase for the treatment of anti-HLA antibody-mediated processes in kidney transplantation. Am J Transplant (2021) Sep 1. doi: 10.1111/ajt.16828.
24. Choi J, Aubert O, Vo A, Loupy A, Haas M, Puliyanda D, et al. Assessment of tocilizumab as a potential treatment for chronic antibody-mediated rejection and transplant glomerulopathy in HLA-sensitized renal allograft recipients. Am J Transplant (2017) 17:2381-89. doi: 10.1111/ajt.14228.
25. Banham GD, Flint SV, Torpey N, Lyons PA, Shanahan DN, Gibson A, et al. Belimumab in kidney transplantation: an experimental medicine, randomized, placebo-controlled phase 2 trial. Lancet (2018) 391:2619-26. doi: 10.1016/S0140-6736(18)30984-X.
26. Kwun J, Matignon M, Manook M, Guendouz S, Audard V, Kheav D, et al. Daratumumab in sensitized kidney transplantation: potentials and limitations of experimental and clinical use. J Am Soc Nephrol (2019) 30:1206-19. doi: 10.1681/ASN.2018121254.

27. Orandi BJ, Garonzik-Wang JL, Massie AB, Zachary AA, Montgomery JR, Van Arendonk KJ, et al. Quantifying the risk of incompatible kidney transplantation: a multicenter study. Am J Transplant (2014) 14:1573-80. doi: 10.1111/ajt.12786.
28. Orandi BJ, Luo X, Massie AB, Garonzik-Wang JM, Lonze BE, Ahmed R, et al. Survival benefit with kidney transplants from HLA-incompatible live donors. N Eng J Med (2016) 374:940-50. doi: 10.1056/NEJMoa1508380.
29. Manook M, Koeser L, Ahmed Z, Robb M, Johnson R, Shaw O, et al. Post-listing survival for highly sensitized patients on the UK kidney transplant waiting list: a matched cohort analysis. Lancet (2017) 389;727-34. doi: 10.1016/S0140-6736(16)31595-1.
30. Koo TY, Lee JH, Min SI, Lee Y, Kim MS, Ha J, et al. Presence of a survival benefit of HLA-incompatible living donor kidney transplantation compared to waiting or HLA-compatible deceased donor kidney transplantation with a long waiting time. Kidney Int (2021) 100:206-14. doi: 10.1016/j.kint.2021.01.027.
31. Clatworthy MR. Targeting B cells and antibody in transplantation. Am J Transplant (2011) 11:1359-67. doi: 10.1111/j.1600-6143.2011.03554.x.

Chapter 5: Outcomes after HLA Incompatible Transplantation

Nizam Mamode*, on behalf of Workstream 06 of the Transplantation Learning Journey of the European Society of Organ Transplantation
Department of Transplant Surgery, Guys Hospital, London, UK

*** Correspondence:** *Nizam Mamode, nizam.mamode@gstt.nhs.uk*

Keywords: kidney transplantation, HLA incompatible transplant, antibody incompatible transplant, outcomes, mortality, morbidity, quality of life

Word count (body text only): 1438
Number of figures: 0
Number of tables: 1

Abstract
HLA incompatible transplantation (HLAi) is defined as transplantation where the baseline complement-dependent cytotoxicity (CDC) or flow cytometric crossmatch is positive. Evidence regarding outcomes after HLAi is limited. Two studies from the US have shown a clear survival advantage for those having HLAi compared with matched patients who remain on the waiting list, but a large UK registry study found no survival advantage (or disadvantage). One US study found a lower rate of hospitalization after 3 years when HLAi recipients were compared with those who remained on the waiting list (RR 0.74; 95% CI 0.66–0.84; p<0.001). We found no data comparing quality of life in these groups. Although HLAi has become less popular in recent years, the data does not support the avoidance of this approach if the only alternative is the waiting list. Studies on quality of life after HLAi are urgently needed.

1 Introduction

There are two key issues which have proved problematic when considering outcomes after HLAi - the definition of HLAi and the comparison group. Most would accept that those with a positive flow cytometric or CDC crossmatch with their donor would fall into the category of HLAi. However, it is less certain whether those who have donor-specific antibodies (DSA) to their donor in solid phase assays, but a negative flow or CDC crossmatch should be considered as HLAi. Such patients are considered separately below, but it is important to realize that many studies reporting outcomes after HLAi include these patients within their groups. For this chapter, HLAi is defined as patients who are crossmatch positive. Published studies including DSA positive, crossmatch negative patients, but where the data from these patients could not be separated from crossmatch positive patents, have been excluded.
The second issue is a comparison group. Results from HLAi are often compared with those from compatible transplants, but the problem is that many HLAi patients will never

have the option of a compatible transplant, as the chance for the most highly sensitized to receive a deceased donor kidney, or matching in a kidney sharing scheme is essentially nil (1, 2). It is important, therefore, when considering outcomes, to also include patients who remain on dialysis and who are waiting for an organ offer as comparators.

The easiest outcome to consider is mortality, and although one would expect that this should be a straightforward outcome to determine, there are difficulties here. One problem is that of immortal time bias (3) (incidentally, a concept initially described in cardiac transplantation) - that is, if, for example, we consider those who have had an HLAi transplant, they have, by definition, not died prior to the transplant, while those on the waiting list who weren't transplanted might have died. This tends to overestimate the benefit of transplantation.

Many will be aware that patients undergoing HLAi may experience serious perioperative morbidity, and this should be easy to capture, although may be poorly reported. It is much harder to capture morbidity while remaining on dialysis, and to compare it. For example, would a post-transplant wound infection be of comparable importance to a patient as an infected aneurysm in a fistula, requiring admission and treatment? Thus, one might expect underestimation of the benefit of transplantation.

Patients undergoing HLAi are usually subjected to more powerful immunosuppressants, and therefore will be at higher risk of infections. Anecdotally, serious or unusual infections are a feature of transplanting such patients, so data on infections is important.

Finally, many patients who have been on dialysis for many years due to sensitization, invoke a poor quality of life as the reason for wanting an HLAi. They may be willing to accept increased risks due to the perception of significant benefit in their quality-of-life post-transplant, and therefore it is important to weigh this when considering outcomes.

This chapter will therefore consider the following:

- A comparison of mortality rates between HLAi and those who remain without a transplant
- A comparison of morbidity between HLAi and those who remain without a transplant
- A comparison of quality of life between HLAi and those who remain without a transplant

2 Mortality

There are only three studies comparing mortality in those who have undergone HLAi with those who remain on the waiting list, and these are detailed in **Table 1**. The study by Montgomery (4) compared outcomes from a single center with those in patients taken from the United Network for Organ Sharing (UNOS) database, matching them from the date of the transplant of the index patient in a 5 to 1 ratio. Matching criteria were well considered. There was a clear survival advantage for those who underwent HLAi compared with remaining on the waiting list, and this applied even when those who subsequently underwent a compatible transplant were considered (the 8-year patient survival rate for this group was 49%).

However, it might have been possible that the survival benefit shown for HLAi was due to the approach in this (expert) center, so in 2016, a study by Orandi (5) considered HLAi in 1025 patients from 22 centers in the US (these included 185 DSA positive, crossmatch negative patients). The results were strikingly similar.

However, the results from these studies have been partly contradicted by a UK registry study, which found no difference in survival when comparing 213 HLAi patients with 852 well-matched controls who remained on the waiting list (2). It is unclear why findings differ between the US and Europe, but one explanation may be a generally lower survival rate on dialysis in the US (6).

Nevertheless, no survival disadvantage for HLAi was found, suggesting that at the least HLAi may offer these patients a better quality of life, and at best, an improved quantity of life.

3 Morbidity

There are no studies that compare morbidity in those undergoing HLAi with those who remain on the waiting list. This is an important gap in our knowledge, particularly given the statements above regarding survival. There is one study by Orandi (7), which compared hospital readmissions in 379 HLAi transplants with matched controls who remained on the waiting list, using registry data from the US. Those who underwent HLAi, unsurprisingly, had a higher readmission rate in the first month (RR 5.86; 95% CI 4.96–6.92; p<0.001), but interestingly, had lower rates of hospitalization subsequently (at 3 years: RR 0.74; 95% CI 0.66–0.84; p<0.001). The data on reasons for admission do not permit detailed comparisons of morbidity, but the implication is that after an expected increased perioperative risk, those who undergo HLAi suffer fewer complications in the long term than those who remain on the waiting list.

A report by Kim (8) compared 56 HLAi (positive T cell flow cytometric crossmatches were excluded) with 274 compatible transplants, providing data on infectious complications, which may help in considering the risk. Urinary tract infections (41% vs 7.7%), cytomegalovirus viraemia (54% vs 14%) and pneumocystis jiroveci pneumonia (PJP) (5% vs 0%) were all significantly higher in the HLAi group (p<0.001). However, perhaps with the exception of PJP, it is difficult to be clear about the severity of these complications, and hence the cost to the patient. Another study which compared 27 HLAi patients with 69 ABOi patients, found no significant difference in viral, bacterial or fungal infections between the two groups, although of note, 6% of the ABOi group had PJP, compared with none of the HLAi group (9).

4 Quality of Life

We were unable to find any studies that compared quality of life in those undergoing HLAi, with those remaining on the waiting list and hoping for a compatible transplant.

This is clearly a major gap in our knowledge, since, given the statements regarding mortality above, will be the prime determinant for the most appropriate choice for patients.

5 Summary

We have found no evidence of increased mortality after HLAi compared with remaining on the waiting list, and, in the US, HLAi conferred a survival advantage.

There are few data concerning morbidity after HLAi in comparison with remaining on the waiting list, but there is some evidence that after the initial perioperative period, subsequent morbidity is lower.

There are no data on the quality of life after HLAi compared with remaining on the waiting list.

6 Recommendations

Areas for Further Research

We recommend that data be collected prospectively for sensitized patients, in order to compare the effect of an HLA incompatible transplant with remaining on the waiting list. This data should include:

- Mortality
- Morbidity
- Quality of Life (Chapters 2 and 5)

Abbreviations

CDC, complement-dependent cytotoxicity; DSA, donor-specific antibodies; HLA, human leukocyte antigen; KSS, kidney sharing scheme; MFI, mean fluorescence intensity; SAB, single antigen bead

Author Contributions

The members of WS06 of ESOT provided input and critical review of this chapter: Oriol Bestard, Frans Claas, Lucrezia Furian, Siân Griffin, Christophe Legendre, Maarten Naesens, Liset Pengel.

Conflict of Interest

The author declares that the research was conducted in the absence of any commercial or financial relationships that could be construed as a potential conflict of interest. The author has received honoraria from Hansa, Chiesi, Novartis and Takeda.

TABLE 1. Mortality in HLAi transplant recipients versus those not transplanted and remaining on the waiting list

	Country	Time (years)	Patient survival, %		p-value
			**HLAi transplant**	**No transplant, but on waiting list**	
Montgomery, 2011 (4)	USA	8	**80.6%** n=211	**30.5%** n=1050	p<0.001 [a]
Orandi, 2016 (5)	USA	8	**76.5%** n=1025	**43.9%** n=5125	p<0.001 [a]
Manook, 2017 (2)	UK	7	**78.3%** n=213	**76.9%** n=852	p=NS[b]

NS, not significantly different

a, Kaplan Meier; b, Kaplan Meier and log rank test

7 References

1. Jackson KR, Covarrubias K, Holscher CM, Luo X, Chen J, Massie AB, et al. The national landscape of deceased donor kidney transplantation for the highly sensitized: Transplant rates, waitlist mortality, and posttransplant survival under KAS. Am J Transplant (2019) 19:1129-38. doi: 10.1111/ajt.15149.
2. Manook M, Koeser L, Ahmed Z, Robb M, Johnson R, Shaw O, et al. Post-listing survival for highly sensitised patients on the UK kidney transplant waiting list: a matched cohort analysis. Lancet (2017) 389(10070):727-34. doi: 10.1016/S0140-6736(16)31595-1.
3. Lévesque LE, Hanley JA, Kezouh A, Suissa S. Problem of immortal time bias in cohort studies: example using statins for preventing progression of diabetes. BMJ (2010) 340:b5087. doi: 10.1136/bmj.b5087.
4. Montgomery RA, Lonze BE, King KE, Kraus ES, Kucirka LM, Locke JE, et al. Desensitization in HLA-incompatible kidney recipients and survival. N Engl J Med (2011) 365:318-26. doi: 10.1056/NEJMoa1012376.
5. Orandi BJ, Luo X, Massie AB, Garonzik-Wang JM, Lonze BE, Ahmed R, et al. Survival benefit with kidney transplants from HLA-incompatible live donors. N Engl J Med (2016) 374:940-50. doi: 10.1056/NEJMoa1508380.
6. Robinson BM, Akizawa T, Jager KJ, Kerr PG, Saran R, Pisoni RL. Factors affecting outcomes in patients reaching end-stage kidney disease worldwide: differences in access to renal replacement therapy, modality use, and haemodialysis practices. Lancet (2016) 388(10041):294-306. doi: 10.1016/S0140-6736(16)30448-2.
7. Orandi BJ, Luo X, King EA, Garonzik-Wang JM, Bae S, Montgomery RA, et al. Hospital readmissions following HLA-incompatible live donor kidney transplantation: A multi-center study. Am J Transplant (2018) 18:650-8. doi: 10.1111/ajt.14472.
8. Kim, DG, Lee J, Park Y, Kim MS, Jeong HJ, Kim SI, et al. Transplant outcomes in positive complement-dependent cytotoxicity- versus flow cytometry-crossmatch kidney transplant recipients after successful desensitization: a retrospective study. BMC Nephrol (2019) 20:456. doi: 10.1186/s12882-019-1625-2.
9. Couzi L, Manook M, Perera R, Shaw O, Ahmed Z, Kessaris N, et al. Difference in outcomes after antibody-mediated rejection between abo-incompatible and positive crossmatch transplantations. Transpl Int (2015) 28:1205-15. doi: 10.1111/tri.12621.

Chapter 6: The Place of Kidney Sharing Schemes for Sensitized Patients

Lucrezia Furian and Caterina Di Bella, on behalf of Workstream 06 of the Transplantation Learning Journey of the European Society of Organ Transplantation
Kidney and Pancreas Transplantation Unit, Department of Surgical Gastroenterological and Oncological Sciences, University Hospital of Padua, Padua, Italy

**** Correspondence:*** *Lucrezia Furian, lucrezia.furian@unipd.it*

Keywords*: *kidney paired donation, kidney sharing scheme, sensitized recipients, living donor kidney transplantation, HLA incompatibility, deceased donor-initiated chains

Word count (body text only): 4021
Number of figures: 0
Number of tables: 0

Abstract
Kidney Paired Donation (KPD) is a promising innovation in kidney transplantation, consisting of a considerable range of strategies developed for patients with a willing, but immunologically incompatible donor. One third of all potential live kidney donors are not suitable to donate a kidney to their intended recipients, due to human leukocyte antigen (HLA) or ABO blood group incompatibility. Incompatible living donor kidney transplantation (LDKT) is feasible using various desensitization protocols, but their outcomes are inferior compared with those from transplants with compatible living donors. Higher infection risk, major incidence of acute rejection and the need for stronger immunosuppression may compromise long-term graft outcome. There are also increased monetary costs involved and an increased length of hospitalization due to desensitization.

1 The Current Situation with Kidney Paired Donations

Rapaport first proposed the concept of KPD in 1986 (1), but it was first performed later, in 1991, in South Korea (2). This first KPD consisted of a simple, two-way swap between two incompatible pairs in a single transplant center. Over the years, KPD has shown encouraging results and is popular worldwide. Further expansion of KPD has led to the development of more complex systems and innovative solutions in order to maximize the number of exchanges.

The simplest form of KPD is a two-way exchange involving two incompatible pairs who swap their donors to achieve a compatible transplant for both recipients (**Figure 1)**.

The closed loop between three or more incompatible pairs whose recipients find a compatible kidney by exchanging their donors, represents another basic form of KPD. It consists of multiple surgeries (nephrectomies and transplants) that should be performed simultaneously allowing each pair to benefit from the swaps at the same time and preventing the risk of donor reneging.

These two forms of KPD work efficiently for pairs with blood type A/B incompatibility or for less immunized recipients. Unfortunately, for highly sensitized patients with a wide range of anti-HLA antibodies or for blood type O recipients, it is very hard to find a compatible match for each pair involved in a closed loop (please see Chapter 3, Figure 3 of this supplement [O. Bestard et al]).

The option of a non-directed altruistic (or unspecified) donor (NDAD) who is willing to donate his/her kidney with no intended recipient, is a real solution to the problem of reciprocal matching and avoids the need to 'close the loop'. The NDAD's kidney is matched with the recipient of an incompatible pair whose living donor donates to another incompatible recipient, initiating a domino-paired kidney exchange. The chain ends with the donor of the last pair donating to a recipient on the waiting list (WL) or waiting for another suitable match, starting another sequence of paired donations later (non-simultaneous extended altruistic donor chain, NEAD), thus becoming a bridge donor (**Figure 1)**. This model of KPD may include non-simultaneous surgeries and this is potentially associated with an incremental risk of donor reneging. Donors might decide not to donate once their intended recipients have been transplanted. Although broken chains are infrequent, and rarely due to lack of donor motivation, this risk might increase for bridge donors, who usually have to wait a while before donating. The occurrence of broken chains has been reported to be as low as 1.5%, with the most common causes for broken chains being bridge donor medical issues (0.46%), donors electing not to proceed (0.34%) and broken chains resulting from the kidney being declined by the recipient surgeon (0.23%)(3).

In 2016, Melcher, et al (4) proposed that a deceased donor organ should start a chain of living donor kidney transplants among incompatible pairs, but the first report of a successful deceased donor-initiated chain was published by Furian, et al in 2019 (5). In the DECeased donor kidney paired exchange (DEC-K) program, the chain-initiating kidney, selected from the deceased donor pool, is allocated to a recipient with an incompatible living donor and, at the end of the domino-chain, the living donor of the last pair donates to a WL patient (**Figure 1)**. Recipients of incompatible pairs are given priority in the allocation of a chain-initiating kidney from a deceased donor only in the absence of urgent, highly sensitized patients or candidates for combined transplants, according to the Italian policy for graft allocation (please see: http://www.trapianti.salute.gov.it/imgs/C_17_cntPubblicazioni_344_allegato.pdf). The program requires appropriate management of the ethical, allocation and logistic issues brought up by the very nature of the exchanges, but it is feasible. The major advantage of the DEC-K program is the ability to offer an opportunity for transplantation to recipients of incompatible donor pairs, but it also benefits WL candidates by allocating chain-ending kidneys from a living donor to them, prioritizing sensitized patients and those who have waited a long time for immunological reasons.

List exchange is another form of KPD, proposed by Delmonico et al, to prevent the issue of donor reneging (6). In this scheme, the donor of the incompatible pair donates before the recipient has received their compatible transplant from the deceased donor pool but, after donation, the paired recipient acquires priority over the WL candidates.

Other novel schemes of KPD take place in the setting of "chronological incompatibility" and constitute the advanced donation programs (ADPs) where a living donor donates

his/her kidney at his/her convenience to a recipient of an incompatible pair in need of transplant while his/her intended recipient will receive the reciprocal compatible kidney later on, when he/she actually needs a transplant (7).

A modification of ADP is the voucher system. A living donor donates their kidney as a NDAD, starting a domino chain or donates directly to a candidate on the WL, while the intended recipient gets a voucher for the future. When the recipient is in need of a transplant, he/she will have priority for graft allocation on the deceased donor WL. This model seems to work efficiently in the case of pairs whose donor might become too old to donate by the time the recipient really needs transplantation.

A strong correlation between the number of pairs enrolled in a kidney exchange program and the success of matching more possible pairs has been widely demonstrated. That said, participation of ABO and HLA compatible pairs appears to be a brilliant strategic move for further expanding the pool and, therefore, the number of successful matches. This model provides undeniable benefits for recipients of incompatible pairs, whereas advantages for compatible pairs seem more questionable. Receiving a kidney from a younger donor, with negative serology for cytomegalovirus or Epstein-Barr virus or getting a better HLA match or weight match might represent an appealing gain for recipients of compatible pairs, encouraging their participation in exchange programs. A recent report from the National Kidney Registry linked to data from the Scientific Registry of Transplant Recipients identified 154 compatible pairs involved in paired exchange programs, seeking to improve their HLA matching through an exchange. These patients obtained a transplant from younger donors, with higher estimated glomerular filtration rate and body mass index and a better score on the living kidney donor profile index as compared with their original donor (8).

Although KPD was conceived as an alternative for incompatible pairs to avoid their recipients undergoing expensive and risky desensitization protocols, another strategy to improve KPD results is combining exchange programs with desensitization. ABO incompatible transplantation in the absence of donor specific antibodies (DSA) provides excellent transplantation results. Combining exchange programs with desensitization extends this to accepting ABO incompatible living donors against whom recipients have lower anti-blood group antibody titers in the setting of KPD. This strategy has been successfully applied in the Australian program and by Montgomery, at the John Hopkins Institute (9). In the Australian experience, ABO-incompatible donors were accepted with anti-A and/or anti-B titres usually 1:64 or less. With this selective incorporation of ABO incompatibility, 10 recipients found a suitable match and were transplanted. These recipients were distributed across eight chains (three two-way and five three-way chains) resulting in 21 recipients being successfully transplanted. It should be noted that, of the patients transplanted through the program with an ABO-incompatible kidney, 54% had a cPRA of >75% and 36% had a cPRA of >90% (10).

Trans-organ paired exchange represents the most innovative concept of KPD. It can be helpful in circumstances when a donor is not suitable for donating a kidney but is still fit to donate other organs for exchange. For example, a living kidney donor who is not eligible for renal donation but can donate his/her liver to a liver recipient of a pair whose donor is ruled out from liver donation but is suitable for kidney donation. Torres, et al published the first case of trans-organ exchange, attracting many criticisms related to

the surgical risk of donation that is very different for different organs (11).

2 Desensitization Versus KPD: Outcome in ABO and HLA Incompatible Patients

KPD and desensitization have traditionally been considered competing strategies to solve the immunological incompatibility that can mitigate living donation. Published literature assists physicians in making decisions regarding the choice of KPD over desensitization or vice versa, when encountering an incompatible pair for LDKT. Risks and benefits relating to the possible strategies - KPD or desensitization - should be outlined and discussed with patients.

Outcomes of ABO incompatible transplantation after desensitization have proved to be excellent over the years (12 – 14), but have highlighted the fact that blood type O recipients have low match rates and long wait list times (15 – 17). There is also a lack of blood type O donors - with blood type O being predominant among recipients but underrepresented in the domino-donor population (18, 19). Blood type O donors are always blood group compatible with their intended recipients, so the only reason why they would join a KPD program is the presence of unacceptable DSA in the recipient, causing a positive crossmatch.

With graft outcomes following ABOi LDKT being comparable with ABO compatible LDKT (20, 21) probably the most convenient option for a blood type O recipient who has an ABOi living donor is desensitization, especially if they present with an acceptable baseline antibody titer and are more likely to be desensitized. For these patients, KPD should be offered as a first option, particularly when desensitization is unsuccessful or for those with very high antibody titer, which may require aggressive immunosuppressive therapy and intensive desensitization protocols, thus increasing the risk-benefit ratio. In countries with a well-developed KPD program, KPD should also be offered to low-titre ABOi recipients, given the advantages of avoiding desensitization, unless the patient declines this option.

Things are very different for HLA incompatible (HLAi) pairs. Desensitization protocols have been applied in cases of sensitized recipients who have a willing, but incompatible living donor due to the presence of DSA (9, 22). Certainly, HLAi LDKT after desensitization provided a significant survival benefit for these patients, compared with remaining on dialysis. Montgomery, et al (9) demonstrated in a cohort of 211 HLA-sensitized patients who were desensitized and subsequently transplanted thanks to their incompatible donor, that patient survival was 90.6% at 1 year, 85.7% at 3 years, 80.6% at 5 years, and 80.6% at 8 years, as compared with rates of 91.1%, 67.2%, 51.5%, and 30.5%, respectively, for patients in the dialysis group. However, poor outcomes have been reported after HLAi LDKT compared with HLA compatible LDKT. A 1.64-fold and 5.01-fold increased risk of graft loss at 1-year for recipients with a positive flow cytometric crossmatch and positive cytotoxic crossmatch, respectively, is reported in the literature (23). This may be a consequence of increased post-operative complications, including delayed graft function and acute rejection (24 – 26) given the potential risk of post-desensitization rebound of DSA. Moreover, need for intensive immunomodulation (infusion of intravenous immunoglobulin, anti-B-cell agents, other agents such as eculizumab, bortezomib, a C1 esterase inhibitor, sessions of plasmapheresis or rescue splenectomy), in addition to post-transplant immunosuppressive medications exposes these patients to a serious risk of infection (27).

In 2005, Segev, et al proved, by a simulation based on UNOS data, the superiority of KPD over desensitization, guaranteeing better graft outcomes and higher transplantation rates for HLAi pairs (28). Interestingly, 47% of the HLAi pairs could have been matched through an optimized national KPD program. The authors clearly stated that KPD should be the preferred treatment for patients who have HLA incompatibilities with their willing donors, as it is less expensive compared with desensitization and requires less immunosuppression (28).

Bingaman, et al identified three disadvantaged patient categories who would particularly benefit from KPD programs: highly sensitized recipients, multiparous females and retransplant patients (29). The KPD program improved their access to transplantation and offered them excellent graft outcomes and low rejection rates.

However, despite the implementation of KPD strategies, in the United States, patients with a PRA of 99.9% remain the most disadvantaged transplant candidates with prolonged waiting times and high waiting list mortality (30). In fact, patients with a cPRA >80% were less likely to receive a LDKT (6.5%) compared with candidates with a cPRA <80% (26.7%), and in the 99% cPRA group, only 3.4% of all transplants were from a paired KPD donor, and only 1.3% in 100% cPRA candidates. This is why some transplantation centers still promote desensitization as a valid and needed approach to increase the probability of transplantation in highly sensitized patients (31). Others have proposed KPD only in cases of failed desensitization procedures, as a kind of “rescue” therapy (32).

To resolve the dilemma of whether to use a desensitization protocol versus KPD for incompatible pairs, an Italian group proposed a decisional algorithm including and integrating both strategies in a unique flowchart (33). They analyzed the outcomes of 54 patients transplanted at Pisa Transplantation Centre, between 2005 and 2017, applying KPD or desensitization therapy. Results achieved with KPD versus those achieved with desensitization for the main groups of incompatibility (ABO and HLA) were compared. No significant differences among the groups were recorded in terms of patient and graft survival. However, DSA+ desensitized patients proved to be more prone to produce de-novo DSA after LDKT and when the DSA titer was high (>3000 mean fluorescence intensity, MFI), recipients showed a higher risk of acute rejection (50% vs 14%). Furthermore, desensitization strategies were more expensive, with a cost equal to 3 months of dialysis. The authors concluded that for HLAi couples, a KPD strategy should always be preferred. For ABOi pairs, desensitization protocols or KPD offer comparable results, differing only by cost, but KPD requires a 3-month prolongation of dialysis while waiting for a compatible match.

3 Strategies to Expand the KPD Pool

KPD match rates are dependent on the number of incompatible pairs enrolled in a program (28, 34); hence, expanding the pool size is critical to the implementation of KPD. ABOi pairs represent an important source for the overall KPD pool; if the number of ABOi registered pairs is lower than those with HLA incompatibility, match probability decreases (35). Furthermore, a KPD pool with several ABOi pairs would potentially offer the opportunity of a higher match probability by accepting ABO incompatibilities in the exchanges. The Australian KPD program has applied this strategy since 2013 (36),

showing an enhancement in transplant rate for all KPD enrolled patients. Ferrari, et al confirmed the same result later with 17 out of 92 transplanted patients from the Australian registry receiving a kidney from an ABOi matched donor. These recipients were distributed across 15 domino chains, which could only have been realized if ABOi matching was accepted (37).

The integration of desensitization protocols in KPD programs can also consist of allowing low-risk crossmatch-incompatible kidney transplantation in highly-sensitized patients, in the setting of paired donation. This is another promising approach, strongly endorsed in the literature (38, 39), and developed to improve KPD efficiency by increasing the transplant rate of highly sensitized patients and reducing their wait time for a LDKT. In 2013, Blumberg, et al proposed a protocol including acceptable crossmatch-incompatible donors and the administration of intravenous immunoglobulin to transplant 12 HLA-sensitized patients (median calculated PRA: 98%) with allografts from the KPD program. In the Californian experience, KPD was successfully performed across crossmatch-incompatible transplants, representing another viable chance of an organ for very sensitized recipients (40). A more complex model of integration is represented by Computerized Integration of Alternative Transplantation Programs (CIAT), recently developed in The Netherlands to integrate paired exchange, altruistic donation and ABO/HLA-desensitization (41). To compare CIAT with reality, a simulation was performed on data from the Dutch Living Donor Kidney Program and included difficult-to-match and highly immunized patients (virtual PRA >85%). HLAi matching with DSA-MFI <8000 was allowed, as well as ABOi matched for long-waiting blood group O or B patients. Compared with reality, the simulation results showed that CIAT would have led to better transplant opportunities for difficult-to-match patients and highly sensitized patients, and more ABO compatible matches, without compromising the total number of matches.

Another concept to increase KPD pair numbers and, accordingly, the number of successful matches within the same pool, is the Unbalanced Paired Donation, consisting of the inclusion of ABO/HLA compatible pairs in KPD programs (42, 43). This strategy was initially proposed as an attempt to facilitate transplantation for patients in the most disadvantageous categories in a KPD program: blood group O recipients and those who are highly sensitized (44 - 46); and a mathematical analysis conducted in 2007 found that including compatible pairs (CC) in a KPD program would correct the blood group imbalance that usually characterizes a pool of ABOi/HLAi pairs (47). In fact, all recipients of incompatible pairs benefited: their chance of receiving a kidney from a compatible donor doubled from 28% to 65% for a single-center program and from 37% to 75% for a national one. Looking at real data from the analysis of the first 9 years of the National Kidney Register, the participation of CC facilitated 146 transplants, including 43 recipients with PRA>80% (48).

Overall, if there is a striking gain for incompatible pairs, CCs participating in a KPD program are disadvantaged, waiting for a match and postponing the transplant surgery that would have been otherwise performed. The altruism behind their participation should be balanced by giving them a potential benefit. From published data, the main reasons why CCs join a KPD program are size/age mismatch, cytomegalovirus or Epstein-Barr

virus serology mismatch between donor and intended recipient, the opportunity to receive a better HLA match, avoiding complex donor kidney anatomy or pure altruism (48 – 50).
Successful single-center experiences of KPD including CC, have been able to provide recipients of CC with kidneys from younger donors (29) or kidneys with a better Living Donor Profile Index score, as a predictor of long-term graft survival (49). This “gain” of receiving a “better matched kidney” for a CC entering KPD programs is considered, by some authors, to be risky, as it might require time to find a better match, thus delaying transplantation, and the estimated quality of the graft might misrepresent the real outcome of the transplant. To answer these points, Gill, et al introduced the concept of the “reciprocity-based strategy” in which the recipient of a CC acquires priority for a repeat deceased donor transplant in case the LDKT fails (51). The authors highlight how this strategy would be embraced more willingly by CC, since it guarantees them a significant and concrete benefit. Despite the ethical concerns about the inclusion of CC in a KPD program, the Unbalanced Paired Donation, by expanding the pool, results in increasing the overall number of LDKTs and facilitating access to transplantation for the most sensitized candidates enrolled in the program.
The last way to widen the number of patients enrolled in a KPD program, is the creation of transnational registers. Indeed, the inclusion of international pairs offers a higher probability of finding a compatible donor, especially for difficult-to-match recipients who have less opportunities to be transplanted within a national or local program. Some authors hypothesized that differences in HLA antigen prevalence across different ethnicities may play an important role in KPD matching (52). Hence, sensitized recipients presenting with DSA against several donors available in their national pool, may find a compatible match more easily among donors of another race or from other countries.
National KPD programs may differ on ethical viewpoints, legal and financial frameworks, clinical practices and population size depending on the country. Whenever a collaboration is established for a transnational KPD, the different regional models need to merge together and reach a compromise that suits all the cultures involved in the cooperation for it to be successful. The goal is for the collaboration to appropriately benefit all populations, recipients and donors involved (53).
Transnational kidney exchanges have been successfully realized across Europe and USA. The international cooperation between Italy, Spain and Portugal has led to 2 two-way exchanges (54), the Czech-Austrian KPD program, involving patients from Austria, Germany, Slovenia and Ukraine facilitated 81 transplants (55) while 38 LDKTs were performed through six chains and two cycles between 30 US patients and eight non-US patients, of whom 11 presented with a PRA>80% (56). At the Mayo Clinic, in 10 years of KPD, a small group of pairs were from outside of the US (49). Their participation enabled highly sensitized patients to receive a compatible transplant because 75% of chains/swaps included an international pair, and also a recipient with a PRA of at least 90%.
These results demonstrate the feasibility of merging small national KPD programs to increase the pool size and encourage the development of international registries to optimize the KPD resource.

4 Logistical Issues of KPD Programs

A KPD program, before starting, requires an extensive assessment of all logistic, legal and ethical issues, including concerns regarding increased times of cold ischemia (CIT) and the risk of donor renege, which might affect the outcomes of the program.

When a kidney exchange involves two or more different transplant centers, shipping the organs rather than asking donors to travel to the recipient center seems to be the favored choice. Usually, both donors and recipients feel much more comfortable undergoing surgery at their trusted transplant center. However, a shipped kidney implies a longer CIT compared with standard LDKT, and a possible increase in the probability of graft failure has been historically a cause for concern. In a 2020 study, conducted on 10-years of transplant activity in the National Kidney Register, extended CIT proved not to be predictive of donor graft failure or graft outcome (57).

The Italian DEC-K experience confirms that shipping kidneys is safe and does not affect graft or patient survival even when the graft comes from a deceased donor, as in the case of a chain initiating kidney, which requires time for organ procurement as well as travel time (mean CIT: 7 hours) (18). It has to be mentioned that distances in the Italian territory are generally conducive for short CIT. However, even when shipped distances increase and CITs increase accordingly, as in the US (58, 59) or Australia (60) or in transnational KPD programs (54, 61), no association between extended CIT and donor graft failure or graft loss were found.

Certainly, a KPD program limited to a single center would succeed in keeping CIT as short as possible but it would only realize a very small number of transplants, given the availability of a small pool size. Pushing the boundaries for acceptable prolonged CIT has helped to expand and optimize KPD by making the utilization of kidneys originating from distant transplant centers possible.

The other major topic when discussing KPD, is the risk of a donor reneging, and the probability of this may vary according to the type of paired donation. Performing surgical procedures simultaneously within a closed loop is logistically difficult and requires a great deal of careful coordination, but it does minimize the risk of donors reneging. The complexity grows proportionately with the number of pairs involved in the loop since all donors should undergo nephrectomy concurrently, ensuring that no donors withdraw after their paired recipient received their kidney transplant. List exchange and Advanced Donation Programs likewise prevent the issue of donor renege, but also require a bigger contribution from donors who have to donate before their intended recipient gets a compatible transplant.

The risk of donor renege is potentially higher in domino-paired donation. Unavoidably, surgeries cannot be simultaneous within a chain and a donor withdrawal would cause a premature break in the chain, leaving a recipient orphaned, despite the fact that his/her paired donor has already donated. This risk increases when donors wait a long time before donating (62), as in the case of bridge donors or when it takes too much time to schedule a continuous chain. Loss of donor motivation or changes in the donor's state of health might occur, affecting the success of the entire KPD program and reducing interest in pursuing this option for kidney transplantation. In addition to a deep psychological assessment, donors and recipients should go through an educational process to fully comprehend the principles and functioning of domino kidney paired

exchange before giving their consent to participate in the program. As highlighted by Furian, et al in the first report of the DEC-K experience, this is essential to prevent the risk of donor renege (5). Other strategies to avoid premature ending of kidney chains are scheduling donor nephrectomy soon after the paired recipient has received their transplant and, as suggested by the Dutch experience, to construct short length chains (63).

5 Recommendations

Organ Allocation

- Access to the donor pool should be increased through greater use of:
  - Increased access to and harmonization of Kidney Exchange Programs, with greater and standardized sharing of outcomes (Chapters 2 and 6)
  - Inclusion of unspecified kidney donations (if these are performed) in kidney sharing schemes (Chapters 2 and 6)
  - Inclusion of compatible pairs and deceased donor organs in kidney sharing schemes
- Kidney Paired Donation is the preferred initial option over desensitization given the better transplant outcomes and cost-effectiveness, in both ABO and HLA incompatible pairs, unless there is a need for desensitization, there is clinical urgency or a low chance of a transplant

Abbreviations

ADP, advanced donation program; cc, compatible pair; CIAT, Computerized Integration of Alternative Transplantation Programs DSA, donor-specific antibodies; %PRA, percentage of positive panel donors in an antibody screening assay; HLA, human leukocyte antigen; KPD, kidney paired donation; KSS, kidney sharing scheme; LDKT, living donor kidney transplant; MFI, mean fluorescence intensity; NDAD, non-directed altruistic donor; NEAD, non-simultaneous extended altruistic donor

Author Contributions

The members of WS06 of ESOT provided input and critical review of this chapter: Oriol Bestard, Frans Claas, Siân Griffin, Christophe Legendre, Nizam Mamode, Maarten Naesens, Liset Pengel.

Conflict of Interest

The authors declare that the research was conducted in the absence of any commercial or financial relationships that could be construed as a potential conflict of interest. LF has received honoraria from Hansa, Chiesi, Novartis and Astellas.

FIGURE 1. Examples of kidney paired donation exchanges **(A)** Two-way exchange **(B)** Three-way exchange **(C)** Domino-chain ending with a donation to a wait-list patient or a bridge donor and starting from a non-directed altruistic donor (NDAD), a non-simultaneous extended altruistic donor (NEAD), or a deceased donor (Dec-K program).

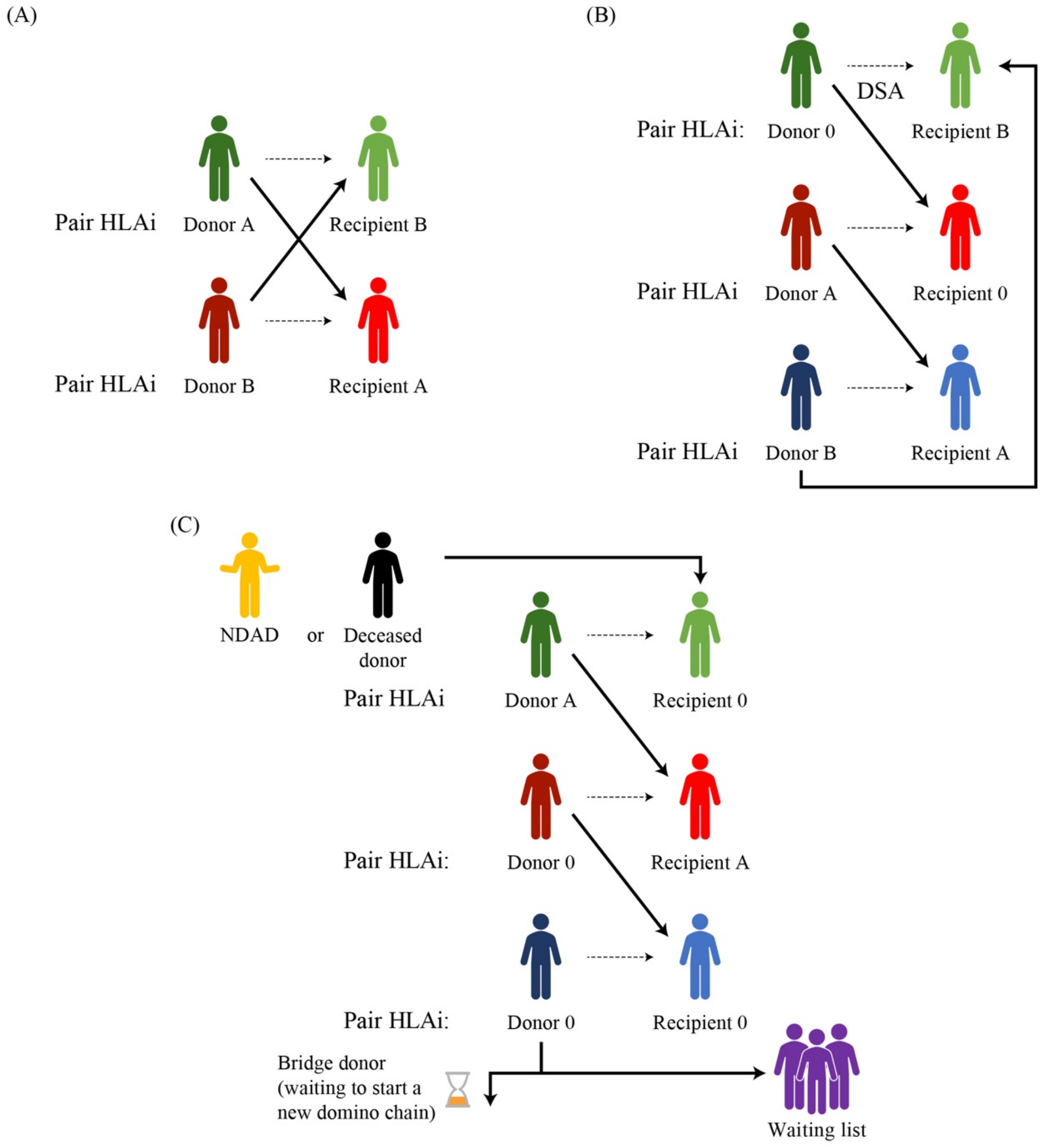

6 References

1. Rapaport FT. The case for a living emotionally related international kidney donor exchange registry. Transplant Proc (1986) 18 Suppl. 2:5-9.
2. Kwak JY, Kwon OJ, Lee KS, Kang CM, Park HY, Kim JH. Exchange-donor program in renal transplantation: a single center experience. Transplant Proc (1999) 31:344-5. doi: 10.1016/s0041-1345(98)01655-8.
3. Cowan N, Gritsch HA, Nassiri N, Sinacore J, Veale J. Broken chains and reneging: A review of 1748 kidney paired donation transplants. Am J Transplant (2017) 17:2451-7. doi: 10.1111/ajt.14343.
4. Melcher ML, Roberts JP, Leichtman AB, Roth AE, Rees MA. Utilization of deceased donor kidneys to initiate living donor chains. Am J Transplant (2016) 16:1367-70. doi: 10.1111/ajt.13740.
5. Furian L, Cornelio C, Silvestre C, Neri F, Rossi F, Rigotti P, et al. Deceased donor–initiated chains: First report of a successful deliberate case and its ethical implications. Transplantation (2019) 103:2196-200. doi: 10.1097/TP.0000000000002645.
6. Delmonico FL, Morrissey PE, Lipkowitz GS, Stoff JS, Himmelfarb J, Harmon W, et al. Donor kidney exchanges. Am J Transplant (2004); 4:1628-34. doi: 10.1111/j.1600-6143.2004.00572.x.
7. Veale JL, Nassiri N, Capron AM, Danovitch GM, Gritsch HA, Cooper M, et al. Voucher-based kidney donation and redemption for future transplant. JAMA Surg (2021) 156:812-7. doi: 10.1001/jamasurg.2021.2375.
8. Chipman V, Cooper M, Thomas AG, Ronin M, Lee B, Flechner S, et al. Motivations and outcomes of compatible living donor-recipient pairs in paired exchange. Am J Transplant (2021). doi: 10.1111/ajt.16821.
9. Montgomery RA, Lonze BE, King KE, Kraus ES, Kucirka LM, Locke JE, et al. Desensitization in HLA-incompatible kidney recipients and survival. N Engl J Med (2011) 365:318-26. doi: 10.1056/NEJMoa1012376.
10. Ferrari P, Hughes PD, Cohney SJ, Woodroffe C, Fidler S, D'Orsogna L. ABO-incompatible matching significantly enhances transplant rates in kidney paired donation. Transplantation (2013) 96:821-6. doi: 10.1097/TP.0b013e3182a01311.
11. Torres AM, Wong F, Pearson S, Weinberg S, Roberts JP, Ascher NL, et al. Bi-organ paired exchange-Sentinel case of a liver-kidney swap. Am J Transplant (2019) 19:2646-9. doi: 10.1111/ajt.15386.
12. Scurt FG, Ewert L, Mertens PR, Haller H, Schmidt BMW, Chatzikyrkou C. Clinical outcomes after ABO-incompatible renal transplantation: a systematic review and meta-analysis. Lancet (2019) 393(10185):2059-72. doi: 10.1016/S0140-6736(18)32091-9.
13. Loupy A, Bouquegneau A, Stegall MD, Montgomery RA. Clinical outcomes after ABO-incompatible renal transplantation. Lancet (2019) 394(10213):1988-9. doi: 10.1016/S0140-6736(19)32490-0.
14. Roodnat JI, van de Wetering J, Claas FH, Ijzermans J, Weimar W. Persistently low transplantation rate of ABO blood type O and highly sensitized patients despite

alternative transplantation programs. Transpl Int (2012) 25:987-93. doi: 10.1111/j.1432-2277.2012.01526.x.
15. Cole EH, Nickerson P, Campbell P, Yetzer K, Lahaie N, Zaltzman J, et al. The Canadian kidney paired donation program: a national program to increase living donor transplantation. Transplantation (2015) 99:985-90. doi: 10.1097/TP.0000000000000455.
16. Holscher CM, Jackson K, Chow EKH, Thomas AG, Haugen CE, DiBrito SR, et al. Kidney exchange match rates in a large multicenter clearinghouse. Am J Transplant (2018) 18:1510-7. doi: 10.1111/ajt.14689.
17. Segev DL, Gentry SE, Melancon JK, Montgomery RA. Characterization of waiting times in a simulation of kidney paired donation. Am J Transplant (2005) 5:2448-55. doi: 10.1111/j.1600-6143.2005.01048.x.
18. Furian L, Nicolò A, Di Bella C, Cardillo M, Cozzi E, Rigotti P. Kidney exchange strategies: new aspects and applications with a focus on deceased donor-initiated chains. Transpl Int (2020) 33:1177-84. doi: 10.1111/tri.13712.
19. Roodnat JI, Zuidema W, Van De Wetering J, De Klerk M, Erdman RAM, Massey EK, et al. Altruistic donor triggered domino-paired kidney donation for unsuccessful couples from the kidney-exchange program. Am J Transplant (2010) 10:821-7. doi: 10.1111/j.1600-6143.2010.03034.x.
20. Tydén G, Kumlien G, Genberg H, Sandberg J, Lundgren T, Fehrman I. ABO incompatible kidney transplantations without splenectomy, using antigen-specific immunoadsorption and rituximab. Am J Transplant (2005) 5:145-8. doi: 10.1111/j.1600-6143.2004.00653.x.
21. Tanabe K. Japanese experience of ABO-incompatible living kidney transplantation. Transplantation (2007) 84(12 Suppl):S4-7. doi: 10.1097/01.tp.0000296008.08452.4c.
22. Orandi BJ, Luo X, Massie AB, Garonzik-Wang JM, Lonze BE, Ahmed R, et al. Survival benefit with kidney transplants from HLA-incompatible live donors. N Engl J Med (2016) 374:940-50. doi: 10.1056/NEJMoa1508380.
23. Orandi BJ, Garonzik-Wang JM, Massie AB, Zachary AA, Montgomery JR, Van Arendonk KJ, et al. Quantifying the risk of incompatible kidney transplantation: a multicenter study. Am J Transplant (2014) 14:1573-80. doi: 10.1111/ajt.12786.
24. Haller J, Wehmeier C, Hönger G, Hirt-Minkowski P, Gürke L, Wolff T, et al. Differential impact of delayed graft function in deceased donor renal transplant recipients with and without donor-specific HLA-antibodies. Transplantation (2019) 103:e273-e280. doi: 10.1097/TP.0000000000002802.
25. Orandi BJ, Chow EH, Hsu A, Gupta N, Van Arendonk KJ, Garonzik-Wang JM, et al. Quantifying renal allograft loss following early antibody-mediated rejection. Am J Transplant (2015) 15:489-98. doi: 10.1111/ajt.12982.
26. Orandi BJ, Luo X, King EA, Garonzik-Wang JM, Bae S, Montgomery RA, et al. Hospital readmissions following HLA-incompatible live donor kidney transplantation: a multi-center study. Am J Transplant (2018) 18:650-8. doi: 10.1111/ajt.14472.
27. Avery RK, Motter JD, Jackson KR, Montgomery RA, Massie AB, Kraus ES, et al. Quantifying infection risks in incompatible living donor kidney transplant recipients. Am J Transplant (2021) 21:1564-75. doi: 10.1111/ajt.16316.

28. Segev DL, Gentry SE, Warren DS, Reeb B, Montgomery RA. Kidney paired donation and optimizing the use of live donor organs. JAMA (2005) 293:1883-90. doi: 10.1001/jama.293.15.1883.
29. Bingaman AW, Wright FH Jr, Kapturczak M, Shen L, Vick S, Murphey CL. Single-center kidney paired donation: the Methodist San Antonio experience. Am J Transplant (2012) 12:2125-32.doi: 10.1111/j.1600-6143.2012.04070.x.
30. Jackson KR, Covarrubias K, Holscher CM, Luo X, Chen J, Massie AB, et al. The national landscape of deceased donor kidney transplantation for the highly sensitized: Transplant rates, waitlist mortality, and posttransplant survival under KAS. Am J Transplant (2019) 19:1129-38. doi: 10.1111/ajt.15149.
31. Schinstock CA, Smith BH, Montgomery RA, Jordan SC, Bentall AJ, Mai M, et al. Managing highly sensitized renal transplant candidates in the era of kidney paired donation and the new kidney allocation system: is there still a role for desensitization? Clin Transplant (2019) 33:e13751. doi: 10.1111/ctr.13751.
32. Sharif A, Zachary AA, Hiller J, Segev D, Alachkar N, Kraus ES, et al. Rescue kidney paired donation as emergency salvage for failed desensitization. Transplantation (2012) 93:e27-9. doi: 10.1097/TP.0b013e318249b10e.
33. Vistoli F, Perrone V, Gozzini L, Kauffmann E, Napoli N, Iacopi S, et al. Desensitization protocols vs kidney paired donation to overcome AB0 incompatibility and/or donor specific antibodies in living donor kidney transplantation [abstract]. Am J Transplant (2019) 19 (suppl 3). https://atcmeetingabstracts.com/abstract/desensitization-protocols-vs-kidney-paired-donation-to-overcome-ab0-incompatibility-and-or-donor-specific-antibodies-in-living-donor-kidney-transplantation/. Accessed January 13, 2021).
34. Saidman SL, Roth AE, Sönmez T, Unver MU, Delmonico FL. Increasing the opportunity of live kidney donation by matching for two- and three-way exchanges. Transplantation (2006) 81:773-82. doi: 10.1097/01.tp.0000195775.77081.25.
35. Johnson RJ, Allen JE, Fuggle SV, Bradley JA, Rudge C, Kidney Advisory Group, UK Transplant NHSBT. Early experience of paired living kidney donation in the United Kingdom. Transplantation (2008) 86:1672-7. doi: 10.1097/TP.0b013e3181901a3d.
36. Ferrari P, Hughes PD, Cohney SJ, Woodroffe C, Fidler S, D'Orsogna L. ABO-incompatible matching significantly enhances transplant rates in kidney paired donation. Transplantation (2013) 96:821-6. doi: 10.1097/TP.0b013e3182a01311.
37. Ferrari P, Weimar W, Johnson RJ, Lim WH, Tinckam KJ. Kidney paired donation: principles, protocols and programs. Nephrol Dial Transplant (2015) 30:1276-85. doi: 10.1093/ndt/gfu309.
38. Karami F. Optimal integration of desensitization protocols into kidney paired donation programs. Operations Research for Health Care (2019) 32:100198. https://doi.org/10.1016/j.orhc.2019.100198 [Accessed 24 September 2021].
39. Yabu JM, Pando MJ, Busque S, Melcher ML. Desensitization combined with paired exchange leads to successful transplantation in highly sensitized kidney transplant recipients: strategy and report of five cases. Transplant Proc (2013) 45:82-7. doi: 10.1016/j.transproceed.2012.08.007.

40. Blumberg JM Gritsch HA, Reed EF, Cecka JM, Lipshutz GS, Danovitch GM, et al. Kidney paired donation in the presence of donor specific antibodies. Kidney Int (2013) 84:1009-16. doi: 10.1038/ki.2013.206.
41. De Klerk M, Kal-van Gestel JA, van de Wetering J, Kho ML, Middel-de Sterke S, Betjes MGH, et al. Creating options for difficult to match kidney transplant candidates. Transplantation (2021) 105:240-8. doi: 10.1097/TP.0000000000003203.
42. Roth AE, Sönmez T, Unver MU. Kidney paired donation with compatible pairs. Am J Transplant (2008) 8:463. doi: 10.1111/j.1600-6143.2007.02052.x.
43. Ratner LE, Rana A, Ratner ER, Ernst V, Kelly J, Kornfeld D, et al. The altruistic unbalanced paired kidney exchange: proof of concept and survey of potential donor and recipient attitudes. Transplantation (2010) 89:15-22. doi: 10.1097/TP.0b013e3181c626e1.
44. Kute VB, Gumber MR, Shah PR, Patel HV, Vanikar AV, Modi PR, et al. Successful three-way kidney paired donation transplantation: The first Indian report. Indian J Nephrol (2014) 24:45-7. doi: 10.4103/0971-4065.125094.
45. Ross LF, Woodle ES. Ethical issues in increasing living kidney donations by expanding kidney paired exchange programs. Transplantation (2000) 69:1539-43. doi: 10.1097/00007890-200004270-00001.
46. Kranenburg LW, Zuidema W, Weimar W, Passchier J, Hilhorst M, de Klerk M, et al. One donor, two transplants: willingness to participate in altruistically unbalanced exchange donation. Transpl Int (2006) 19:995-9. doi: 10.1111/j.1432-2277.2006.00378.x.
47. Gentry SE, Segev DL, Simmerling M, Montgomery RA. Expanding kidney paired donation through participation by compatible pairs. Am J Transplant (2007) 7:2361-70.doi: 10.1111/j.1600-6143.2007.01935.x.
48. Flechner SM, Thomas AG, Ronin M, Veale JL, Leeser DB, Kapur S, et al. The first 9 years of kidney paired donation through the National Kidney Registry: Characteristics of donors and recipients compared with National Live Donor Transplant Registries. Am J Transplant (2018) 18(11):2730-8. doi: 10.1111/ajt.14744.
49. Basu A, Prieto M, Kosberg C, Mai ML, Khamash HA, Jadlowiec CC, et al. Ten years of kidney paired donation at Mayo Clinic: The benefits of incorporating ABO/HLA compatible pairs. Transplantation (2020) 104:1229-38. doi: 10.1097/TP.0000000000002947.
50. Weng FL, Grogan T, Patel AM, Mulgaonkar S, Morgievich MM. Characteristics of compatible pair participants in kidney paired donation at a single center. Clin Transplant (2017) 31:10.1111/ctr.12978. doi: 10.1111/ctr.12978.
51. Gill JS, Tinckam K, Fortin MC, Rose C, Shick-Makaroff K, Young K, et al. Reciprocity to increase participation of compatible living donor and recipient pairs in kidney paired donation. Am J Transplant (2017) 17:1723-8. doi: 10.1111/ajt.14275.
52. Alabadi A. Simulation of international kidney exchange between Saudi Arabia and the United States using actual incompatible pair data. Congress abstract – 26th International Congress of the Transplantation Society, TTS 2016. Hong Kong. Transplantation 2016.
53. Biró P, Haase-Kromwijk B, Andersson T, Ásgeirsson EI, Baltesová T, Boletis I, et al; ENCKEP COST Action. Building kidney exchange programmes in Europe-An

overview of exchange practice and activities. Transplantation (2019) 103:1514-22. doi: 10.1097/TP.0000000000002432.
54. Valentín MO, Garcia M, Costa AN, Bolotinha C, Guirado L, Vistoli F, et al. International cooperation for kidney exchange success. Transplantation (2019) 103:e180-e181. doi: 10.1097/TP.0000000000002664.
55. Viklicky O, Krivanec S, Vavrinova H, Berlakovich G, Marada T, Slatinska J, et al. Crossing borders to facilitate live donor kidney transplantation: the Czech-Austrian kidney paired donation program - a retrospective study. Transpl Int (2020) 33:1199-210. doi: 10.1111/tri.13668.
56. Rees M. International kidney exchanges providing living donor kidney transplants for 38 patients involving 18 transplant centers from six countries. Congress abstract – 19th Congress of the European Society for Organ Transplantation, Denmark. Transplant Int (2019) 32 8(Suppl 2):131.
57. Nassiri N, Kwan L, Bolagani A, Thomas AG, Sinacore J, Ronin M, et al. The oldest and the coldest shipped living donor kidneys transplanted through kidney paired donation. Am J Transplant (2020) 20:137-44. doi: 10.1111/ajt.15527.
58. Treat EG, Miller ET, Kwan L, Connor SE, Maliski SL, Hicks EM, et al. Outcomes of shipped live donor kidney transplants compared with traditional living donor kidney transplants. Transpl Int (2014) 27:1175-82. doi: 10.1111/tri.12405.
59. Segev DL, Veale JL, Berger JC, Hiller JM, Hanto RL, Leeser DB, et al. Transporting live donor kidneys for kidney paired donation: initial national results. Am J Transplant (2011) 11:356-60. doi: 10.1111/j.1600-6143.2010.03386.x.
60. Allen RDM, Pleass HCC, Woodroffe C, Clayton PA, Ferrari P. Challenges of kidney paired donation transplants involving multiple donor and recipient surgeons across Australia. ANZ J Surg (2018) 88:167-71. doi: 10.1111/ans.13517.
61. Garonzik–Wang JM, Sullivan B, Hiller JM, Cass V, Tchervenkow J, Feldman L, et al. International kidney paired donation. Transplantation (2013) 96:e55-6. doi: 10.1097/TP.0b013e3182a68879.
62. Ashlagi I, Gilchrist DS, Roth AE, Rees MA. Non simultaneous chains and dominos in kidney-paired donation-revisited. Am J Transplant (2011) 11:984-94. doi: 10.1111/j.1600-6143.2011.03481.x.
63. De Klerk M, Van Der Deijl WM, Witvliet MD, Haase-Kromwijk BJ, Claas FH, Weimar W. The optimal chain length for kidney paired exchanges: an analysis of the Dutch program. Transpl Int (2010) 23:1120-5. doi: 10.1111/j.1432-2277.2010.01114.x.

Appendix 1: Bibliographic Searches

7 Definition of sensitization

7.1 Clinical question (1)

In renal transplantation, what is the effect of the number of eplet mismatches (eplet load) on the induction of donor specific antibodies (DSA) after transplantation?

7.2 Search strategy and results

The Transplant Library (www.transplantlibrary.com) was searched from inception to January 11, 2021 using the search strategy below:

1. (eplet or epitope).ti,ab.
2. Epitopes/
3. HLAMatchmaker.ti,ab.
4. or/1-3
5. (“donor specific antibod$” or DSA).ti,ab.
6. 4 AND 5
7. Limit 6 to kidney transplant

This search identified four potentially relevant references.
MEDLINE and EMBASE were searched from inception to January 11, 2021. In order to create a manageable search set, the search criteria above were refined to:

1. (eplet or epitope).ti,ab.
2. Epitopes/
3. HLAMatchmaker.ti,ab.
4. or/1-3
5. (“donor specific antibod$” or DSA).ti,ab.
6. 4 AND 5
7. KIDNEY TRANSPLANTATION/
8. ((kidney or renal) adj5 (graft$ or allograft$ or transplant$)).ti,ab.
9. Organ transplantation/
10. or/6-8
11. 6 and 10

This search yielded 387 potentially relevant references.

1.2 Clinical question (2)

In renal transplantation, what is the clinical relevance of donor specific HLA antibodies present before transplantation, detected by solid phase assays only?

1.3 Search strategy and results

The Transplant Library (www.transplantlibrary.com) was searched from inception to January 8, 2021 using the search strategy below:

1. (“donor specific antibod$” or DSA).ti,ab.
2. Limit 1 to kidney transplant

Searches identified 78 potentially relevant references.

The last systematic review has a search date to 2016. Searches were therefore expanded to include non-randomized studies from 2016 to 8th January 2021 for completeness. We searched MEDLINE and EMBASE from 01/01/2016 to 08/01/2021. In order to create a manageable search set, the search criteria above were refined to:

1. (Luminex or “solid phase”).ti,ab.
2. (“donor specific antibod$” or DSA),ti,ab
3. 1 and 2
4. (pretransplant$ or pre-transplant$),ti,ab.
5. 3 and 4
6. KIDNEY TRANSPLANTATION/
7. ((kidney or renal) adj5 (graft$ or allograft$ or transplant$)).ti,ab.
8. Organ transplantation/
9. or/6-8
10. 5 and 9
11. Limit 9 to yr=”2016-current”

This search yielded 216 potentially relevant references.

2 Comparison of practices across Europe

2.1 Clinical questions

- What are the different approaches to blood group incompatible transplantation in Europe?
- What are the different approaches to HLA antibody incompatible transplantation in Europe?
- Which countries in Europe use desensitization for antibody incompatible living or deceased donor transplantation?
- Which countries in Europe have living donor sharing schemes?
- What desensitization techniques are used in Europe?

2.2 Search strategy and results

The Transplant Library (www.transplantlibrary.com) was searched from inception to January 11, 2021 using the search strategy below:

1. Blood Group Incompatibility/
2. ABO incompatib$.mp.
3. ABOi.ti,ab.
4. HLA incompatib$.mp.
5. incompatible pair$.mp.
6. incompatible don$.mp.
7. Desensitization, Immunologic/
8. (desensitiz$ or desensitis$).ti,ab.
9. kidney chain.mp.
10. kidney exchange.mp.
11. kidney sharing scheme.mp.
12. paired exchange.mp.
13. paired don$.mp.

14. domino chain.mp.
15. sharing registry.mp.
16. or/1-15

Searches identified 48 potentially relevant references.

Bibliographic searches were expanded to include non-randomized studies. We searched MEDLINE and EMBASE on January 11, 2021 using the search strategy below.

1. Blood Group Incompatibility/
2. ABO incompatib$.mp.
3. ABOi.ti,ab.
4. HLA incompatib$.mp.
5. incompatible pair$.mp.
6. incompatible don$.mp.
7. or/1-6
8. Desensitization, Immunologic/
9. (desensitiz$ or desensitis$).ti,ab.
10. kidney chain.mp.
11. kidney exchange.mp.
12. kidney sharing scheme.mp.
13. paired exchange.mp.
14. paired don$.mp.
15. domino chain.mp.
16. sharing registry.mp.
17. or/8-16
18. 7 and 17
19. organ transplantation/
20. kidney transplantation/
21. pancreas transplantation/
22. lung transplantation/
23. heart transplantation/
24. liver transplantation/
25. (pancreas$ transplant$ and kidney$ transplant$).tw.
26. simultaneous pancreas kidney transplant$.tw.
27. spk.tw.
28. lung transplant$.tw.
29. heart transplant$.tw.
30. liver transplant$.tw.
31. solid organ transplant$.tw.
32. kidney transplant$.tw.
33. pancreas transplant$.tw.
34. or/19-33
35. 18 and 34
36. remove duplicates from 35

This search yielded 1043 potentially relevant references.

A separate search was in MEDLINE and EMBASE conducted for Question 2 on January 11, 2021

1. HLA incompatib$.ti,ab.
2. anti-HLA.ti,ab.
3. Desensitization, Immunologic/mt [Methods]
4. (desensitiz$ or desensitis$).ti,ab.
5. transplant$.ti,ab.
6. 1 or 2
7. 3 or 4
8. 5 and 6 and 7
9. limit 8 to yr="2010 - 2021"

The search yielded 474 references.
All search results were limited to studies published from 2010 and only publications from one of the European countries were included. Non-English studies were excluded.

2 How can we risk stratify patients

Searches were conducted by authors

3 Desensitization strategies

Searches were conducted by authors

4 Outcomes after HLA-incompatible kidney transplantation

4.1 Clinical question

What is the outcome of HLA-incompatible transplantation compared to 'conventional' transplantation or dialysis?

4.2 Search strategy and results

The Transplant Library (www.transplantlibrary.com) was searched from inception to January 28, 2021 using the search strategy below:

1. HLA incompatib$.ti,ab.
2. anti-HLA.ti,ab.
3. HLA abs.mp.
4. HLA antibod$.ti,ab.
5. incompatible kidney.ti,ab.
6. DSA.ti,ab.
7. donor specific antibodies.ti,ab.
8. or/1-7
9. limit 8 to kidney transplant

Searches identified 111 potentially relevant references.
MEDLINE and EMBASE were searched from inception to January 28, 2021 using the search strategy below:

1. HLA incompatib$.ti,ab.
2. positive crossmatch.ti,ab.
3. ((kidney or renal) adj3 (transplant$ or graft$)).ti,ab.
4. 1 or 2
5. 3 and 4

This search yielded 736 potentially relevant references.

2 Kidney sharing schemes for sensitized patients

2.1 Clinical questions

- In HLA incompatible renal transplant recipients, is desensitization better than kidney paired donation (KPD)?
- In KPD programs, should ABO incompatible pairs be included?
- In KPD programs, should compatible pairs be included?
- What is the impact of prolonged cold ischemia time and logistical issues in transnational KPD?
- Are there immunological advantages for sensitized patients in transnational KPD versus national/local programs?

2.2 Search strategy and results

The Transplant Library (www.transplantlibrary.com) was searched from inception to November 5, 2020 using the search strategy below:

1. kidney chain.mp.
2. kidney exchange.mp.
3. kidney sharing scheme.mp.
4. paired exchange.mp.
5. kidney paired donation.mp.
6. domino chain.mp.
7. paired don$.mp.
8. Blood Group Incompatibility/
9. ABO incompatible.mp.
10. HLA incompatible.mp.
11. incompatible pair$.mp.
12. incompatible don$.mp.
13. (sensitiz$ or sensitis$).ti,ab.
14. or/1-13
15. limit 14 to kidney transplant

Searches identified 25 potentially relevant references.

MEDLINE and EMBASE were searched from inception to November 7, 2020 using the search strategy below:

1. kidney chain.mp.
2. kidney exchange.mp.
3. kidney sharing scheme.mp.
4. paired exchange.mp.
5. paired don$.mp.
6. domino chain.mp.
7. or/1-6
8. Blood Group Incompatibility/
9. ABO incompatible.mp.
10. HLA incompatible.mp.
11. incompatible pair$.mp.

12. incompatible don$.mp.
13. Desensitization, Immunologic/
14. (sensitiz$ or sensitis$).ti,ab.
15. or/8-14
16. living donors/
17. (liv$ adj3 kidney$).tw.
18. (liv$ adj5 donor$).tw.
19. or/16-18
20. 7 and 15 and 19

This search yielded 597 potentially relevant references.

For PICO questions 4 and 5 the search terms for KPD were combined with the additional terms below (search was run on November 17, 2020):

1. international cooperation/
2. Europe/
3. (transnational or international or multinational or European or global or world or cross$ border$).ti,ab.

This search yielded 109 potentially relevant references.

Appendix 2: Attendees (both in person and via live-stream) at the expert working group meeting to discuss the recommendations, Milan, Italy, August 28th 2021.

**Presenters:**	**Country**
Nizam Mamode	UK
Frans Claas	The Netherlands
Sian Griffin	UK
Lucrezia Furian	Italy
Oriol Bestard	Spain
Christophe Legendre	France
**Participants:**	
Cristiano Amarelli	Italy
Lionel Couzi	France
Emanuele Cozzi	Italy
Marta Crespo	Spain
Aiko De Vries	The Netherlands
Annelies de Weerd	The Netherlands
Isabelle Delabaye	Belgium
Dimitrios Moutzouris	UK
Alexander-Farsad Eskandary	Austria
Denis Glotz	France
Fadi Haidar	Switzerland
Uwe Heemann	Germany
Jelena Stojanovic	UK
Karine Hadaya	Switzerland
Christian Kjellman	Sweden
Fiona Loud	UK
Soufian Meziyerh	The Netherlands
Liset Pengel	UK
Lionel Rostaing	France
Soeren Schwartz	Denmark
Olivier Thaunat	France
Nicholas Torpey	UK
Fabio Vistoli	Italy
Gianluigi Zaza	Italy
